# New 3-Acyl Tetramic Acid Derivatives from the Deep-Sea-Derived Fungus *Lecanicillium fusisporum*

**DOI:** 10.3390/md20040255

**Published:** 2022-04-06

**Authors:** Xinya Xu, Yanhui Tan, Chenghai Gao, Kai Liu, Zhenzhou Tang, Chunju Lu, Haiyan Li, Xiaoyong Zhang, Yonghong Liu

**Affiliations:** 1 Institute of Marine Drugs, Guangxi University of Chinese Medicine, Nanning 530200, China; xyxu@gxtcmu.edu.cn (X.X.); gaoch@gxtcmu.edu.cn (C.G.); liuk@gxtcmu.edu.cn (K.L.); tangzz@gxtcmu.edu.cn (Z.T.); luchunjv@stu.gxtcmu.edu.cn (C.L.); lihaiyan12368@163.com (H.L.); 2 State Key Laboratory for the Chemistry and Molecular Engineering of Medicinal Resources, School of Chemistry and Pharmaceutical Science, Guangxi Normal University, Guilin 541004, China; tyh533@smu.edu.cn; 3 College of Marine Sciences, South China Agricultural University, Guangzhou 510642, China

**Keywords:** tetramic acid derivatives, deep-sea fungus, clavipitacae, *Lecanicillium fusisporum*, anti-inflammatory activity

## Abstract

Seven rare C3-C6 reduced 3-acyl tetramic acid derivatives, lecanicilliumins A–G (**1**–**7**), along with the known analogue cladosporiumin D (**8**), were obtained from the extract of the deep-sea-derived fungus *Lecanicillium fusisporum* GXIMD00542 within the family Clavipitacae. Their structures were elucidated by extensive spectroscopic data analysis, quantum chemistry calculations and chemical reaction. Compounds **1**, **2**, **5**–**7** exhibited moderate anti-inflammatory activity against NF-κB production using lipopolysaccharide (LPS) induced RAW264.7 cells with EC_50_ values range of 18.49–30.19 μM.

## 1. Introduction

**Figure 1:**
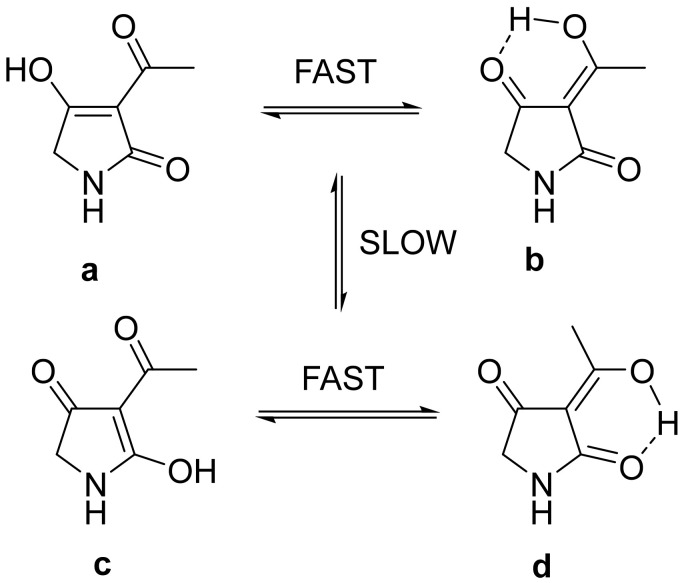
The interconverting internal tautomers of 3-acyl tetramic acid [5].

Tetramic acid (2,4-pyrrolidinedione) derivatives have been isolated from many terrestrial and marine organisms, including bacteria, fungi and sponges [1]. They have attracted considerable attention from chemists, biologists and physicians for their diverse chemical structures and broad range of potent biological activities such as antimicrobial, antitumoral, antiprotozoal, protease-inhibitory and anti-inflammatory effects [2,3,4]. Among these tetramate derivatives, the 3-acyl tetramic acids, which contain an acyl substituent at C-3, are the most common ones found in nature [4]. The 3-acyl tetramic acids have two sets of rapidly interconverting internal tautomers **a**/**b** and **c**/**d** in solution (Figure 1). Distribution calculation of these four tautomers indicated the *exo*-enol **d** with C3-C6 olefinic bone, is the main tautomeric form and commonly reported [5,6,7]. The C3-C6 reduced 3-acyl tetramic acid derivatives are rarely found [8].

The fungal genus *Lecanicillium*, which once was placed in the genus *Verticillium* [9], belongs to the family Clavipitacae. *Lecanicillium* spp. such as *L. fusisporum*, *L. psalliotae* and *L. lecanii* are well known as entomopathogenic species and used for biological control of insect pests [10,11,12]. However, the secondary metabolisms of these fungi are rarely reported. Ishidoh et al. has isolated two new cyclic lipodepsipeptides verlamelins A and B from *Lecanicillium* sp. HF627 [13]. Huang et al. reported one new alkaloid lecasporinoid from marine fungal strain *L. fusisporum* [14]. Unfortunately, these compounds did not show identified biological activities. During our continuing search on bioactive compounds from marine fungi, the deep-sea-derived fungal strain GXIMD00542 has been isolated from Mariana Trench sediment (141°57′ E, 10°51′ N, 5467 m depth) and identified as *L. fusisporum* by ITS rDNA sequence homology. Further chemical study led to the isolation of eight 3-acyl tetramic acid derivatives including seven new compounds and one known analogue cladosporiumin D (**8**) [8]. This is the first report of tetramic acid derivatives obtained from the genus *Lecanicillium.* Some of them exhibited moderate anti-inflammatory activity against NF-κB production using LPS-induced RAW264.7 cells. Herein, we report the isolation, structural elucidation and the biological determination of these compounds.

## 2. Results and Discussion

**Figure 2:**
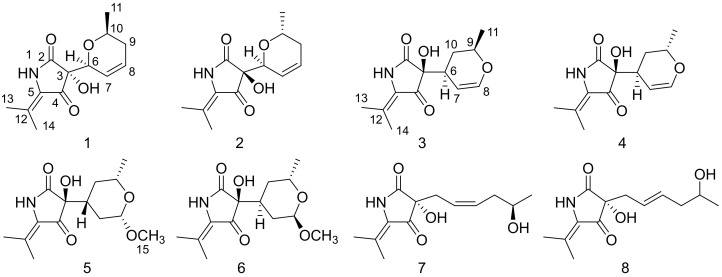
The chemical structures of compounds **1**–**8**.

The deep-sea-derived fungal strain GXIMD00542 was inoculated and fermented using liquid medium in standing situation for 30 days at 25 °C. The HPLC-UV guided isolation of the extract led to compounds **1**–**8** (Figure 2).

**Figure 3:**
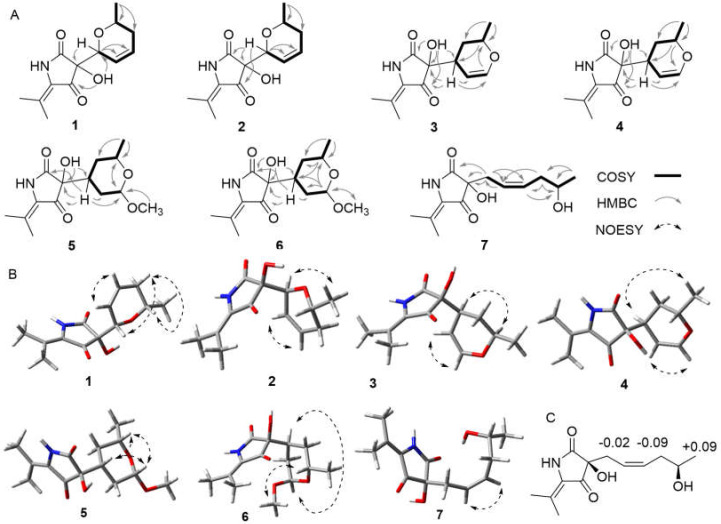
(**A**) Key ^1^H-^1^H COSY and HMBC correlations for compounds **1**–**7**. (**B**) Key NOESY correlations for compounds **1**–**7**. (**C**) The Δ*δ* (*δ*_S_−*δ*_R_) values for MTPA esters of **7**.

**Figure 4:**
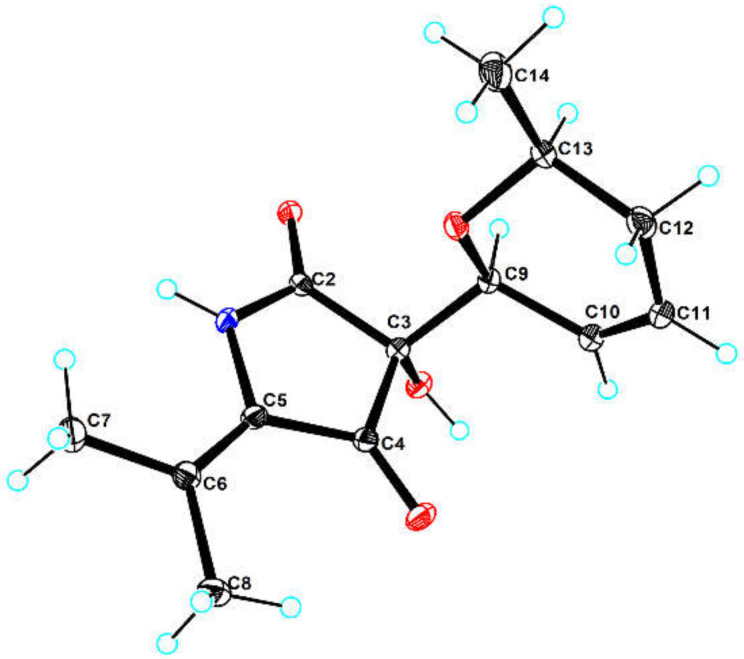
X-ray ORTEP drawing of compound **1**. The crystal structure was deposited at the Cambridge Crystallographic Data Centre with number of CCDC 2092073.

Compound **1** was obtained as light brown orthorhombic crystal. The molecular formula of C_13_H_17_NO_4_ was established by HRTOFESIMS quasi-molecular ion at *m*/*z* 274.1063 [M + Na]^+^ (calcd. 274.1055) (Figure S8), which had six degrees of unsaturation. The 1D NMR and HSQC spectral signals revealed the presence of three methyl groups, one methylene, four methines including two oxygenated methines and one double bone, and four quaternary carbons including one amide carbonyl and one α, β-unsaturated ketone moiety (Table 1 and Table 2). The HMBC correlations from N*H*-1 to C-2, C-3, C-4, C-12, and from C*H*_3_-13/C*H*_3_-14 to C-5, C-12 indicated the existence of the 5-isobutenyl-2,4-pyrrolidinedione moiety, which is structural core of tetramic acid derivative (Figure 3) [8]. ^1^H-^1^H COSY correlations of H-6/H-7/H-8/H-9/H-10/C*H*_3_-11, and HMBC correlations from H-6 to C-7, C-8, from H-9 to C-7, C-8, C-10, from C*H*_3_-11 to C-9, C-10, and an extra degree of unsaturation indicated the presence of a 2-methyl-3,6-dihydro-2*H*-pyran moiety. HMBC correlations from H-6 to C-3 and C-4, from O*H*-3 to C-6 declared the 2-methyl-3,6-dihydro-2*H*-pyran moiety attached to the C-3 of the 5-isobutenyl-2,4-pyrrolidinedione moiety. The NOESY correlations between H-6 and H-9α, between H-9α and H-10 indicated H-6 and H-10 on the same side of the 2-methyl-3,6-dihydro-2*H*-pyran moiety. The absolute configuration of **1** was further determined as (3*R*,6*S*,10*S*) by Cu Kα radiation X-ray crystal analysis, which was obtained as orthorhombic crystal in methanol (crystal size: 0.16 × 0.08 × 0.08 mm^3^, Flack parameter: 0.03) (Figure 4).

**Figure 5:**
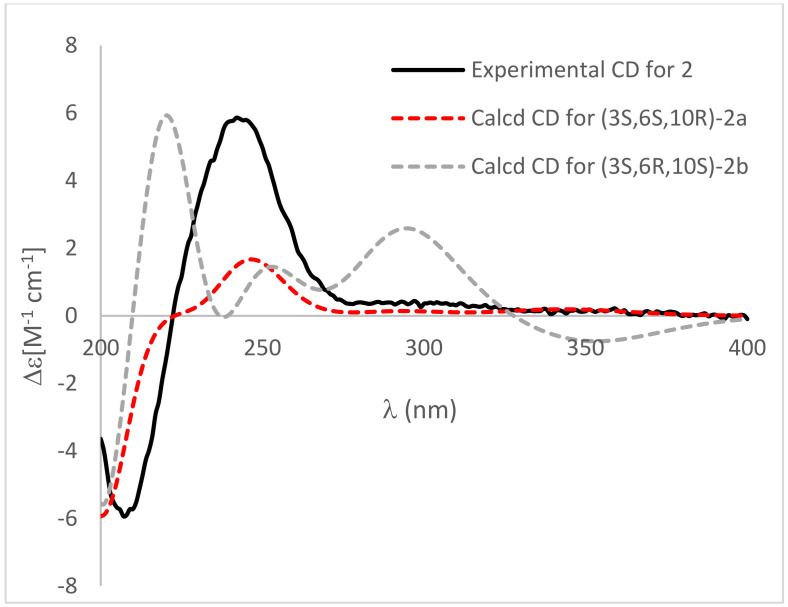
Comparison of calculated ECD spectra of (3*S*,6*S*,10*R*)-**2a** (red), (3*S*,6*R*,10*S*)-**2b** (gray) in MeOH and experimental CD (black). σ = 0.3 eV, UV shift = 7 nm.

Compound **2** was yielded as light brown powder, with the molecular formula C_13_H_17_NO_4_, based on the HRTOFESIMS spectrum (*m*/*z* 274.1059 [M + Na]^+^) (Figure S15). The same molecular formula and detailed analysis of the NMR data disclosed that **2** had same plane structure with **1**. The relative configurations of 2-methyl-3,6-dihydro-2*H*-pyran moiety of **2** were elucidated by the NOESY correlations between H-6 and C*H*_3_-10. Based on the comparison of ECD cotton effects with **1** and similar known compound **8** [8], the absolute configuration of C-3 was assigned as 3*S*, in which (3*S*)-5-isobutenyl-2,4-pyrrolidinedione showed negative cotton effects around 210 nm and positive cotton effects around 230 nm, whereas (3*R*)-5-isobutenyl-2,4-pyrrolidinedione exhibited opposite cotton effects at the same wavelength. The further ECD calculation of the optimized conformations of (3*S*,6*S*,10*R*)-**2a** and (3*S*,6*R*,10*S*)-**2b** were conducted at the M062X/def2TZVP level. Accordingly, the absolute configurations of **2** were assigned as (3*S*,6*S*,10*R*)-**2** by the comparison of calculated ECD curves with experimental ECD spectrum (Figure 5).

Compound **3** was white amorphous powder with the same molecular formula as **1** and **2** by the HRTOFESIMS spectrum (*m*/*z* 252.1241 [M + H]^+^) (Figure S22). The ^1^H NMR and ^13^C NMR data revealed **3** had high structural similarities to **1** and **2** (Table 2 and Table 3) except for the chemical shift of the dihydro-2*H*-pyran moiety. The ^1^H-^1^H COSY correlations H-8/H-7/H-6/H-10/H-9/C*H*_3_-11 and HMBC correlations from H-6 to C-7, C-8, C-10, from H-8 to C-6, C-7, C-9 and from H-9 to C-6, C-10, C-11 deduced the presence of 2-methyl-3,4-dihydro-2*H*-pyran moiety. The HMBC correlations from H-6 to C-2, C-3, and C-4 indicated that the 5-isobutenyl-2,4-pyrrolidinedione moiety was attached to γ-position of the 2-methyl-3,4-dihydro-2*H*-pyran moiety. The NOESY correlations between H-6 and H-9 indicated they were on the same plane of the pyran moiety. The absolute configuration of C-3 was assigned as 3*S* on the basis of the same ECD cotton effects around 210 nm and 230 nm as compound **2**. The two candidate isomers (3*S*,6*S*,9*R*)-**3a** and (3*S*,6*R*,9*S*)-**3b** were further calculated by the quantum chemical calculations of the NMR data (qccNMR) at the B97-2/pcSseg-1 level. DP4+ analysis identified (3*S*,6*S*,9*R*)-**3** as the most probable structure with 89.66% DP4+ probability [15,16].

**Figure 6:**
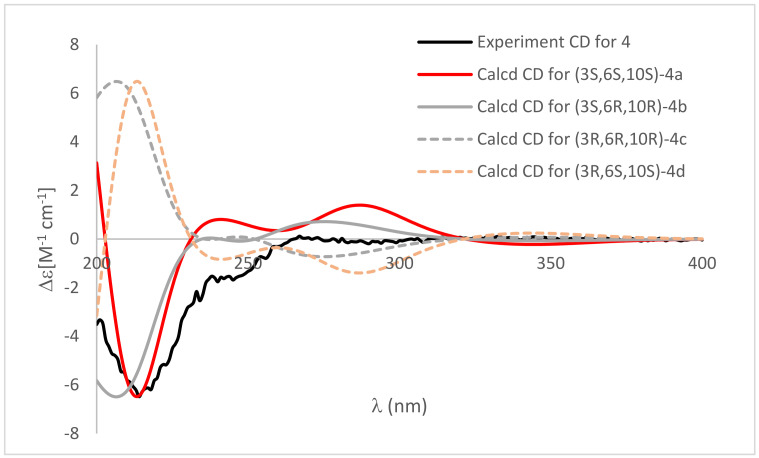
Comparison of calculated ECD spectra of (3*S*,6*S*,9*S*)-**4a** (red), (3*S*,6*R*,9*R*)-**4b** (gray), (3*R*,6*R*,9*R*)-**4c** (dashed gray) and (3*R*,6*S*,9*S*)-**4d** (dashed orange) and experimental CD (black). σ = 0.3 eV; UV shift = 7 nm.

Compound **4** shared the same plane structure as **3** on the basis of HRTOFESIMS data (*m*/*z* 252.1235 [M + H]^+^) and 1D NMR data (Table 2 and Table 3). The NOESY correlation between H-6 and C*H*_3_-11 disclosed they were on the same plane of the 5-isobutenyl-2,4-pyrrolidinedione moiety. The absolute configuration of C-3 was assigned as 3*S* based on the negative cotton effect around 210 nm and the ECD calculation of (3*S*,6*S*,9*S*)-**4a**,(3*S*,6*R*,9*R*)-**4b**, (3*R*,6*R*,9*R*)-**4c** and (3*R*,6*S*,9*S*)-**4d** at the M062X/def2TZVP level (Figure 6). Further, the qccNMR calculation and the DP4+ analysis of (3*S*,6*S*,9*S*)-**4a** and (3*S*,6*R*,9*R*)-**4b** at the B97-2/pcSseg-1 level identified (3*S*,6*S*,9*S*)-**4** as the most probable structure with 91.53% DP4+ probability [15,16].

The molecular formula C_14_H_21_NO_5_ of **5** was deduced from the HRTOFESIMS data (*m*/*z* 306.1319 [M + Na]^+^). The 1D NMR data showed that **5** had 5-isobutenyl-2,4-pyrrolidinedione moiety (Table 2 and Table 3). The ^1^H-^1^H COSY correlations of H-8/H-7/H-6/H-10/H-9/C*H*_3_-11, and HMBC correlations from H-8 to C-6, C-7, C-9, C-15, from H-9 to C-8 and from OC*H*_3_-15 to C-8 disclosed the presence of 2-methoxy-6-methyltetrahydro-2*H*-pyran moiety. HMBC correlations from H-6 to C-2, C-3, C-4 and from O*H*-3 to C-6 indicated the 5-isobutenyl-2,4-pyrrolidinedione moiety was linked to the 2-methoxy-6-methyltetrahydro-2*H*-pyran moiety at C-6. The relative configurations of **5** was elucidated by the NOESY spectra and 1D selective NOESY spectra. NOESY correlations between H-6 and H-9, and 1D selective NOESY correlations between H-6 and H-8, H-9, between H-8 and H-6, H-9 exhibited that H-6, H-8 and H-9 were at the same plane of the 2-methoxy-6-methyltetrahydro-2*H*-pyran moiety. The absolute configuration of C-3 was assigned as *S* according to the similar cotton effects tendency as compounds **2**–**4**. The qccNMR calculation and the DP4+ analysis of (3*S*,6*R*,8*S*,9*S*)-**5a** and (3*S*,6*S*,8*R*,9*R*)-**5b** at the B97-2/pcSseg-1 level showed (3*S*,6*R*,8*S*,9*S*)-**5** as the most probable structure with 86.42% DP4+ probability [15,16].

Compound **6** had the same plane structure as **5** according to the identical HRTOFESIMS data (*m*/*z* 306.1319 [M + Na]^+^) and similar 1D NMR data. The observed NMR chemical shift difference between **6** and **5** of H-6, H-8, and C-6, C-8 and C-9 indicated they were diastereoisomers. NOESY correlations between H-6 and H-8, between H-9 and OC*H*_3_-15 disclosed H-6, H-8, and C*H*_3_-11 were on the same plane of the 2-methoxy-6-methyltetrahydro-2*H*-pyran moiety, while H-9 and OC*H*_3_-15 were on the other side. The absolute configuration of C-3 was also assigned as *S* because of the similar cotton effects tendency as compounds **2**–**5**. The qccNMR calculation and the DP4+ analysis of (3*S*,6*S*,8*R*,9*S*)-**6a** and (3*S*,6*R*,8*S*,9*R*)-**6b** at the B97-2/pcSseg-1 level showed (3*S*,6*S*,8*R*,9*S*)-**6** as the most probable structure with 86.61% DP4+ probability [15,16].

Compound **7** was obtained as light-yellow powder. The HRTOFESIMS ion signal at *m*/*z* 276.1203 [M + Na]^+^ disclosed the molecular formula C_13_H_19_NO_4_. The 1D NMR data showed that **1** was tetramic acid derivative too. The ^1^H-^1^H COSY correlations of H-6/H-7/H-8/H-9/H-10/C*H*_3_-11, and HMBC correlations from H-6 to C-7, C-8, from H-9 to C-7, C-8, C-10, from C*H*_3_-11 to C-9, C-10 indicated **7** had the same plane structure as known compond **8**. The double bond *Z*-Δ^7,8^ configuration of **7** was assigned by the coupling constant value *J*_H-7,8_ = 10.9 Hz instead of *J*_H-7,8_ = 15.0 Hz in **8** (*E*-Δ^7,8^) [8]. The configuration of C-3 and C-10 were assigned as (3*S*,10*R)* based on the comparison of ECD cotton effect with **1**–**6** and the chemical shift values analysis of Mosher ester derivatives of **7**, respectively (Figure 3C).

To access the anti-inflammatory effects of these C3-C6 reduced 3-acyl tetramic acid derivatives, all the compounds were evaluated for their inhibition of NF-κB production using LPS induced RAW264.7 cells. Compounds **1**, **2**, **5**–**7** exhibited moderate effect with EC_50_ values of 18.49 ± 1.21, 25.81 ± 1.30, 23.10 ± 1.26, 24.70 ± 1.19, and 26.52 ± 1.12 μM, respectively. Meanwhile, the positive control BAY11-7082 (NF-κB inhibitor) had the EC_50_ values of 1.5 ± 1.4 μM.

The anti-inflammatory effects of tetramic acid derivatives have been reported. Vermelhotin, pseurotin A3 and pseurotin G showed anti-inflammatory effect for the inhibition of NO production in LPS-induced RAW264.7 cells with EC_50_ values of 5.35 μM, 34.5 μM and 57.0 μM, respectively [17,18].

Their cytotoxicity was also determined towards hepatocytes cell line LO2 and hepatoma cells line Bel-7402 and RAW264.7. No definite inhibitory effects were observed at the concentration of 30 μM.

## 3. Materials and Methods

### 3.1. General Experimental Procedure

Circular dichroism and optical rotations were measured with JASCO J-1500 circular dichroism spectrophotometer (JASCO, Easton, PA, USA). ^1^H NMR, ^13^C NMR and 2D NMR spectra were recorded on a Bruker AVANCE III HD600 spectrometer (Bruker, Billerica, MA, USA) with TMS as reference. The suitable crystal was analyzed on a Bruker Smart Apex II single crystal X-Ray diffractometer (Bruker, Billerica, MA, USA). High-resolution TOFESIMS was performed on a WATERS Xevo G2-S Qtof Quadrupole Time-of-Flight Mass Spectrometry (Waters, Milford, MA, USA). Analysis and semi-preparative reversed-phase HPLC was performed on a Shimadzu LC-2030 liquid chromatography (Shimadzu, Kyoto, Japan) with YMC-Pack ODS-A column 250 × 10 mm i.d., S-5 μm × 12 nm, and 250 × 20 mm i.d., S-5 μm × 12 nm. Column chromatography (CC) was performed on silica gel (200–300 mesh, Jiangyou Silica Gel Co., Ltd., Yantai, China) or CHROMATOREX C18 silica (Fuji Silysia Chemical Ltd., Kozoji-cho, Kasugai Aichi, Japan).

### 3.2. Fungal Material

The fungal strain GXIMD00542 was isolated from Mariana Trench sediment (141°57′ E, 10°51′ N, 5467 m depth), and identified as *Lecanicillium fusisporum* by ITS rDNA sequence homology (97.57% similarity with *L. fusisporum*) (GenBank accession number ON005314, Figure S1). The strain was deposited in the Guangdong Microbial Culture Collection Center (GDMCC) with the accession number 62091.

### 3.3. Fermentation and Extraction

Spores of the fungal strain were inoculated into 1000 mL Erlenmeyer flasks each containing 300 mL of liquid medium (glucose 1%, maltose 2%, monosodium glutamate 1%, KH_2_PO_4_ 0.05%, MgSO_4_ 7H_2_O 0.003%, yeast extract 0.3%, dissolved in artificial sea water. After 30 days of stationary cultivation at 25 °C, the whole broths (30 L) were filtered through cheesecloth. Sterilized XAD-16 resin (20 g/L) was added to the liquor and shaken at low speed for 30 min to absorb the organic products. The resin was washed with distilled water to remove medium residue then eluted with methanol. The methanol solvent was removed under vacuum to yield a brown residue (42 g). The mycelium portion was smashed and extracted twice with 80% acetone/H_2_O. The acetone soluble fraction was dried in vacuo to yield 18 g of residue. The residues of liquor and mycelium extracts were combined together according to TLC chromatography detecting.

### 3.4. Isolation and Purification

The combined extract (60 g) was subjected to silica gel column (1000 g) and eluted with CH_2_Cl_2_/acetone (100:0–60:40, *v*/*v*) to yield 15 fractions (Fractions 1–15). Fraction 9 (0.41 g) was separated by C18 silica column and eluted with CH_3_CN/H_2_O (10:90–40:60, *v*/*v*) to give 20 sub-fractions (Fr. 9-1–9-20). Sub-fraction 9-11 was subjected to semi-preparation HPLC (CH_3_CN/H_2_O, 18:82) at the flow rate of 3 mL/min to obtain **5** (t_R_ 30.3 min, 8 mg). Sub-fraction 9-13 was purified with semi-preparation HPLC (MeOH/H_2_O, 40:60) at the flow rate of 3 mL/min to yield **6** (t_R_ 28.9 min, 11 mg). Sub-fraction 9-14 was purified with semi-preparation HPLC (MeOH/H_2_O, 40:60) at the flow rate of 3 mL/min to yield **3** (t_R_ 28.9 min, 40 mg) and **4** (t_R_ 33.6, 14 mg). Sub-fraction 9-15 was purified with semi-preparation HPLC (CH_3_CN/H_2_O, 22:78) at the flow rate of 3 mL/min to yield **2** (t_R_ 35.8 min, 8 mg) and **1** (t_R_ 38.2 min, 5 mg). Fraction 13 (2.0 g) was isolated with C18 silica column eluting with CH_3_CN/H_2_O (10:90–40:60) to obtain 17 sub-fractions (Fractions 13-1–13-17). Sub-fraction 13-11 was purified by preparatory HPLC (MeOH/H_2_O, 30:70) at the flow rate of 5 mL/min to yield **8** (t_R_ 96.5 min, 22 mg) and **7** (t_R_ 106.1 min, 10 mg).

Lecanicilliumin A (**1**): light brown orthorhombic crystal; [α]^25^_D_ −34.7 (*c* 0.09, MeOH); UV (MeOH) λ_max_ (log ε) 235 (3.75), 294 (3.28) nm; CD (MeOH) λ_max_ (Δε) 233 (–4.05), 266 (−0.21), 280 (−0.40), 347 (+0.71); ^1^H and ^13^C NMR data, see Table 1 and Table 2; HRESIMS *m*/*z* 274.1063 [M + Na]^+^ (calcd. for 274.1055).

Lecanicilliumin B (**2**): light brown powder; [α]^25^_D_ +14.4 (*c* 0.80, MeOH); UV (MeOH) λ_max_ (log ε) 236 (3.78), 296 (3.31) nm; CD (MeOH) λ_max_ (Δε) 207 (−5.93), 242 (+5.87), 297 (+0.44); ^1^H and ^13^C NMR data, see Table 1 and Table 2; HRESIMS *m*/*z* 274.1059 [M + Na]^+^ (calcd. for 274.1055).

Lecanicilliumin C (**3**): white amorphous powder; [α]^25^_D_ +43.2 (*c* 0.94, MeOH); UV (MeOH) λ_max_ (log ε) 233 (4.06), 296 (3.66) nm; CD (MeOH) λ_max_ (Δε) 216 (−3.94), 240 (+6.53), 281 (+1.84); ^1^H and ^13^C NMR data, see Table 2 and Table 3; HRESIMS *m*/*z* 252.1241 [M + H]^+^ (calcd. for 252.1236).

Lecanicilliumin D (**4**): white amorphous powder; [α]^25^_D_ −18.9 (*c* 0.41, MeOH); UV (MeOH) λ_max_ (log ε) 234 (3.98), 296 (3.60) nm; CD (MeOH) λ_max_ (Δε) 214 (−6.48), 239 (−1.60), 245 (−1.68), 269 (+0.03); ^1^H and ^13^C NMR data, see Table 2 and Table 3; HRESIMS *m*/*z* 252.1235 [M + H]^+^ (calcd. for 252.1236).

Lecanicilliumin E (**5**): colorless solid; [α]^25^_D_ +12.0 (*c* 0.27, MeOH); UV (MeOH) λ_max_ (log ε) 233 (3.89), 296 (3.50) nm; CD (MeOH) λ_max_ (Δε) 207 (−6.95), 234 (+1.80), 256 (+0.05), 303 (+0.95); ^1^H and ^13^C NMR data, see Table 2 and Table 3; HRESIMS *m*/*z* 306.1319 [M + Na]^+^ (calcd. for 306.1317).

Lecanicilliumin F (**6**): colorless solid; [α]^25^_D_ −65.9 (*c* 0.23, MeOH); UV (MeOH) λ_max_ (log ε) 233 (3.99), 296 (3.60) nm; CD (MeOH) λ_max_ (Δε) 206 (−8.38), 233 (+1.44), 251 (−0.20), 300 (+1.14); ^1^H and ^13^C NMR data, see Table 2 and Table 3; HRESIMS *m*/*z* 306.1319 [M + Na]^+^ (calcd. for 306.1317).

Lecanicilliumin G (**7**): light yellow solid; [α]^25^_D_ −4.5 (*c* 0.90, MeOH); UV (MeOH) λ_max_ (log ε) no obvious absorption peak in the 200–400 range; CD (MeOH) λ_max_ (Δε) 207 (−1.60), 234 (+2.18), 331 (−0.37); ^1^H and ^13^C NMR data, see Table 1 and Table 2; HRESIMS *m*/*z* 276.1203 [M + Na]^+^ (calcd. for 276.1212).

### 3.5. Mosher’s Ester Reaction

The Mosher’s ester reaction of **7** was performed as described in the literature [19]. Briefly, 1 mg of dried **7** was dissolved in 0.5 mL anhydrous dichloromethane. Then, 10 μL of triethylamine, 0.1 mg of 4-dimethyl aminopyridine (DMAP) and 10 uL (*R*)-(−)- or (*S*)-(+)-methoxy-α-(trifluoromethyl)phenylacetyl chloride (MTPA-Cl) were added, respectively. The mixtures were stirred at room temperature overnight and quenched by adding water to give the (*S*)- and (*R*)-MTPA ester derivatives of **7**.

### 3.6. X-ray Crystal Structure Analysis

Compound **1** was obtained as light brown orthorhombic crystal from MeOH with molecular formula of C_26_H_36_N_2_O_9_. The suitable crystal was selected and analyzed on a CCD area detector diffractometer (Bruker Smart Apex II) using Cu Kα radiation. The crystallographic data for 1 (CCDC 2092073) was deposited in the Cambridge Crystallographic Data Centre.

Crystal data for lecanicilliumin A (**1**): C_26_H_36_N_2_O_9_ (*M* = 520.57), orthorhombic, space group P2_1_2_1_2_1_, *a* = 11.32560(10) Å, *b* = 12.19970(10) Å, *c* = 18.5233(2) Å, α = 90°, β = 90°, γ = 90°, *V* = 2559.34(4) Å^3^, *Z* = 4, ρ_calc_ = 1.351 g/cm^3^, *μ* (Cu Kα) = 0.851 mm^−1^, crystal size: 0.16 × 0.08 × 0.08 mm^3^, 14,388 reflections measured (8.678° ≤ θ ≤ 148.214°), 5046 unique (*R*_int_ = 0.0182, *R*_sigma_ = 0.0192). The final *R*_1_ was 0.0266 (*I* > 2σ(I) and ω*R*_2_ was 0.0702, flack = 0.03 (4).

### 3.7. Computational Methods

Molecular Merck force field (MMFF) and DFT/TDDFT calculations were performed with Spartan’14 software package (Wavefunction Inc., Irvine, CA, USA) [20] and Gaussian16 program package (Gaussian Inc., Wallingford, CT, USA) [21], respectively, using default grids and convergence criteria. MMFF conformational search generated low-energy conformers within a 10 kcal/mol energy window were subjected to geometry optimization using the B3LYP/6-31G(d,p) method. Frequency calculations were performed with the same method to verify each optimized conformer was a true minimum and to estimate their relative thermal free energies (ΔG) at 298.15 K. Energies of the low-energy conformers in MeOH were calculated at the M062X/def2-TZVP level. Solvent effects were taken into account by using a polarizable continuum model (PCM). The TDDFT calculations were performed using the M062X functions with basis set def2-TZVP. The number of excited states per each molecule was 32. The ECD spectra were generated by the program SpecDis [22] using a Gaussian band shape from dipole-length dipolar and rotational strengths. Equilibrium population of each conformer at 298.15 K was calculated from its relative free energies using Boltzmann statistics. The calculated spectra were generated from the low-energy conformers according to the Boltzmann weighting of each conformer in a MeOH solution.

The B3LYP/6-31G(d) optimized geometries of **3**–**6** were adopted for further NMR computation. Gauge-Independent Atomic Orbital (GIAO) calculations of the ^13^C NMR chemical shifts were accomplished by DFT at the B97-2/pcSseg-1 level in DMSO with PCM. The calculated ^13^C NMR spectroscopic data were averaged according to the Boltzmann distribution by the program Multiwfn 3.7 [23]. The ^13^C NMR chemical shifts for TMS were calculated by the same procedure and used as the reference.

### 3.8. Cytotoxicity Assay

Cytotoxicities of **1**–**8** were evaluated against LO2, Bel-7402 and RAW264.7 cell lines using MTT method. The detailed methodologies for cytotoxic testing have been described in previous report [24].

### 3.9. NF-κB Luciferase Assay

The effects of **1**–**8** on LPS-induced NF-κB luciferase activity were detected by luciferase reporter gene assay as described earlier. Briefly, RAW264.7 cells, stably transfected with a NF-κB luciferase reporter construct, were treated with **1**–**8** (30 μM) and BAY11-7082 (NF-κB inhibitor, 5 μM) for 4 h, followed by stimulation with LPS (100 ng/mL^−1^) for 6 h. The luciferase activity was determined using the luciferase assay system (Promega, Madison, WI, USA) [24]. The dose-dependent effects of **1**–**8** and BAY11-7082 on LPS induced NF-κB luciferase activity were detected by the same assay.

## 4. Conclusions

In conclusion, a series of rare C3-C6 reduced 3-acyl tetramic acid derivatives, lecanicilliumins A-G (**1**–**7**), along with the known analogue cladosporiumin D (**8**), were obtained from the extract of the deep-sea-derived fungus *Lecanicillium fusisporum* GXIMD00542. Compounds **1**, **2**, **5**–**7** exhibited moderate anti-inflammatory activity against NF-κB production using LPS induced RAW264.7 cells with EC_50_ values range of 18.49–30.19 μM. This finding expanded the chemical diversity of 3-acyl tetramic acid derivatives, and also enriched the secondary metabolites in deep-sea-derived fungus.

## Figures and Tables

**Table 1:** ^1^H NMR data (600 MHz) for compounds **1**, **2** and **7** in DMSO-*d*_6_ (δ in ppm, *J* in Hz).

No.	1	2	7
6	4.30, br s	4.23, m	2.41, dd, (14.1, 7.5)
7	5.97, d, (10.4)	5.95, m	5.17, dt, (10.9, 7.3)
8	5.82, dtd, (10.2, 2.1, 5.7)	5.79, dtd, (9.9, 4.3, 1.9)	5.49, dt, (10.9, 7.3)
9	1.90, m, H-9α	1.93, m,	1.94–2.04, ovp.
	1.65, m, H-9β	1.74, ovp. ^a^,	
10	3.52, ddd, (15.6, 6.1, 4.6)	3.52, dtt, (12.3, 6.1, 3.5)	3.52, m
11	0.99, d, (6.1)	0.99, d, (6.1)	0.98, d, (6.2)
13	1.77, s	1.77, s	1.78, s
14	2.02, s	2.07, s	2.06, s
N*H*-1	10.33, br s	10.19, br s	10.34, br s
O*H*-3	6.32, br s	6.35, br s	6.14, br s
O*H*-13	-	-	4.44, d, (4.5)

^a^ overlapped.

**Table 2:** ^13^C NMR data (150 MHz) for compounds **1**–**7** in DMSO-*d*_6_ (*δ* in ppm).

No.	1	2	3	4	5	6	7
2	171.45, C	169.7, C	171.35, C	171.89, C	171.51, C	171.53, C	171.91, C
3	74.31, C	74.22, C	75.14, C	75.85, C	75.29, C	75.55, C	73.96, C
4	197.12, C	199.36, C	199.04, C	199.64, C	199.59, C	199.65, C	199.38, C
5	120.24, C	120.52, C	122.3, C	122.07, C	122.44, C	122.28, C	122.47, C
6	76.97, CH	77.68, CH	37.73, CH	34.26, CH	39.6, CH	35.23, CH	33.46, CH_2_
7	125.37, CH	125.44, CH	97.81, CH	96.65, CH	30.31, CH_2_	28.86, CH_2_	121.66, CH
8	124.85, CH	124.85, CH	145.33, CH	145.25, CH	101.66, CH	97.05, CH	130.96, CH
9	32.02, CH_2_	32.02, CH_2_	71.2, CH	68.28, CH	69.93, CH	63.82, CH	36.74, CH_2_
10	69.62, CH	69.73, CH	29.7, CH_2_	27.95, CH_2_	31.72, CH_2_	31.85, CH_2_	65.81, CH
11	21.4, CH_3_	21.35, CH_3_	21.17, CH_3_	20.63, CH_3_	21.45, CH_3_	21.6, CH_3_	23.11, CH_3_
12	128.96, C	129.13, C	128.57, C	128.6, C	128.5, C	128.44, C	128.41, C
13	20.57, CH_3_	20.57, CH_3_	20.71, CH_3_	19.91, CH_3_	20.69, CH_3_	20.66, CH_3_	20.69, CH_3_
14	18.39, CH_3_	18.39, CH_3_	18.49, CH_3_	18.44, CH_3_	18.46, CH_3_	18.43, CH_3_	18.46, CH_3_
15	-	-	-	-	55.32, CH_3_	53.67, CH_3_	-

**Table 3:** ^1^H NMR data (600 MHz) for compounds **3**–**6** in DMSO-*d*_6_ (δ in ppm, *J* in Hz).

No.	3	4	5	6
6	2.64, ddt, (11.4, 5.8, 2.0)	2.45, ddd, (10.5, 4.7, 2.4)	1.98, tt, (12.5, 3.7)	2.17, tt, (12.8, 3.5)
7	4.51, d, (6.4)	4.44, dd, (6.4, 3.4)	1.59, dt, (12.5, 2.1)	1.56, ovp.
			1.05, td, (12.5, 9.5)	1.05, ovp.
8	6.35, dd, (6.4, 2.3)	6.38, dd, (6.4, 2.2)	4.23, dd, (9.5, 2.1)	4.66, d, 2.6
9	3.85, dqd, (12.6, 6.3, 1.7)	3.91, ddd, (11.5, 6.3, 3.2)	3.41, dqd, (12.3, 6.1, 1.9)	3.63, dqd, (12.2, 6.3, 2.1)
10	1.78, ovp. ^a^	2.12, ddd, 1(4.2, 7.0, 3.5)	1.52, m	1.56, ovp.
	1.53, dt, (13.2, 11.4)	1.45, ddd, (14.2, 7.0, 3.5)	0.97, td, (12.7, 11.0)	1.05, ovp.
11	1.18, d, (6.3)	1.13, d, (6.3)	1.11, d, (6.1)	1.05, d, (6.3)
13	1.81, s	1.78, s	1.80 s	1.79, s
14	2.09, s	2.05, s	2.08, s	2.07, s
15	-	-	3.30, s	3.19, s
N*H*-1	10.37, br s	10.41, br s	10.4, br s	10.37, br s
O*H*-3	6.09, br s	6.07, br s	6.04, br s	5.97, br s

^a^ overlapped.

## Data Availability

Data are contained within the article.

## Supplementary Materials

The following supporting information can be downloaded at: https://www.mdpi.com/article/10.3390/md20040255/s1, Figure S1: The colonies and the ITS rRNA sequences data of deep-sea-derived fungus *Lecanicillium fusisporum* GXIMD00542; Figures S2–S52: The 1D and 2D NMR spectra, HRTOFESIMS spectra of compound **1**–**7**; Table S1: Crystal data and structure refinement for compound **1**. Figure S53: ^1^H NMR spectrum of (*R*)-MTPA ester of 7; Figure S54: ^1^H NMR spectrum of (*S*)-MTPA ester of 7; Tables S2–S13: Relative free energies and equilibrium populations of conformers for **2**–**6**; Figures S55 and S56: The optimized structures and the calculated CD spectra of conformers for **2** in MeOH at M06-2X/def2TZVP level; Figures S57–S74: The optimized structure of conformers, correlation plots of experimental ^13^C NMR chemical shifts versus the corresponding calculated data and DP4+ probabilities (%) for conformers of **3**–**6**. File S1: Crystallographic Data of compound **1**. References [2,25] are cited in the Supplementary Materials
