# Supplementary material for: New 3-Acyl Tetramic Acid Derivatives from the Deep-Sea-Derived Fungus Lecanicillium fusisporum
Source: Mar Drugs. 2022 Apr 6;20(4):255. doi: 10.3390/md20040255 (PMC9031249; doi:10.3390/md20040255)
Supplement: Supplementary file 1 [file marinedrugs-20-00255-s001.zip › marinedrugs-1654401-SI0405.pdf]

Supporting Information

New 3-Acyl Tetramic acid Derivatives from the Deep-Sea-Derived Fungus *Lecanicillium fusisporum*

Xinya Xu [1,†], Yanhui Tan [2,†], Chenghai Gao [1], Kai Liu[1], Zhenzhou Tang [1], Chunju Lu [1], Haiyan Li [1], Xiaoyong Zhang [3,*] and Yonghong Liu [1,*]

[1] Institute of Marine Drugs, Guangxi University of Chinese Medicine, Nanning 530200, P. R. China; xyxu@gxtcmu.edu.cn (X. X.); gaoch@gxtcmu.edu.cn (C. G); liuk@gxtcmu.edu.cn (K. L.); tangzz@gxtcmu.edu.cn (Z. T.); luchunjv@stu.gxtcmu.edu.cn (C. L.); lihaiyan12368@163.com (H. L.); yonghongliu@scsio.ac.cn (Y. L.)

[2] School of Chemistry and Pharmaceutical Science, State Key Laboratory for the Chemistry and Molecular Engineering of Medicinal Resources, Guangxi Normal University, Guilin 541004, P. R. China; tyh533@smu.edu.cn

[3] College of Marine Sciences, South China Agricultural University, Guangzhou 510642, P. R. China; zhangxiaoyong@scau.edu.cn

* Correspondence: zhangxiaoyong@scau.edu.cn; yonghongliu@scsio.ac.cn

† These authors contributed equally to this paper.

Table of Contents

Figure S1. The colonies and the ITS rRNA sequences data of deep-sea-derived fungus *Lecanicillium fusisporum* GXIMD00542 4
Figure S2. ^{1}H NMR spectrum of 1 4
Figure S3. ^{13}C NMR spectrum of 1 5
Figure S4. HSQC spectrum of 1 5
Figure S5. ^{1}H-^{1}H COSY spectrum of 1 6
Figure S6. HMBC spectrum of 1 6
Figure S7. NOESY spectrum of 1 7
Figure S8. HR-ESI-MS spectrum of 1 7
Table S1. Crystal data and structure refinement for compound 1 8
Figure S9. ^{1}H NMR spectrum of 2 9
Figure S10. ^{13}C NMR spectrum of 2 9
Figure S11. HSQC spectrum of 2 10
Figure S12. ^{1}H-^{1}H COSY spectrum of 2 10
Figure S13. HMBC spectrum of 2 11
Figure S14. NOESY spectrum of 2 11
Figure S15. HR-ESI-MS spectrum of 2 12
Figure S16. ^{1}H NMR spectrum of 3 12
Figure S17. ^{13}C NMR spectrum of 3 13
Figure S18. HSQC spectrum of 3 13
Figure S19. ^{1}H-^{1}H COSY spectrum of 3 14
Figure S20. HMBC spectrum of 3 14
Figure S21. NOESY spectrum of 3 15
Figure S22. HR-ESI-MS spectrum of 3 15
Figure S23. ^{1}H NMR spectrum of 4 16
Figure S24. ^{13}C NMR spectrum of 4 16
Figure S25. HSQC spectrum of 4 17
Figure S26. ^{1}H-^{1}H COSY spectrum of 4 17
Figure S27. HMBC spectrum of 4 18
Figure S28. NOESY spectrum of 4 18
Figure S29. HR-ESI-MS spectrum of 4 19
Figure S30. ^{1}H NMR spectrum of 5 19
Figure S31. ^{13}C NMR spectrum of 5 20
Figure S32. HSQC spectrum of 5 20
Figure S33. ^{1}H-^{1}H COSY spectrum of 5 21
Figure S34. HMBC spectrum of 5 21
Figure S35. NOESY spectrum of 5 22
Figure S36. 1D selective NOESY spectrum of 5 22
Figure S37. 1D selective NOESY spectrum of 5 23
Figure S38. HR-ESI-MS spectrum of 5 23
Figure S39. ^{1}H NMR spectrum of 6 24
Figure S40. ^{13}C NMR spectrum of 6 24

Figure S41. HSQC spectrum of 6 25
Figure S42. ^{1}H-^{1}H COSY spectrum of 6 25
Figure S43. HMBC spectrum of 6 26
Figure S44. NOESY spectrum of 6...26
Figure S45. HR-ESI-MS spectrum of 6 27
Figure S46. ^{1}H NMR spectrum of 7...27
Figure S47. ^{13}C NMR spectrum of 7..28
Figure S48. HSQC spectrum of 7 28
Figure S49. ^{1}H-1H COSY spectrum of 7..29
Figure S50. HMBC spectrum of 7 29
Figure S51. NOESY spectrum of 7...30
Figure S52. HR-ESI-MS spectrum of 7 30
Figure S53. ^{1}H NMR spectrum of (*R*)-MTPA ester of 7 31
Figure S54. ^{1}H NMR spectrum of (*S*)-MTPA ester of 7..31
Table S2. Relative free energies[a] and equilibrium populations[b] of conformers for (3*S*, 6*S*, 10*R*)-2a [c] 31
Figure S55. The optimized structures (left) and the calculated CD spectra of conformers (3*S*, 6*S*, 10*R*)-2a in MeOH at M06-2X/def2TZVP level (right). σ=0.30 eV. 34
Table S3. Relative free energies[a] and equilibrium populations[b] of conformers for (3*S*, 6*R*, 10*S*)-2b[c] 34
Figure S56. The optimized structures (left) and the calculated CD spectra of conformers (3*S*, 6*R*, 10*S*)-2b in MeOH at M06-2X/def2TZVP level (right). σ=0.30 eV..35
Table S4. Relative free energies[a] and equilibrium populations[b] of conformers for (3*S*, 6*S*, 9*R*)-3a[c] 35
Figure S57. The optimized structure of conformers for (3*S*, 6*S*, 9*R*)-3a in MeOH at B3LYP/6-31G (d) level 36
Table S5. Relative free energies[a] and equilibrium populations[b] of conformers for (3*S*, 6*R*, 9*S*)-3b[c] 36
Figure S58. The optimized structure of conformers for (3*S*, 6*R*, 9*S*)-3b in MeOH at B3LYP/6-31G (d) level 37
Figure S59. Correlation plots of experimental ^{13}C NMR chemical shifts versus the corresponding calculated data for (3*S*, 6*S*, 9*R*)-3a and (3*S*, 6*R*, 9*S*)-3b 38
Figure S60. DP4+ probabilities (%) for conformers (3*S*, 6*S*, 9*R*)-3a (isomer 1) and (3*S*, 6*R*, 9*S*)-3b (isomoer 2) 38
Table S6. Relative free energies[a] and equilibrium populations[b] of conformers for (3*S*,6*S*,9*S*)-4a[c] .38
Figure S61. The optimized structures (left) and the calculated CD spectra for conformers (3*S*,6*S*,9*S*)-4a in MeOH at M06-2X/def2TZVP level (right). σ=0.30 eV. 40
Table S7. Relative free energies[a] and equilibrium populations[b] of conformers for (3*S*,6*R*,9*R*)-4b[c] 40
Figure S62. The optimized structures (left) and the calculated CD spectra for conformers (3*S*,6*R*,9*R*)-4b in MeOH at M06-2X/def2TZVP level (right). σ=0.30 eV. 41
Table S8. Relative free energies[a] and equilibrium populations[b] of conformers for (3*R*,6*R*,9*R*)-4c[c] 41
Figure S63. The optimized structures (left) and the calculated CD spectra for conformers (3*R*,6*R*,9*R*)-4c in MeOH at M06-2X/def2TZVP level (right). σ=0.30 eV. 43
Table S9. Relative free energies[a] and equilibrium populations[b] of conformers for (3*R*,6*S*,9*S*)-4d[c].43

Figure S64. The optimized structures (left) and the calculated CD spectra for conformers (3*R*,6*S*,9*S*)-4d in MeOH at M06-2X/def2TZVP level (right). σ=0.30 eV. 44
Figure S65. Correlation plots of experimental ^{13}C NMR chemical shifts versus the corresponding calculated data for (3*S*, 6*S*, 9*S*)-4a and (3*S*, 6*R*, 9*R*)-4b 45
Figure S66. DP4+ probabilities (%) for conformers of (3*S*, 6*S*, 9*S*)-4a (isomer 1) and (3*S*, 6*R*, 9*R*)-4b (isomoer 2). 46
Table S10. Relative free energies[a] and equilibrium populations[b] of conformers for (3*S*, 6*R*, 8S, 9*S*)-5a[c] 46
Figure S67. The optimized structure of conformers for (3*S*,6*R*,8*S*,9*S*)-5a in MeOH at B3LYP/6-31G (d) level....47
Table S11. Relative free energies[a] and equilibrium populations[b] of conformers for (3*S*,6*S*,8*R*,9*R*)-5b [c] 47
Figure S68. The optimized structure of conformers for (3*S*,6*S*,8*R*,9*R*)-5b in MeOH at B3LYP/6-31G (d) level....48
Figure S69. Correlation plots of experimental ^{13}C NMR chemical shifts versus the corresponding calculated data for (3*S*,6*R*,8*S*,9*S*)-5a and (3*S*,6*S*,8*R*,9*R*)-5b. 48
Figure S70. DP4+ probabilities (%) for conformers of (3*S*,6*R*,8*S*,9*S*)-5a (isomer 1) and (3*S*,6*S*,8*R*,9*R*)-5b (isomoer 2). 49
Table S12. Relative free energies[a] and equilibrium populations[b] of conformers for (3*S*,6*S*,8*R*,9*S*)-6a [c]....49
Figure S71. The optimized structure of conformers for (3*S*,6*S*,8*R*,9*S*)-6a in MeOH at B3LYP/6-31G (d) level....50
Table S13. Relative free energies[a] and equilibrium populations[b] of conformers for (3*S*,6*R*,8*S*,9*R*)-6b [c] 50
Figure S72. The optimized structure of conformers for (3*S*,6*R*,8*S*,9*R*)-6b in MeOH at B3LYP/6-31G (d) level....51
Figure S73. Correlation plots of experimental ^{13}C NMR chemical shifts versus the corresponding calculated data for (3*S*,6*S*,8*R*,9*S*)-6a and (3*S*,6*R*,8*S*,9*R*)-6b....51
Figure S74. DP4+ probabilities (%) for conformers of (3*S*,6*S*,8*R*,9*S*)-6a (isomer 1) and (3*S*,6*R*,8*S*,9*R*)-6b (isomoer 2). 52
Figure S74. Proposed biosynthetic pathway of compounds 1–7. 52

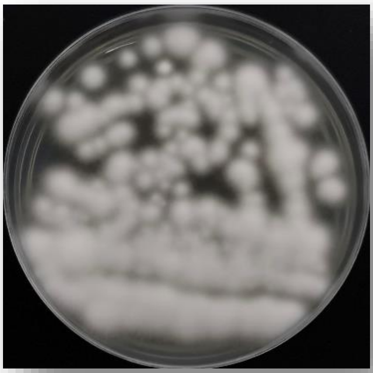

CAACCCTTATGTGACATACCAATCGTTGCTTCGGCGGACTCGCCCCGGTGTCCGGCAGGCCCTCGCGG
CCGGCCGCGACCCGGATCCAGGCGGACGCCGGAGACCATCCAAAAACTCTTTGTATTCTAGCAAGTCT
TCTGAATGAGCCGCAAGGCAACACAAATGAATCAAAACTTTCAACAACGGATCTCTTGGTTCTGGCATC
GATGACTAGCGCAGCGAAAGGAGATAACTAATGTGAATTGCACACATCCGCGAACTTGAACACACTGG
AGGCCCATAGCGGTCCCAACCAATCGCCCCGGTTGAGGAGGACGGGCAACGACTTGATTCGTGGCTA
CCATGCAGGCACCCTTTTCGGCCGTGAAGCTTCACCCACAACTTGCATTCAAAGACTCGATGGTTCCCG
GGATTCTGCAATTCGCTCATGATTGACATGACTTCAGCATTATTTAGCTTTGTACTGTAATGAAAAAGG
AAAGGAACGTTTAATTTATTGGGCTTGAAAATCCGATCAATACTCCACTCTTTTATTTCCCAAAAAAGAG
AATACTACTTGTATATAGTTACA

Figure S1. The colonies and the ITS rRNA sequences data of deep-sea-derived fungus *Lecanicillium fusisporum* GXIMD00542

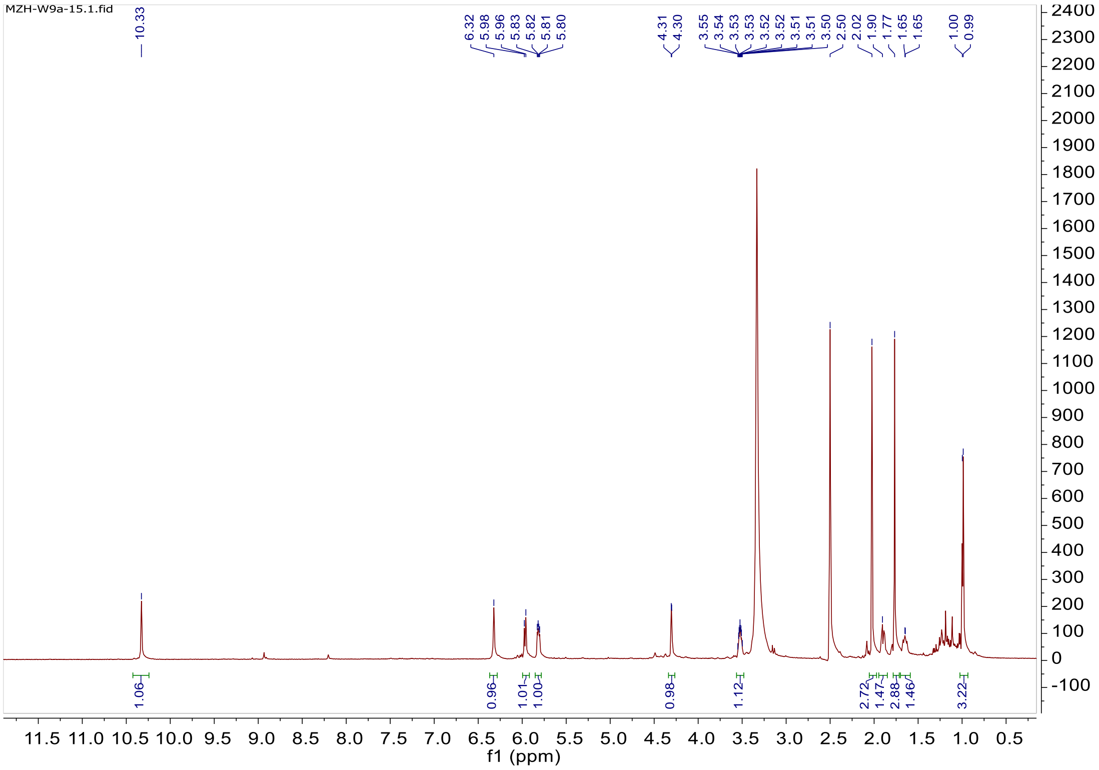

Figure S2. ^{1}H NMR spectrum of **1**

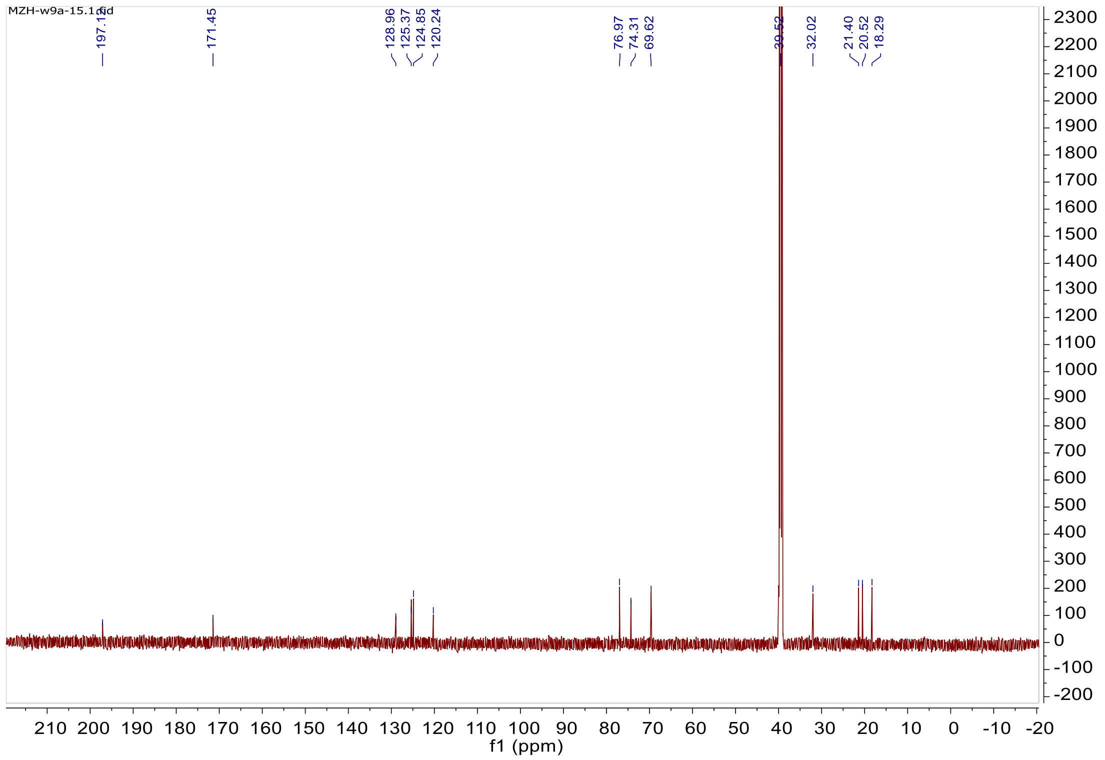

Figure S3. ^{13}C NMR spectrum of **1**

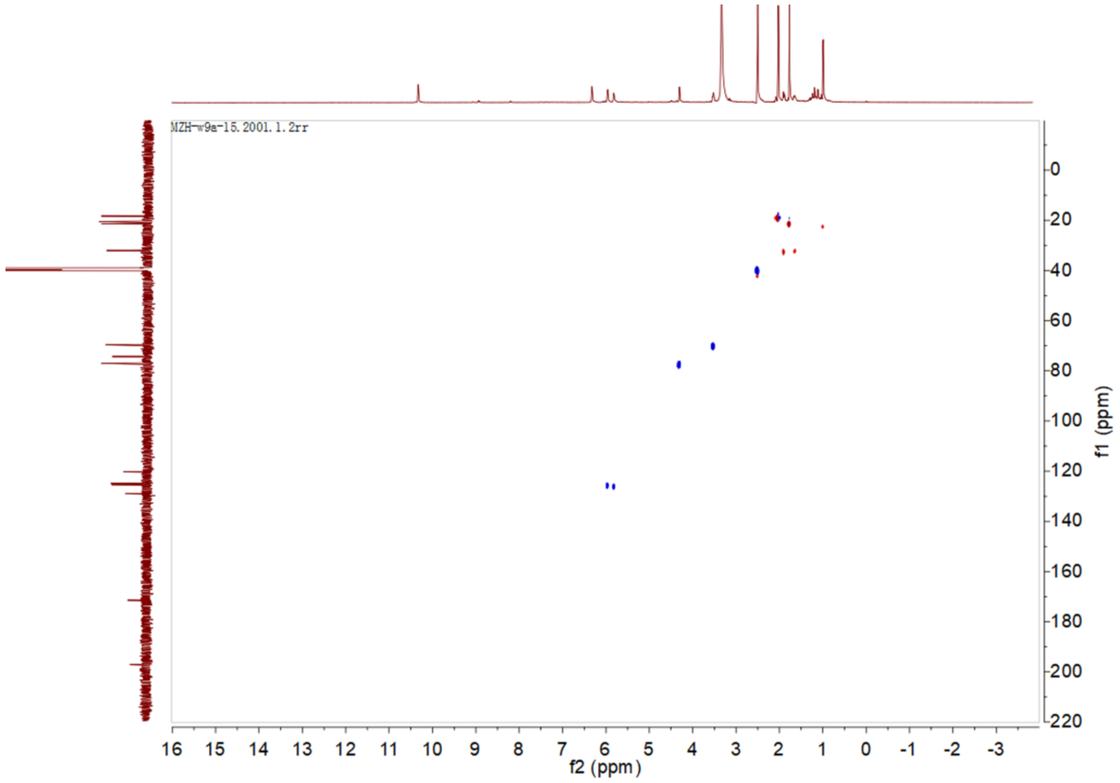

Figure S4. HSQC spectrum of **1**

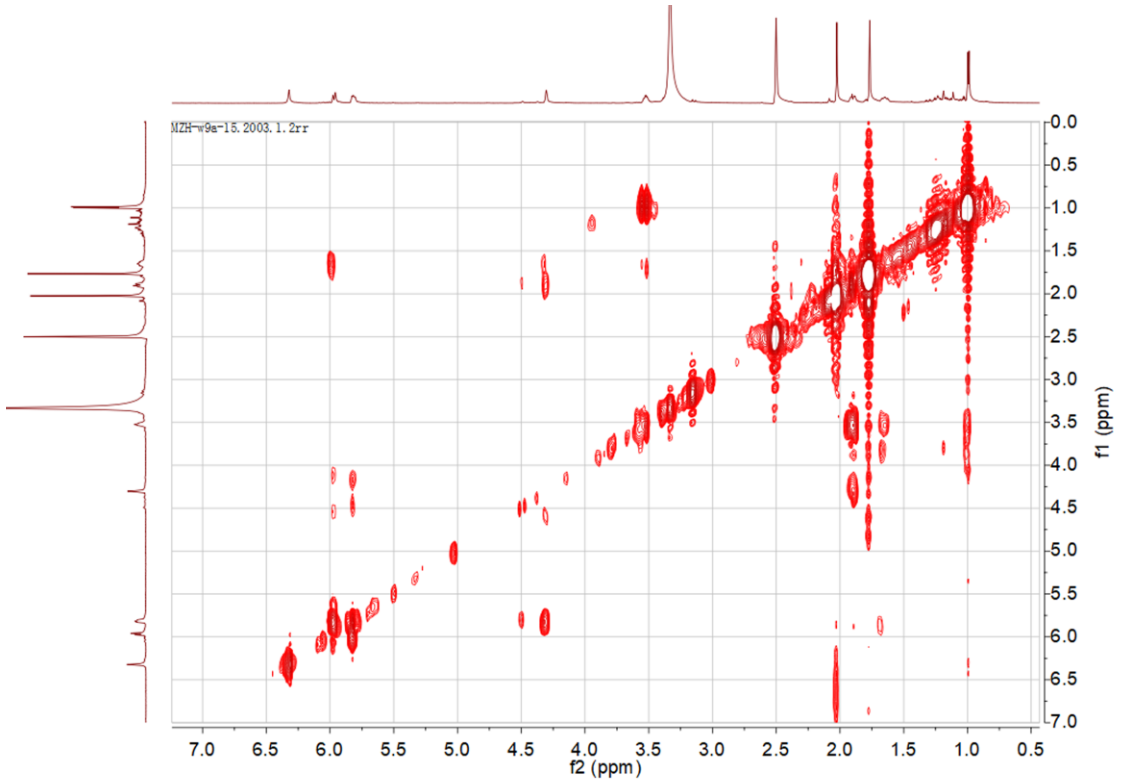

Figure S5. ^{1}H-^{1}H COSY spectrum of **1**

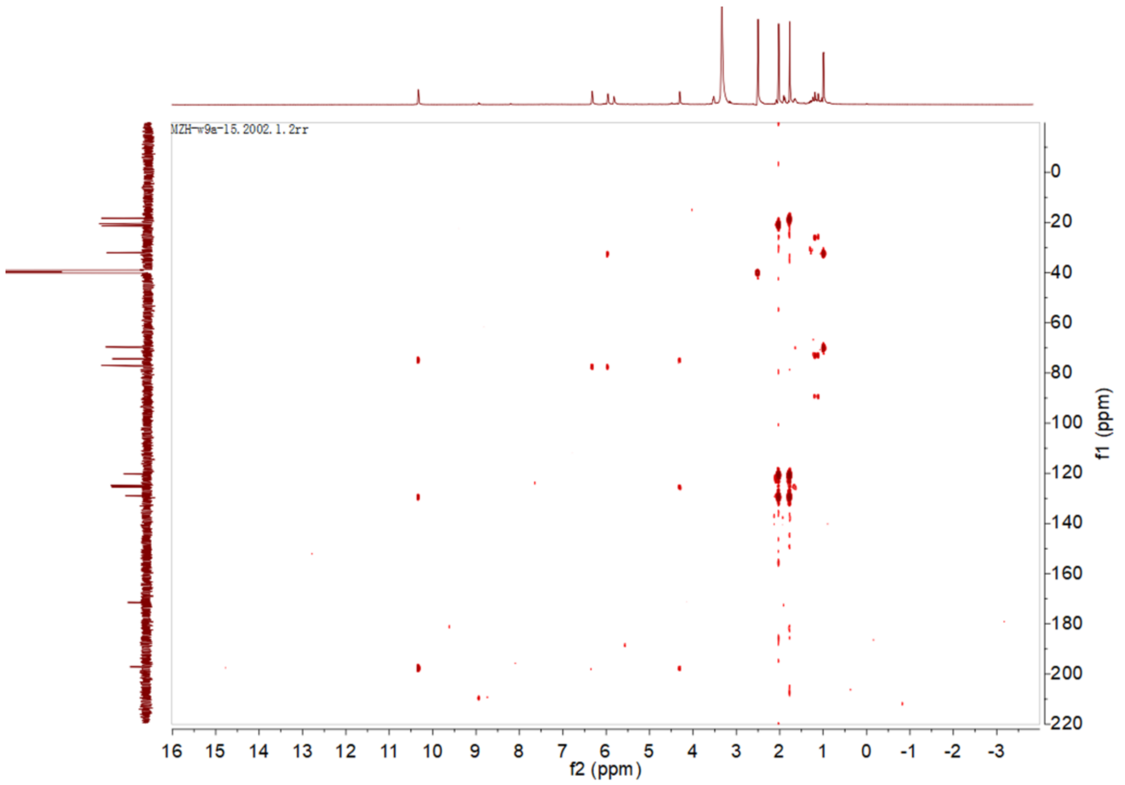

Figure S6. HMBC spectrum of **1**

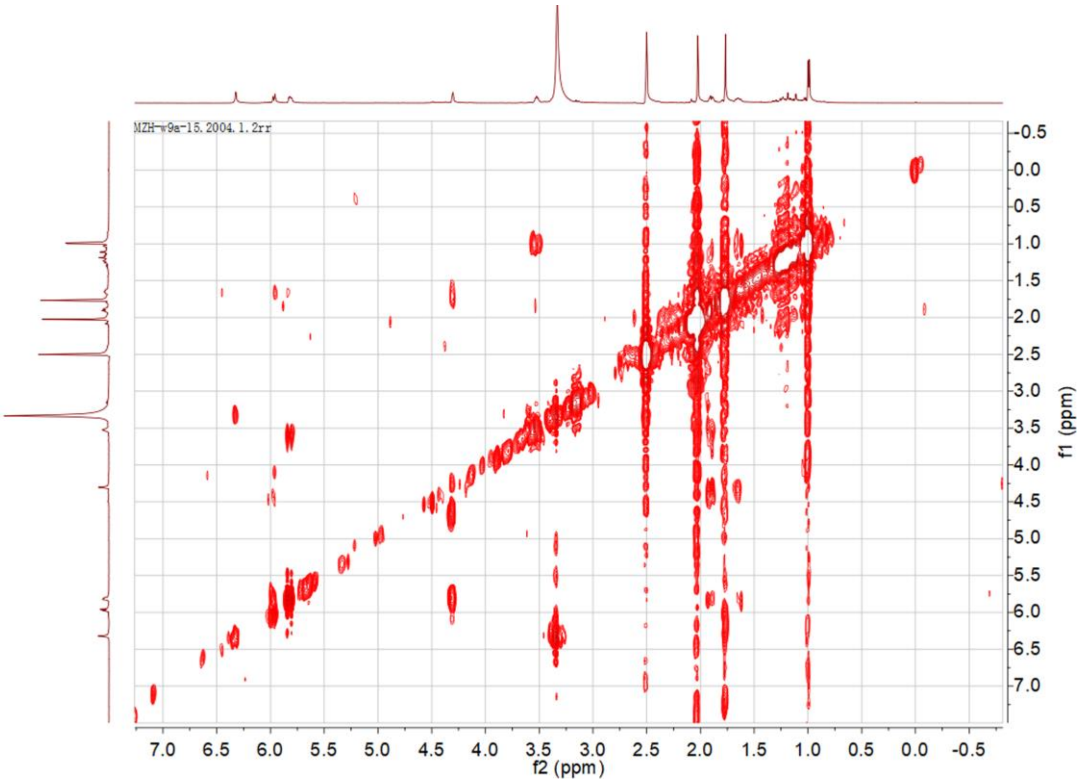

Figure S7. NOESY spectrum of **1**

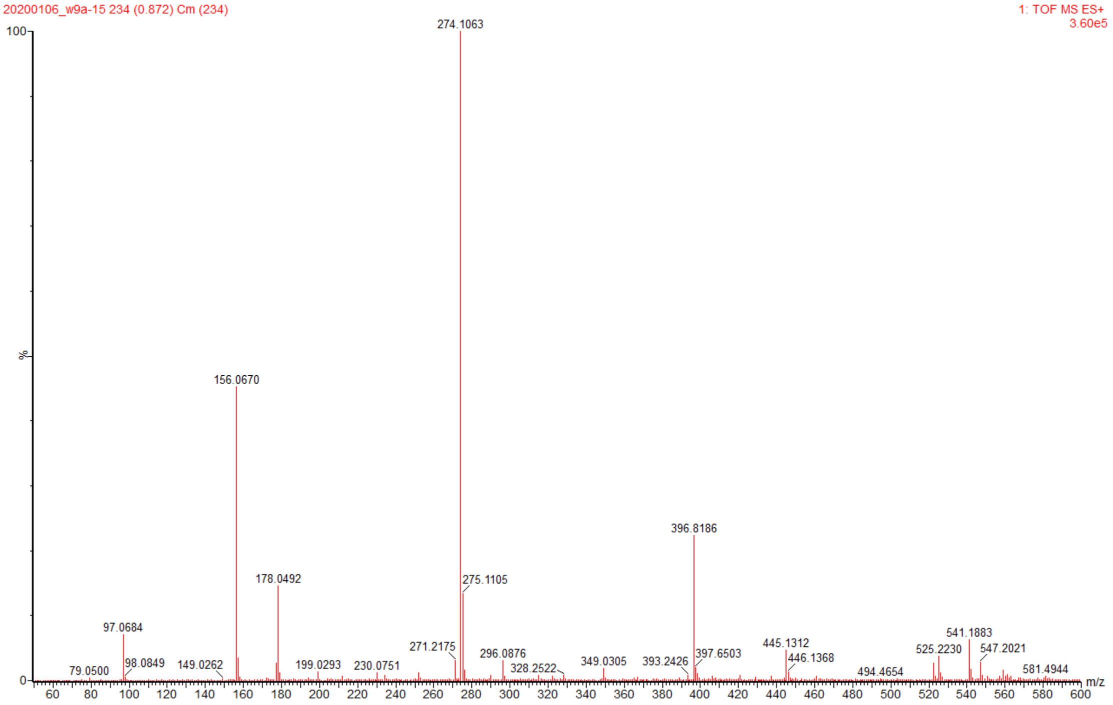

Figure S8. HR-ESI-MS spectrum of **1**

Table S1. Crystal data and structure refinement for compound **1**

Empirical formula	$C_{26}H_{36}N_2O_9$
Formula weight	520.57
Temperature/K	100.00(10)
Crystal system	orthorhombic
Space group	$P2_12_12_1$
a/Å	11.32560(10)
b/Å	12.19970(10)
c/Å	18.5233(2)
α/°	90
β/°	90
γ/°	90
Volume/Å^3	2559.34(4)
Z	4
ρ_{calc}g/cm^3	1.351
μ/mm^{-1}	0.851
F(000)	1112.0
Crystal size/mm^3	0.16 × 0.08 × 0.08
Radiation	Cu Kα (λ = 1.54184)
2Θ range for data collection/°	8.678 to 148.214
Index ranges	-13 ≤ h ≤ 12, -13 ≤ k ≤ 14, -22 ≤ l ≤ 22
Reflections collected	14388
Independent reflections	5046 [R_{int} = 0.0182, R_{sigma} = 0.0192]
Data/restraints/parameters	5046/0/345
Goodness-of-fit on F^2	1.045
Final R indexes [I>=2σ (I)]	R_1 = 0.0266, wR_2 = 0.0702
Final R indexes [all data]	R_1 = 0.0270, wR_2 = 0.0705
Largest diff. peak/hole / e Å^{-3}	0.18/-0.27
Flack parameter	0.03(4)

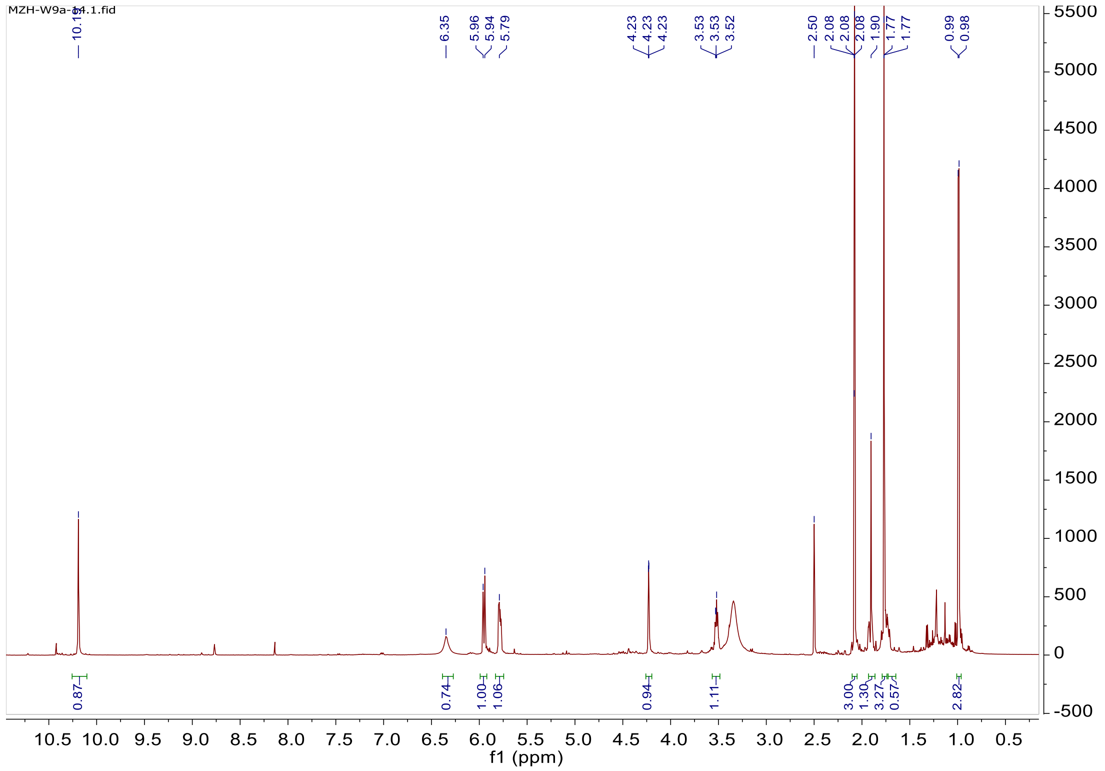

Figure S9. ^{1}H NMR spectrum of **2**

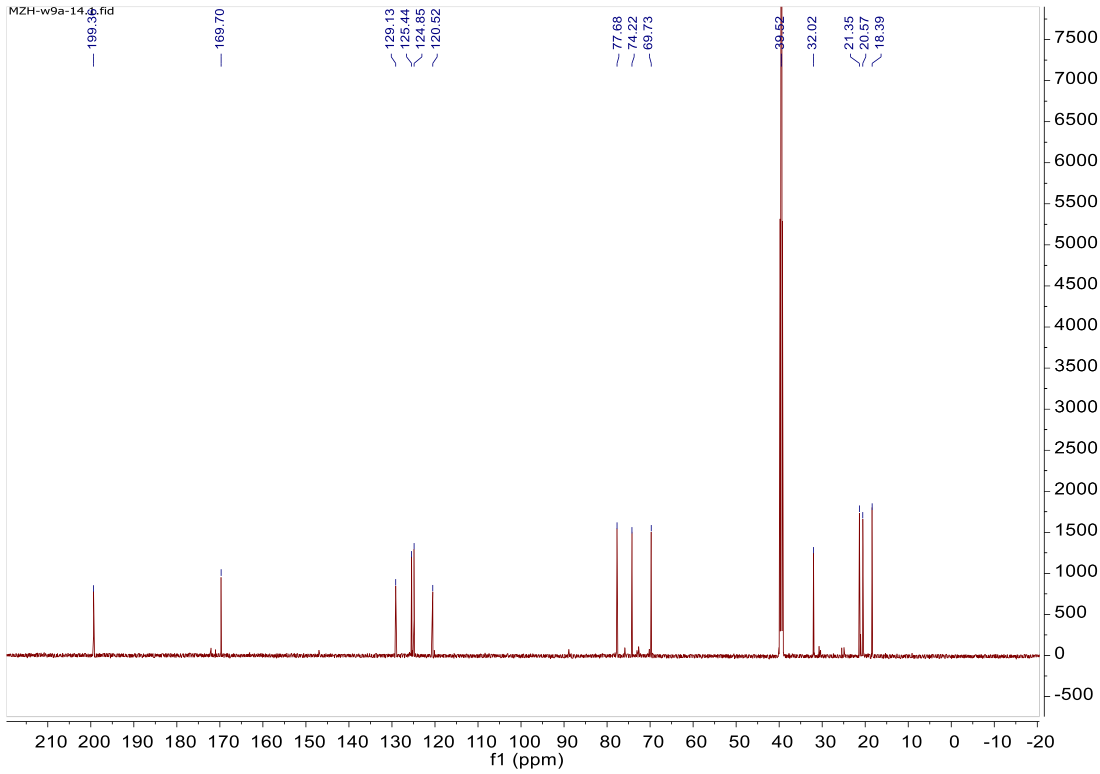

Figure S10. ^{13}C NMR spectrum of **2**

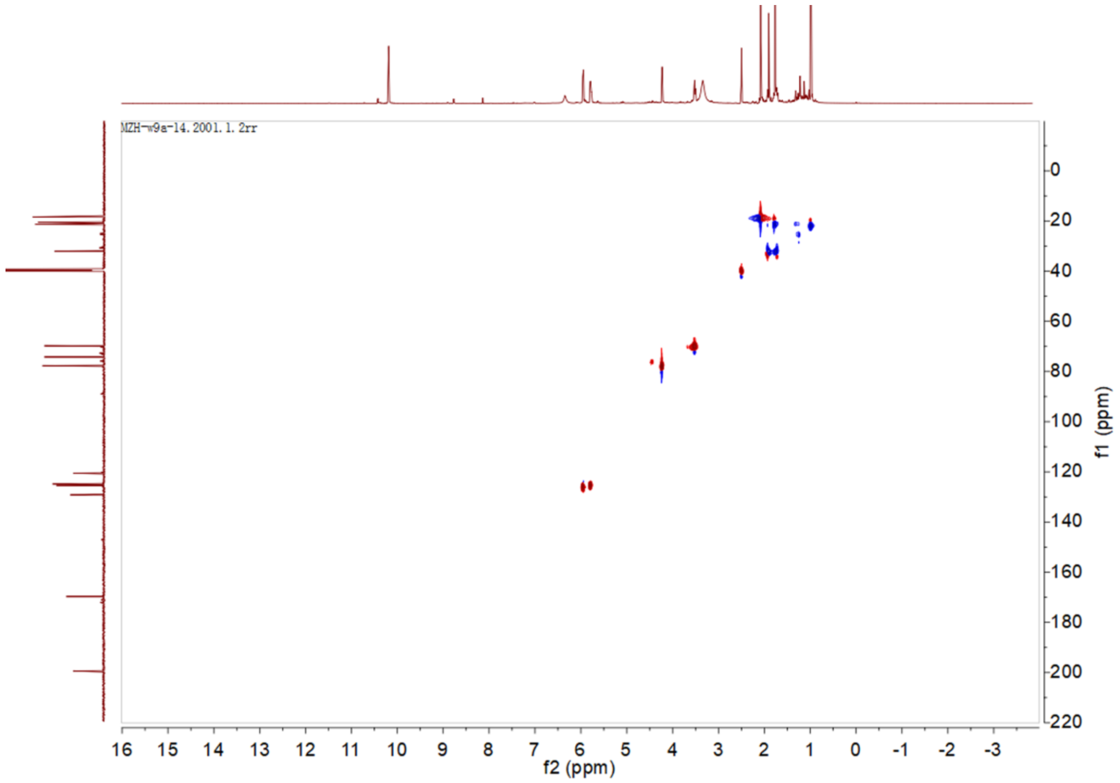

Figure S11. HSQC spectrum of **2**

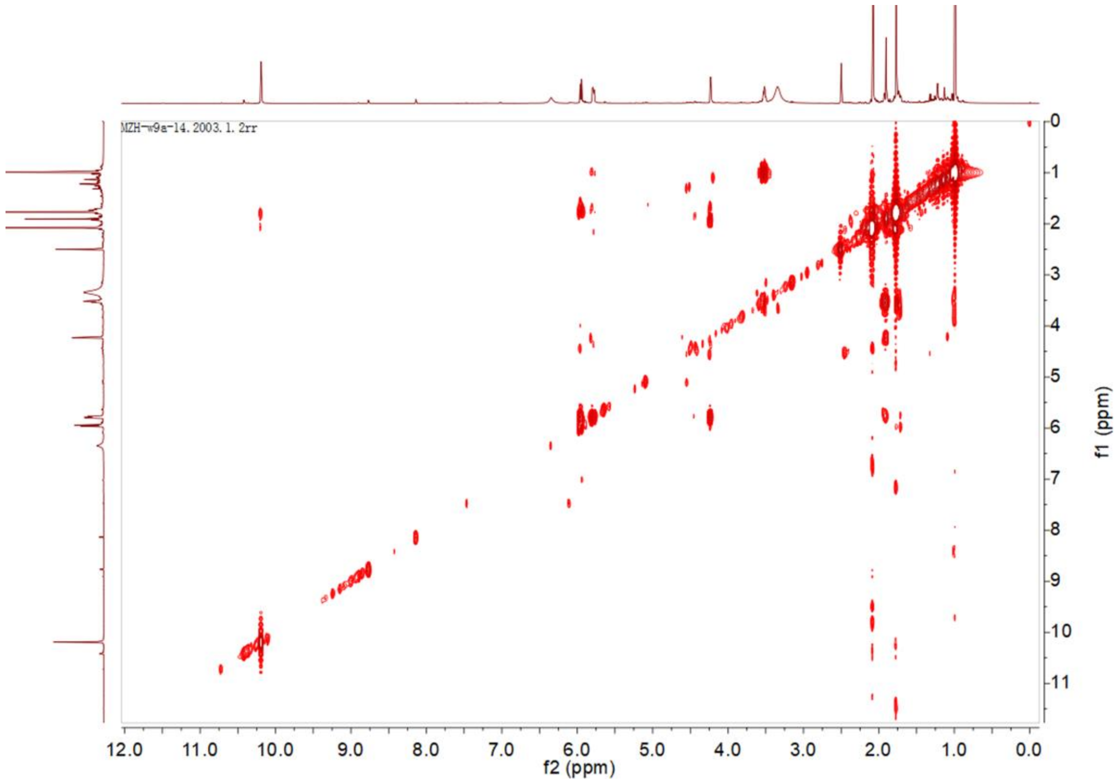

Figure S12. ^{1}H-^{1}H COSY spectrum of **2**

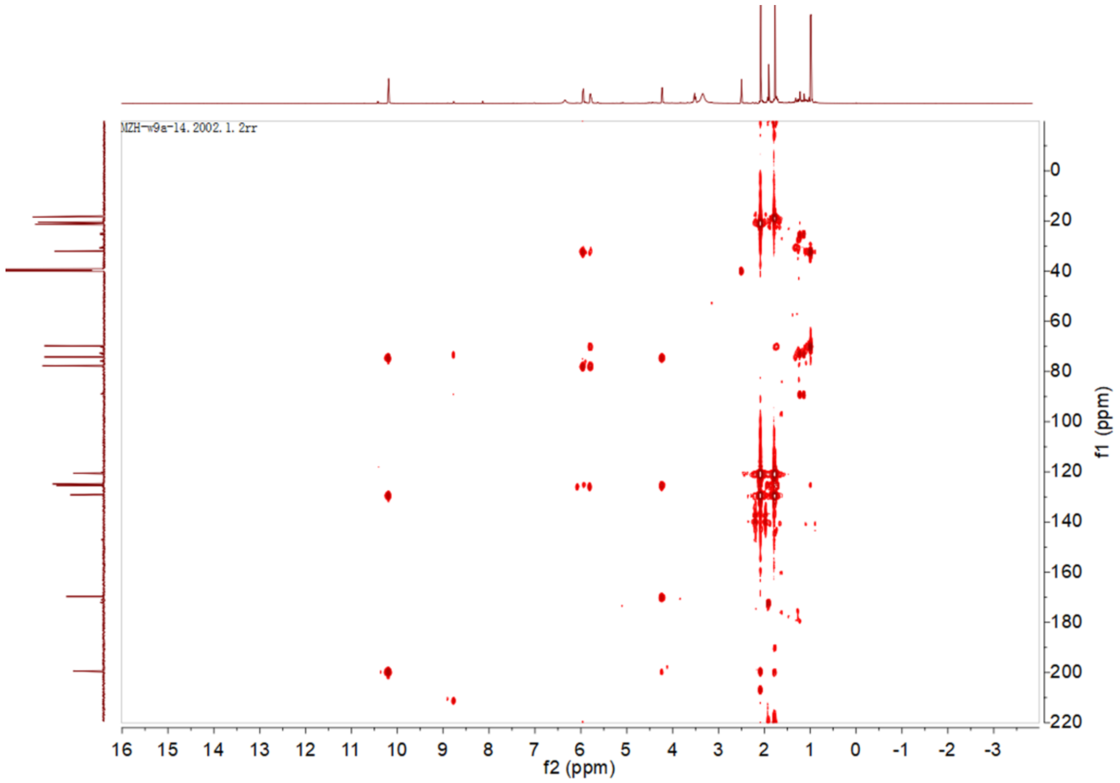

Figure S13. HMBC spectrum of 2

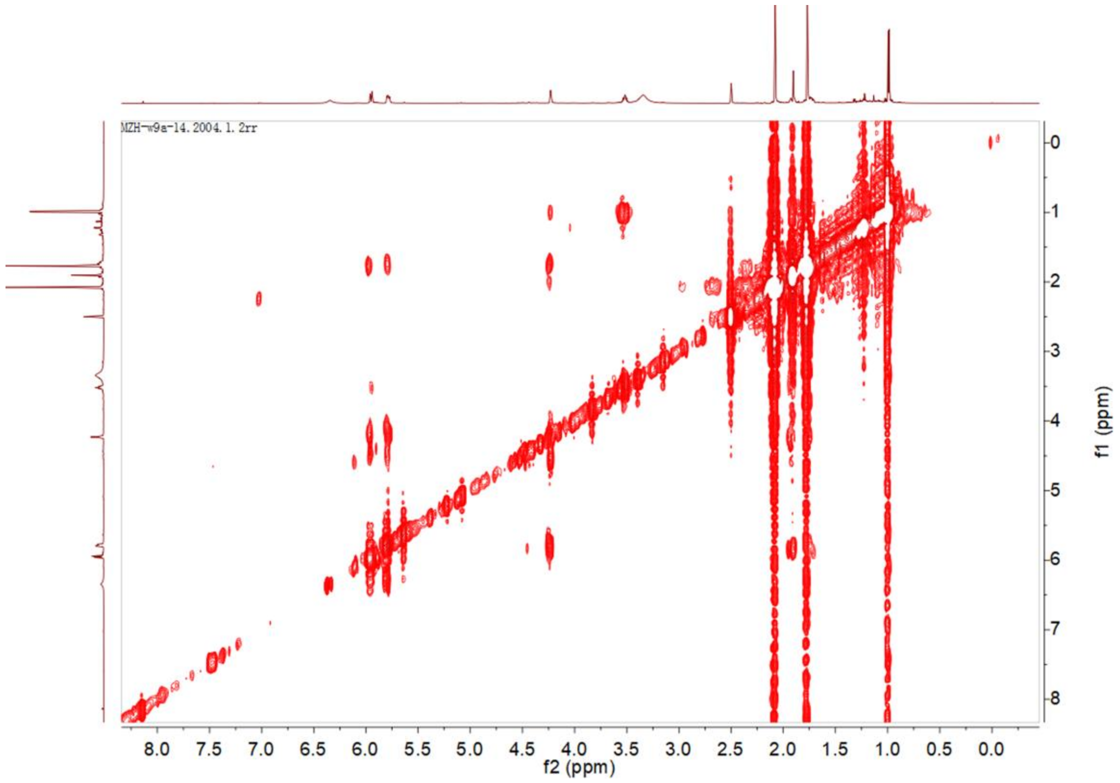

Figure S14. NOESY spectrum of 2

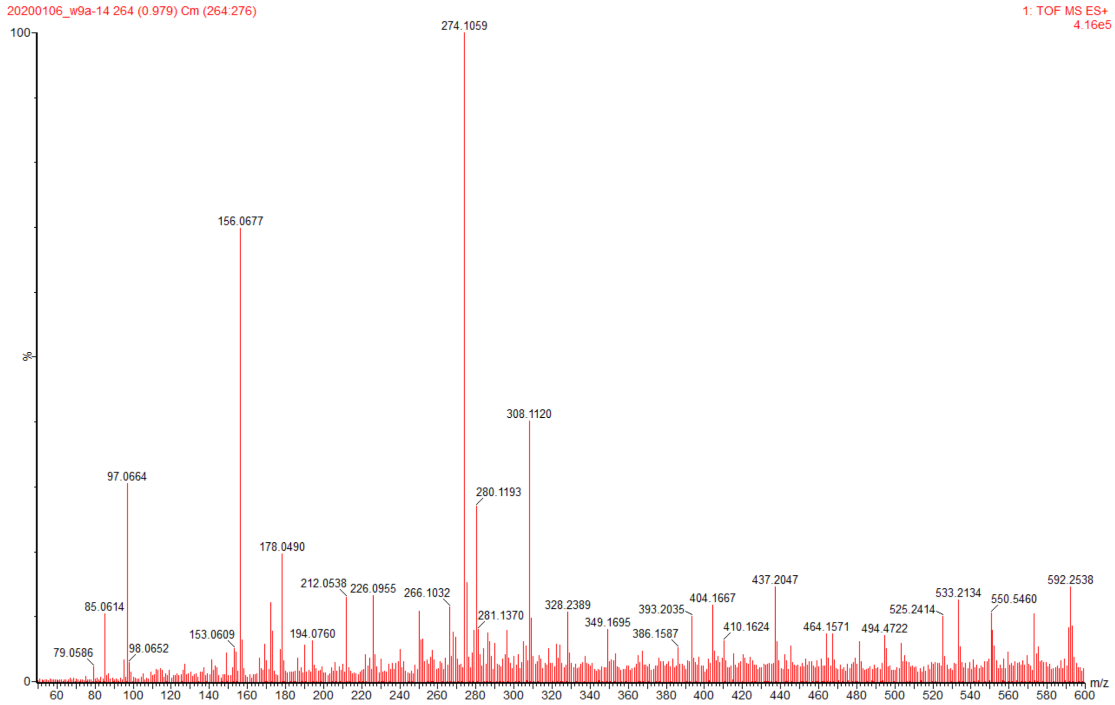

Figure S15. HR-ESI-MS spectrum of **2**

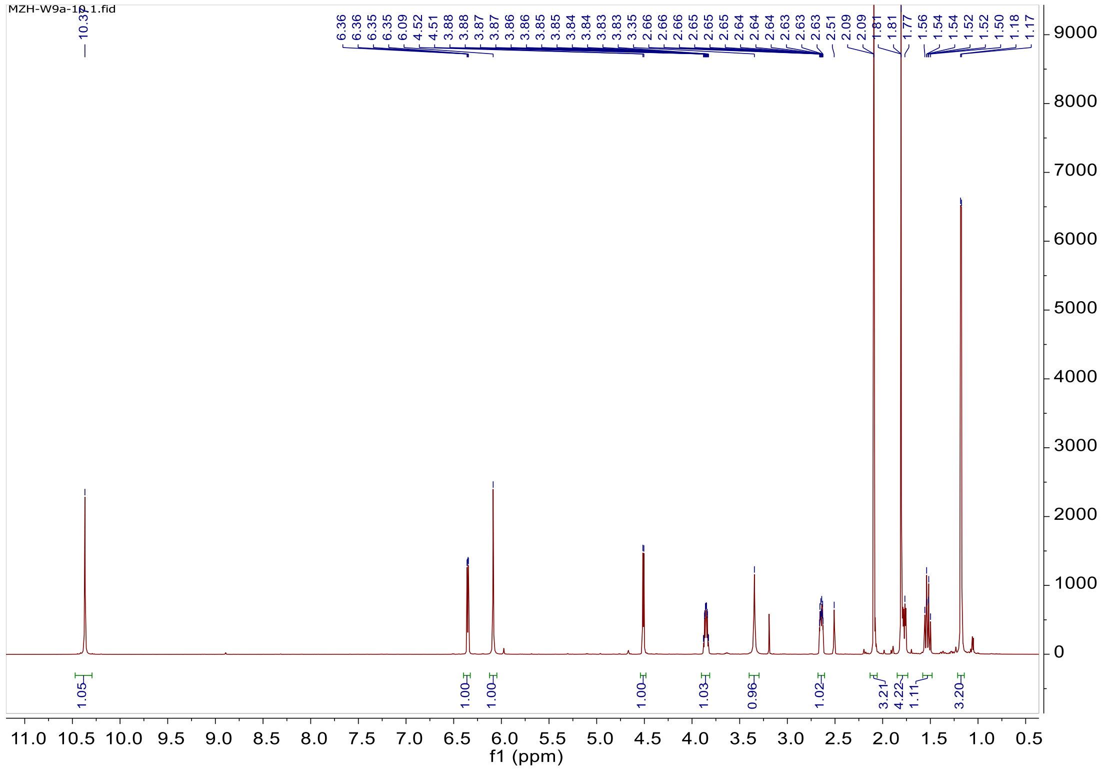

Figure S16. ^{1}H NMR spectrum of **3**

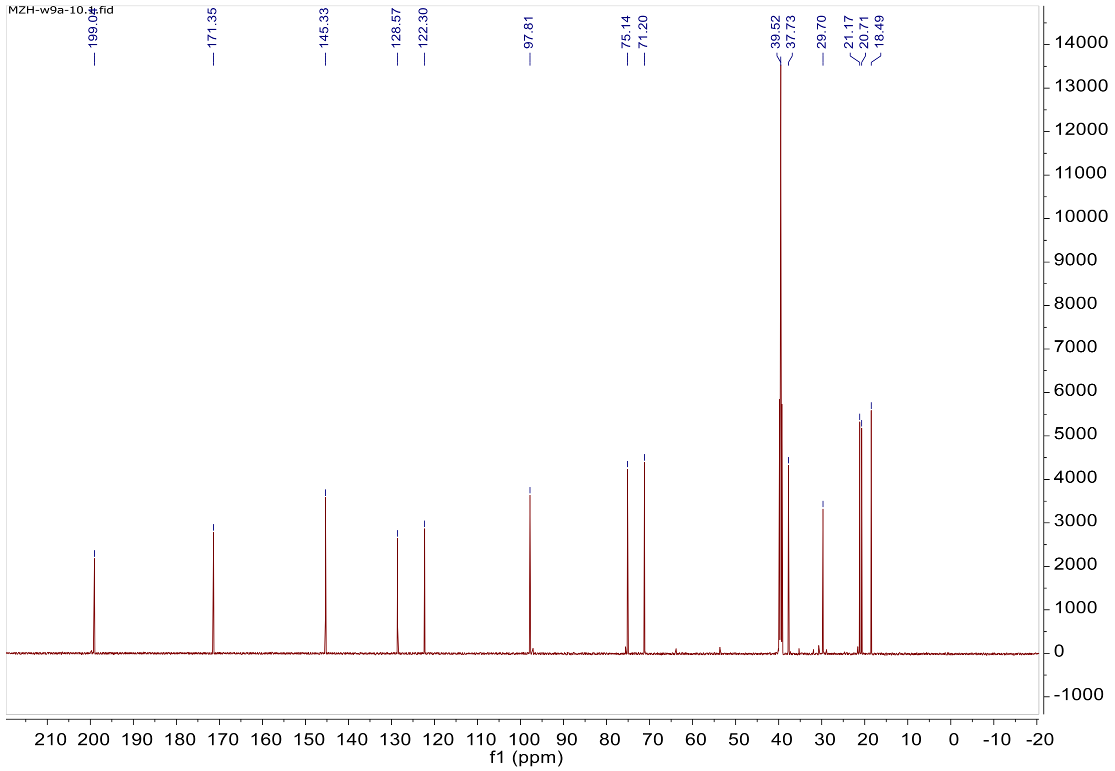

Figure S17. ^{13}C NMR spectrum of **3**

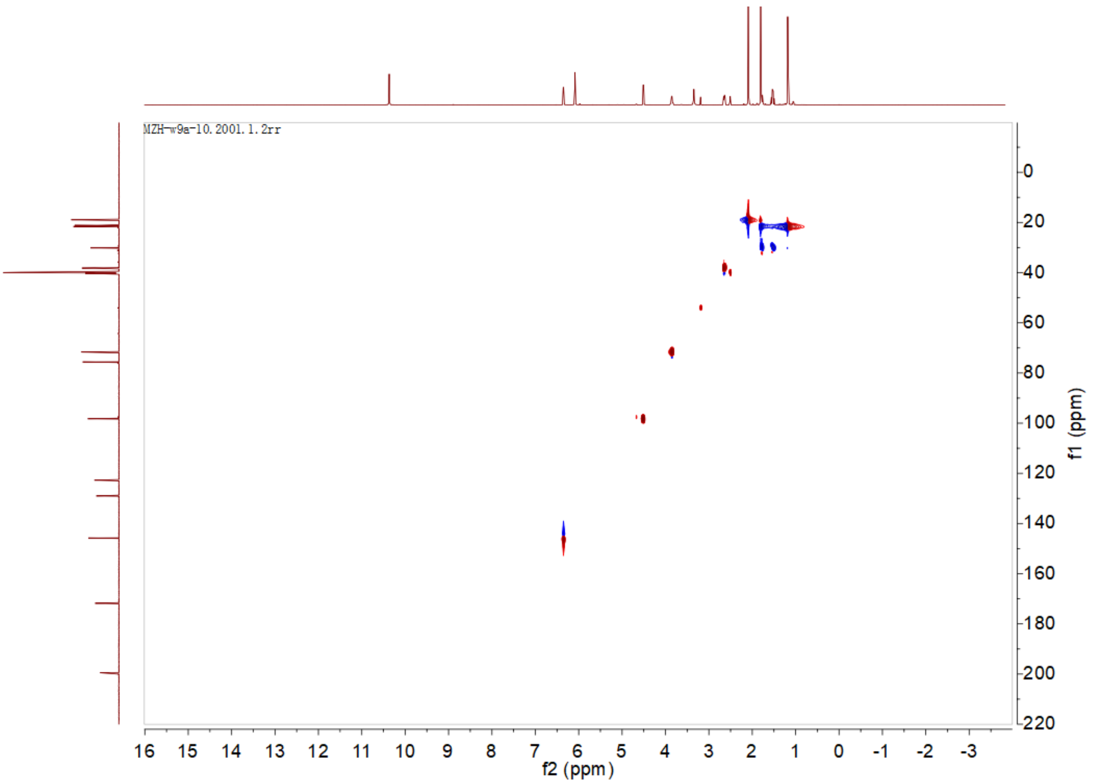

Figure S18. HSQC spectrum of **3**

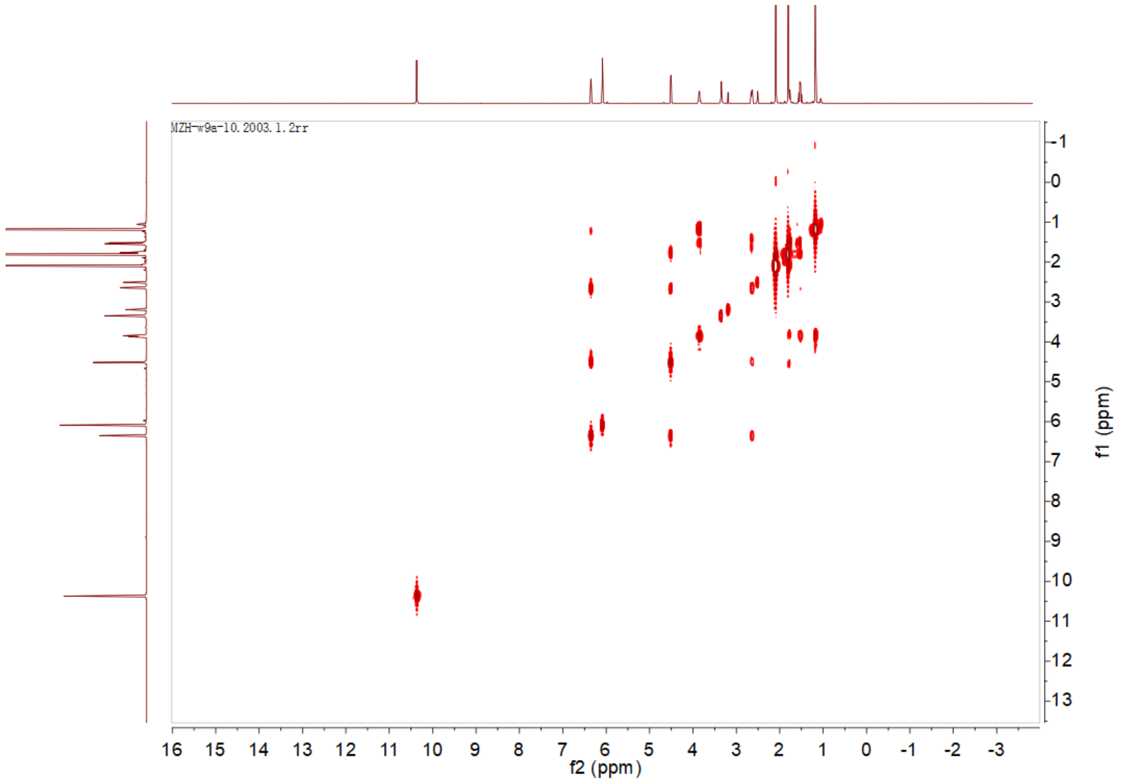

Figure S19. ^{1}H-^{1}H COSY spectrum of **3**

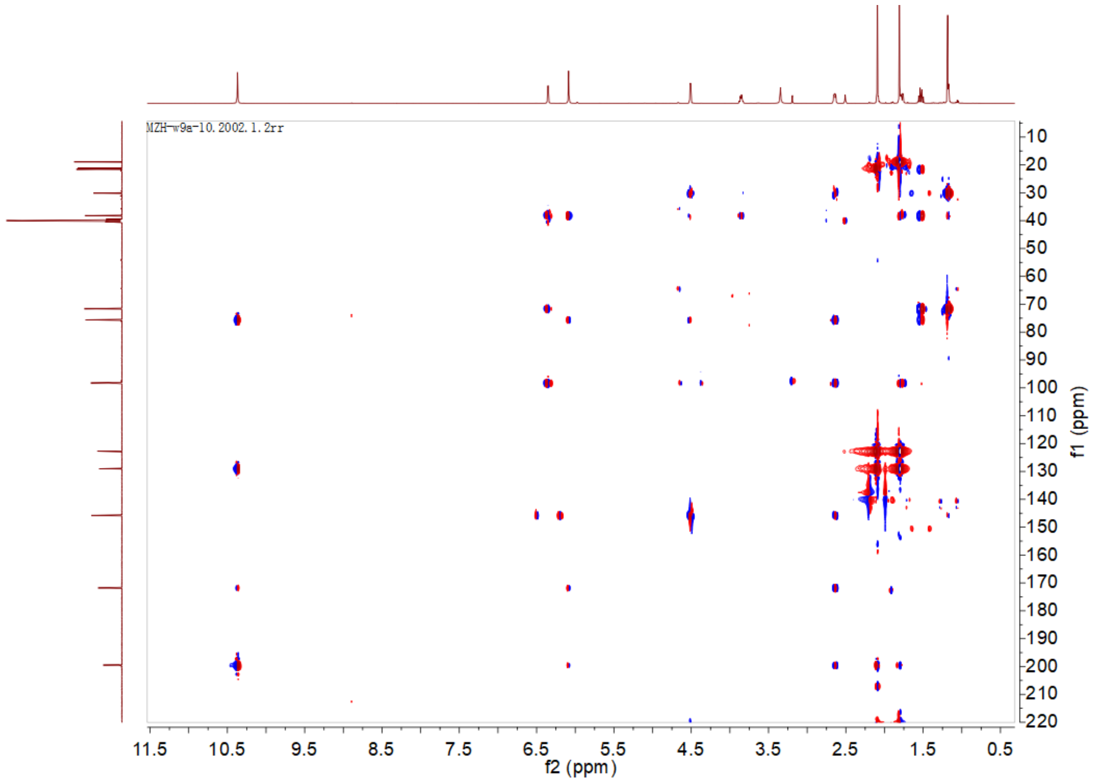

Figure S20. HMBC spectrum of **3**

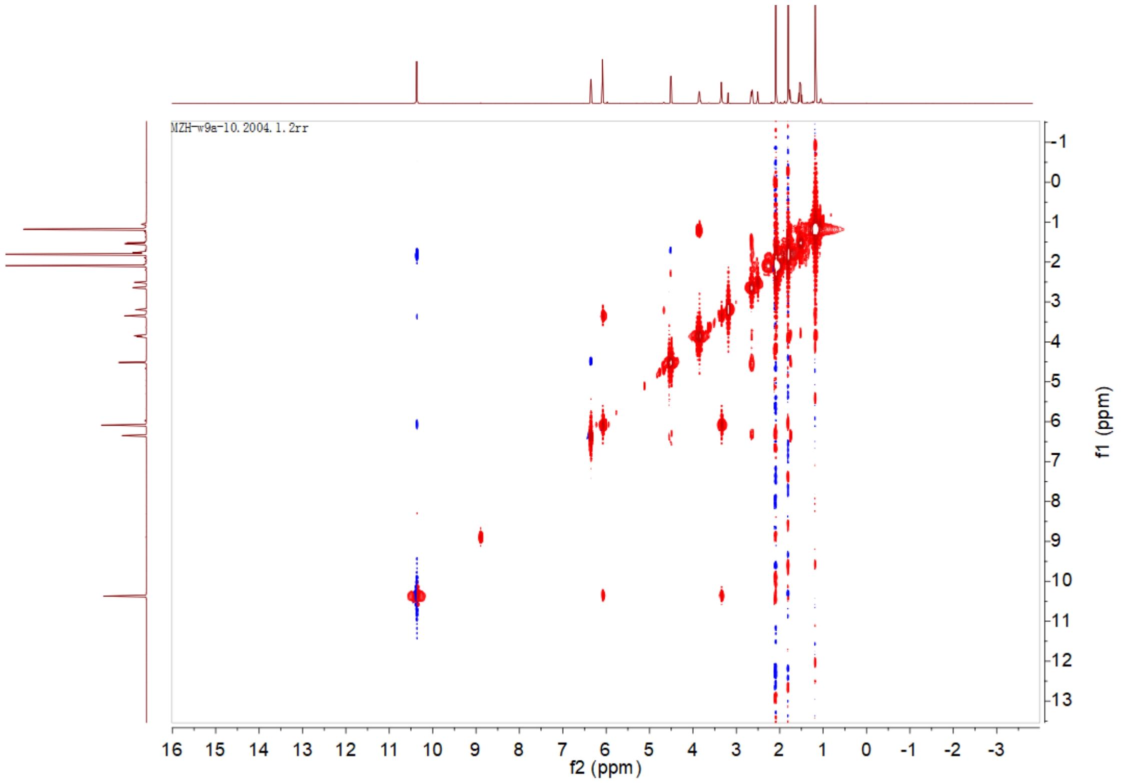

Figure S21. NOESY spectrum of **3**

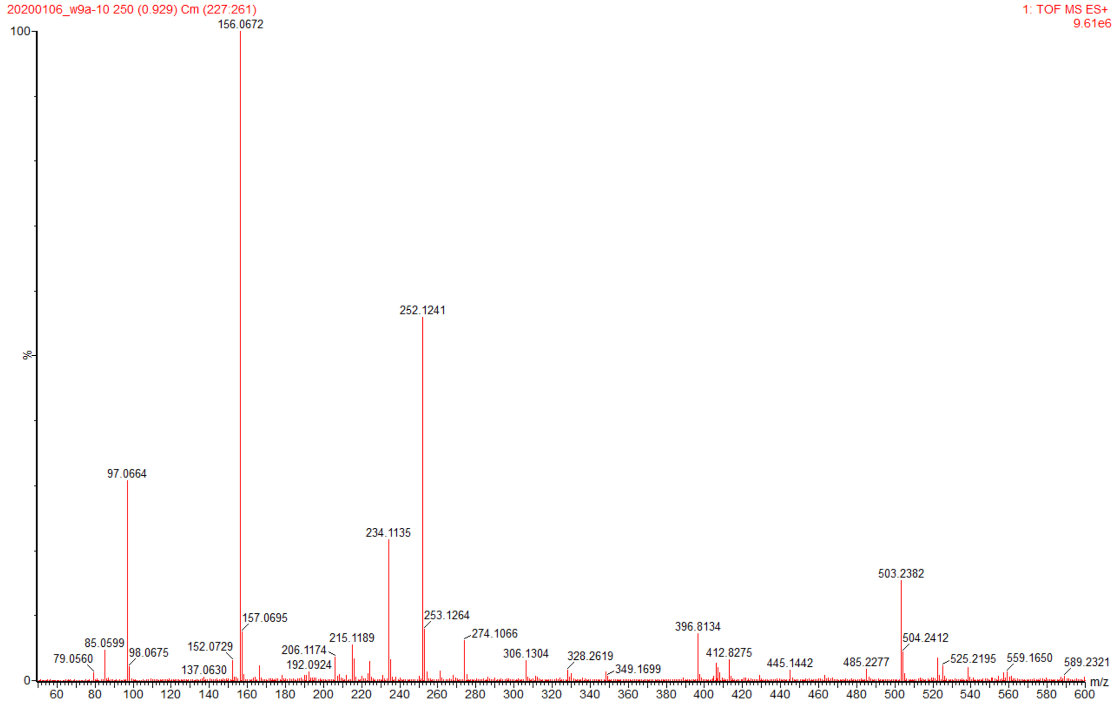

Figure S22. HR-ESI-MS spectrum of **3**

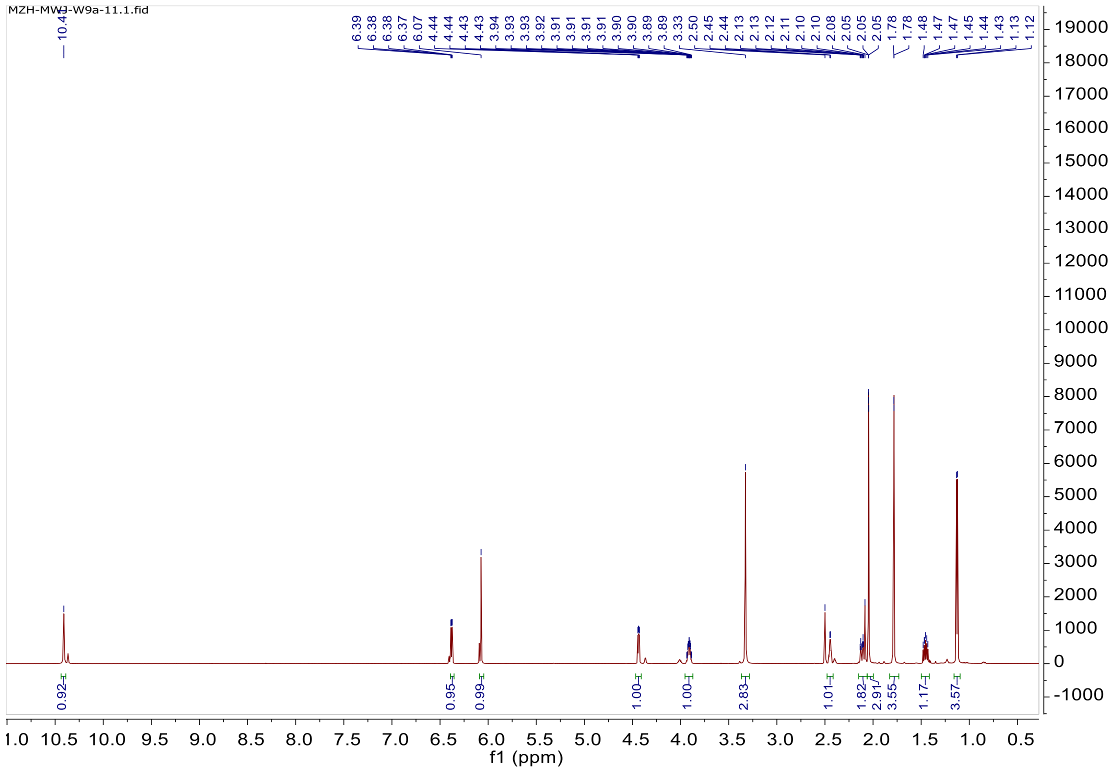

Figure S23. ^{1}H NMR spectrum of **4**

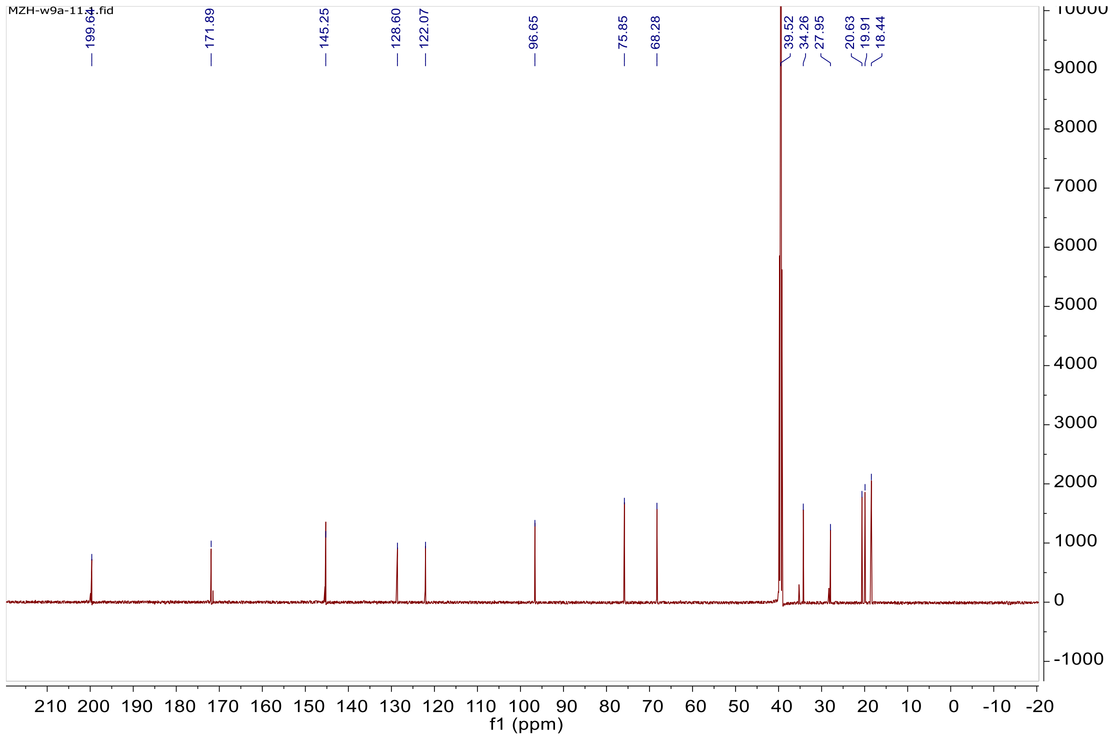

Figure S24. ^{13}C NMR spectrum of **4**

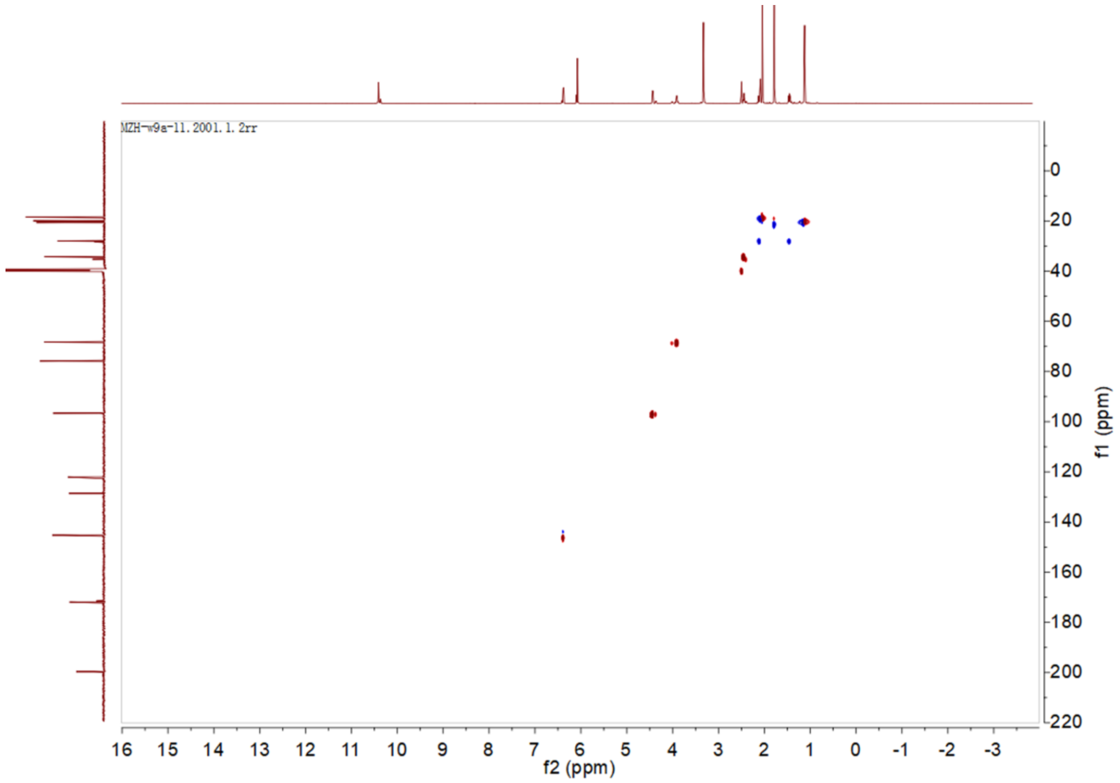

Figure S25. HSQC spectrum of **4**

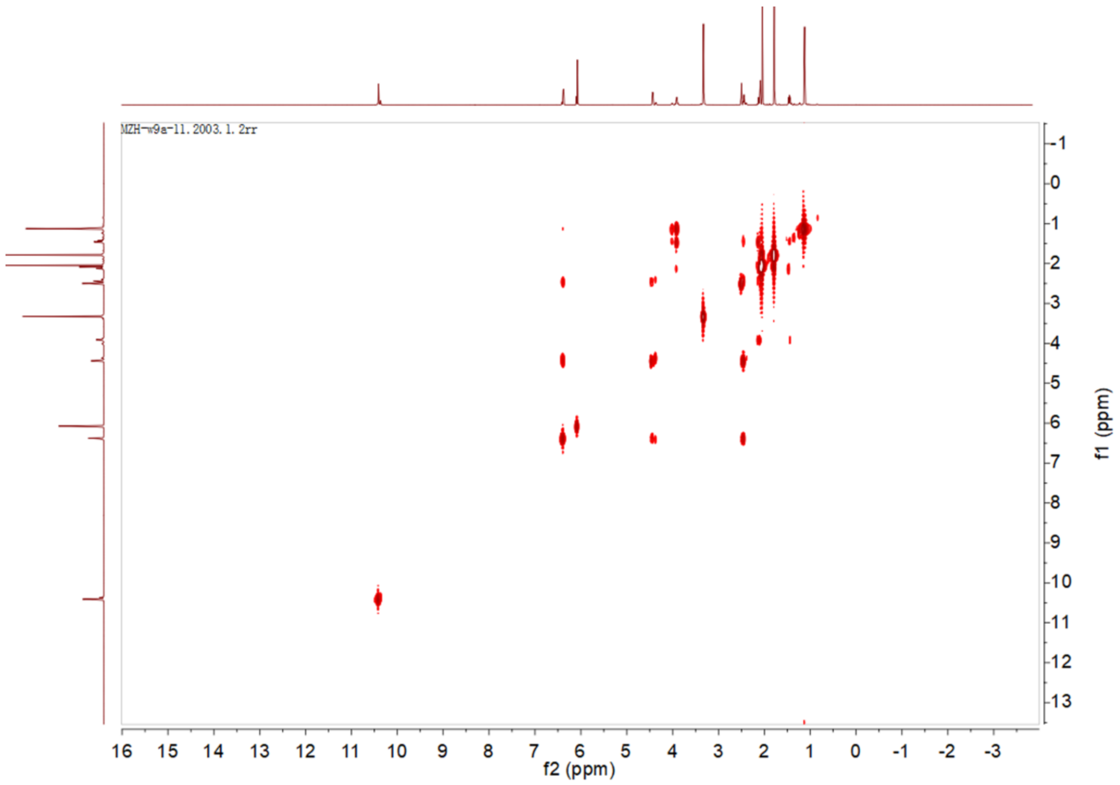

Figure S26. ^{1}H-^{1}H COSY spectrum of **4**

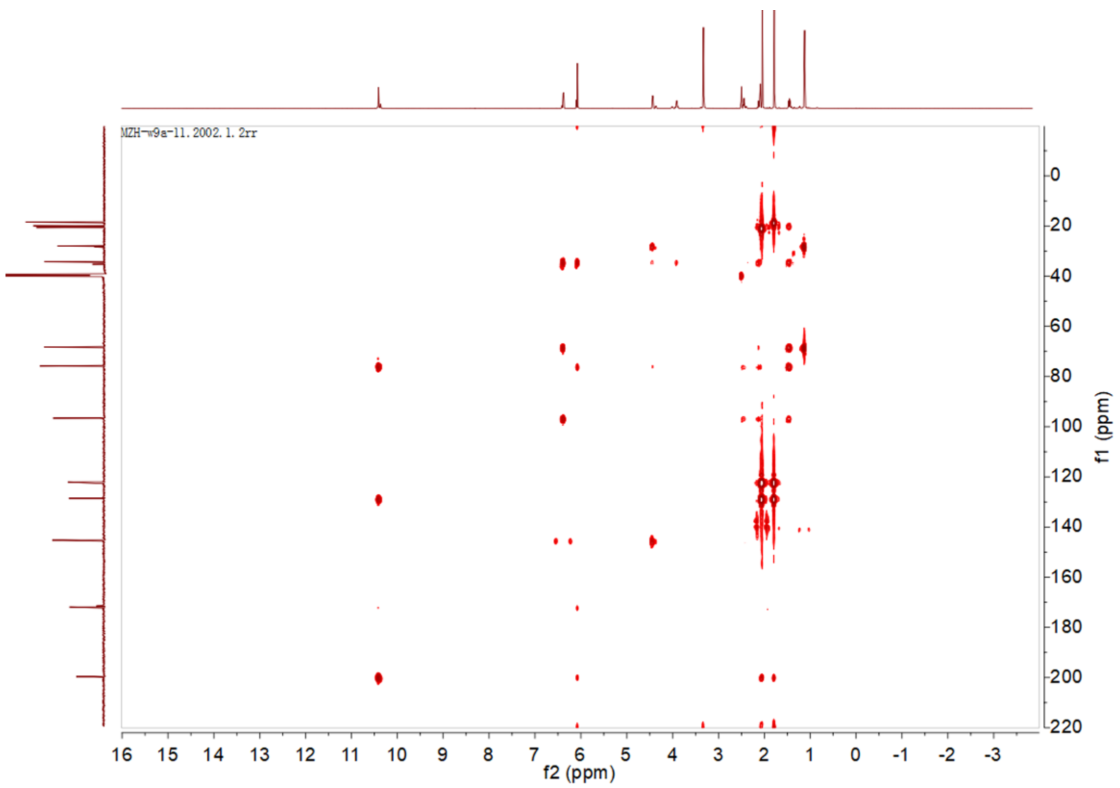

Figure S27. HMBC spectrum of **4**

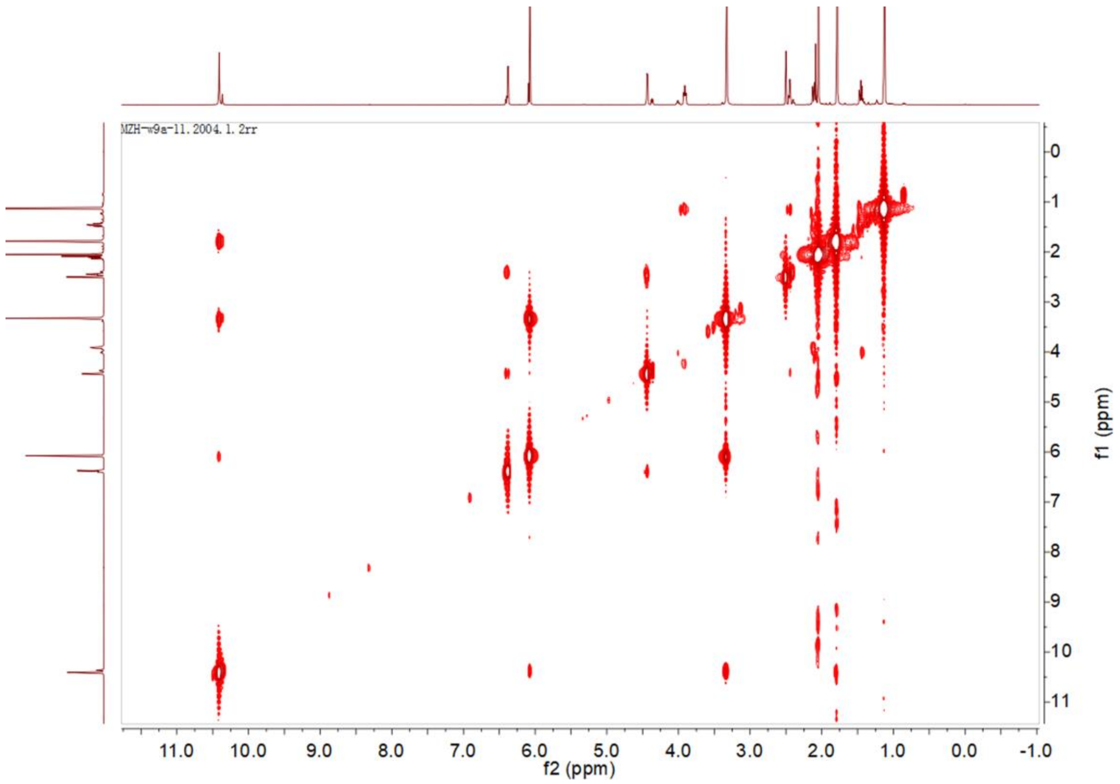

Figure S28. NOESY spectrum of **4**

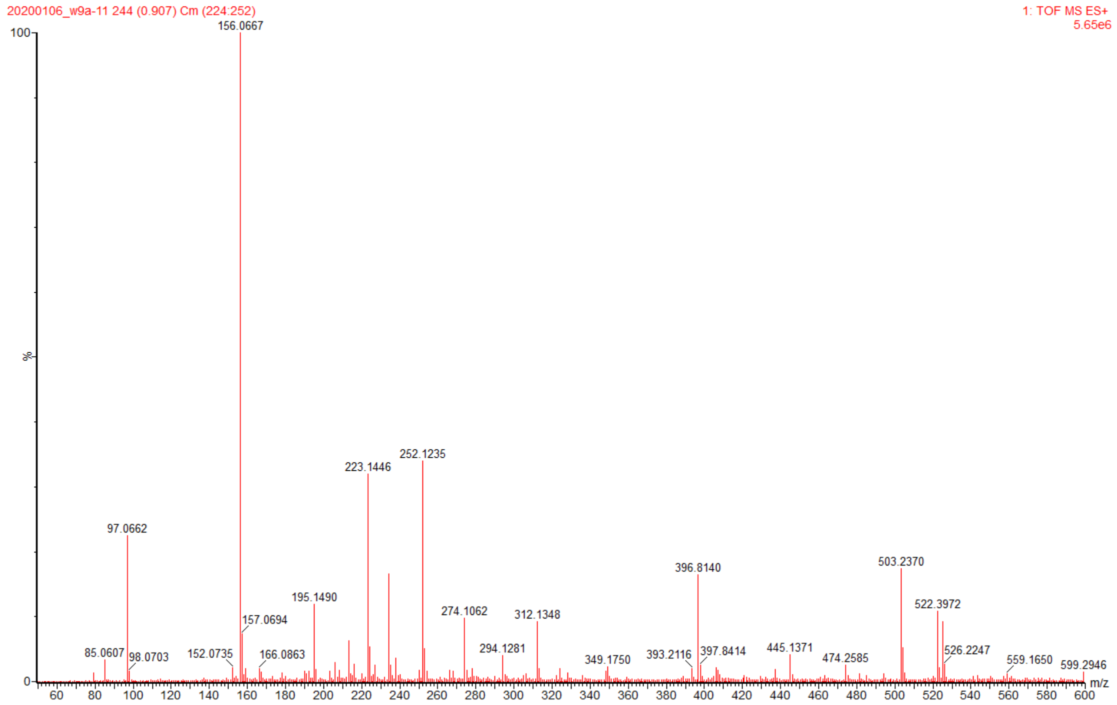

Figure S29. HR-ESI-MS spectrum of **4**

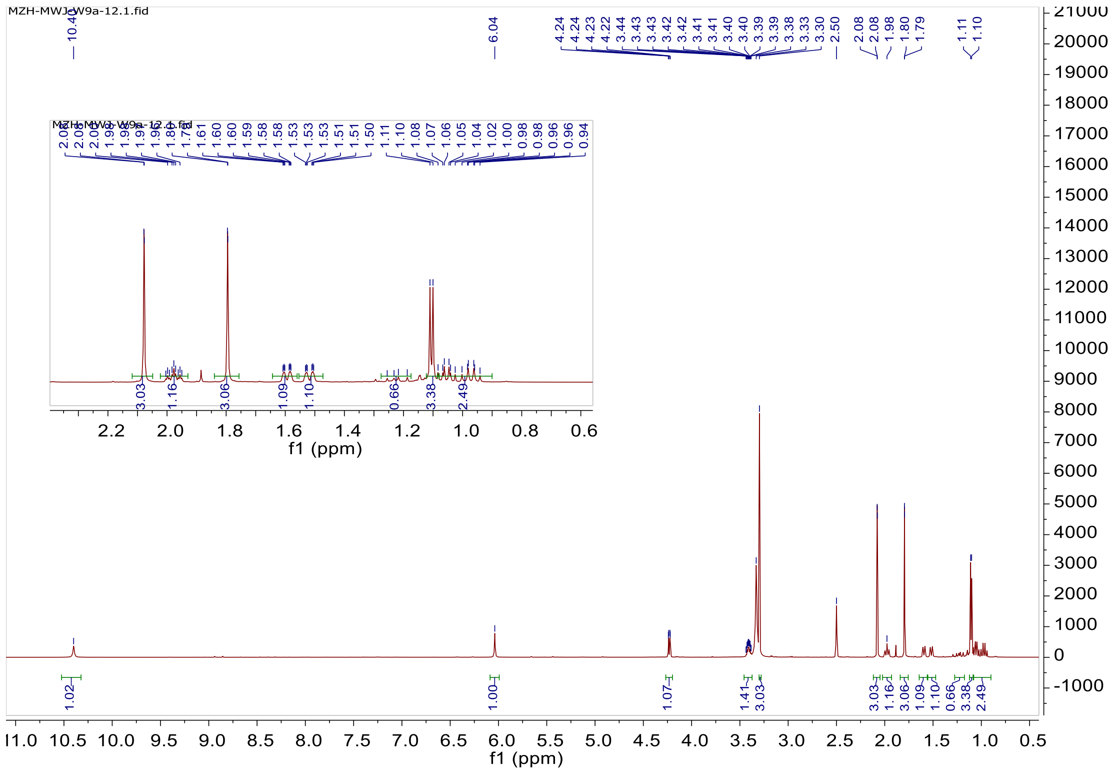

Figure S30. ^{1}H NMR spectrum of **5**

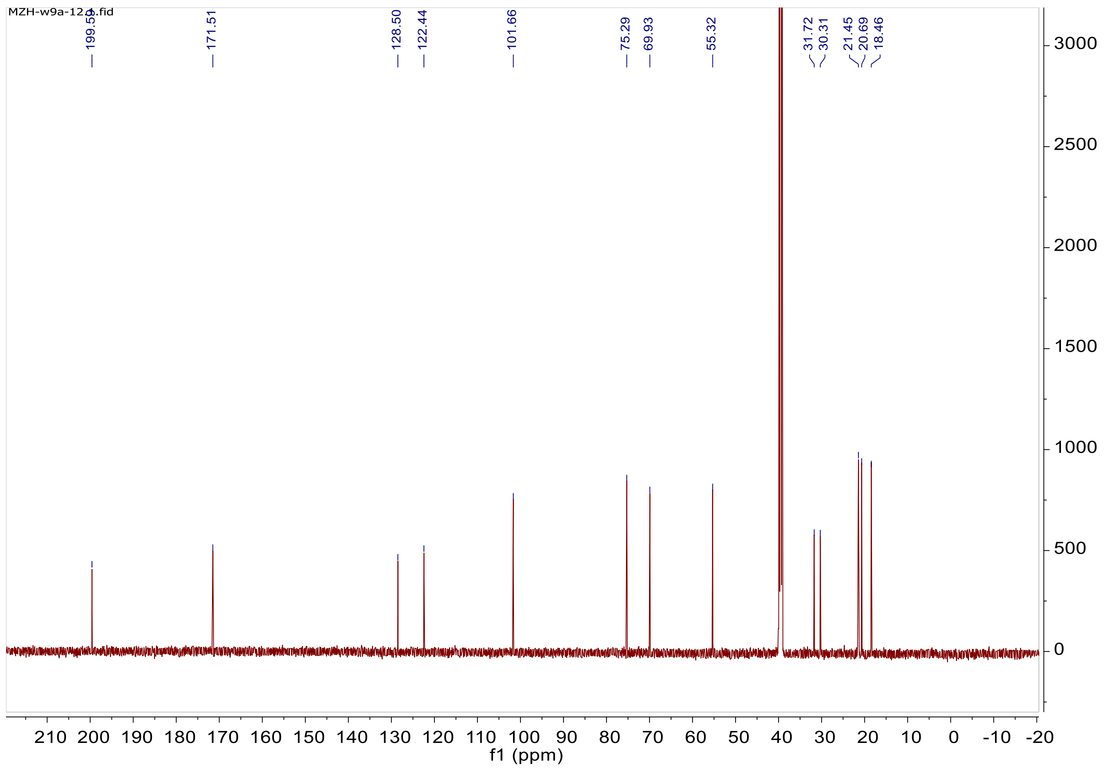

Figure S31. ^{13}C NMR spectrum of **5**

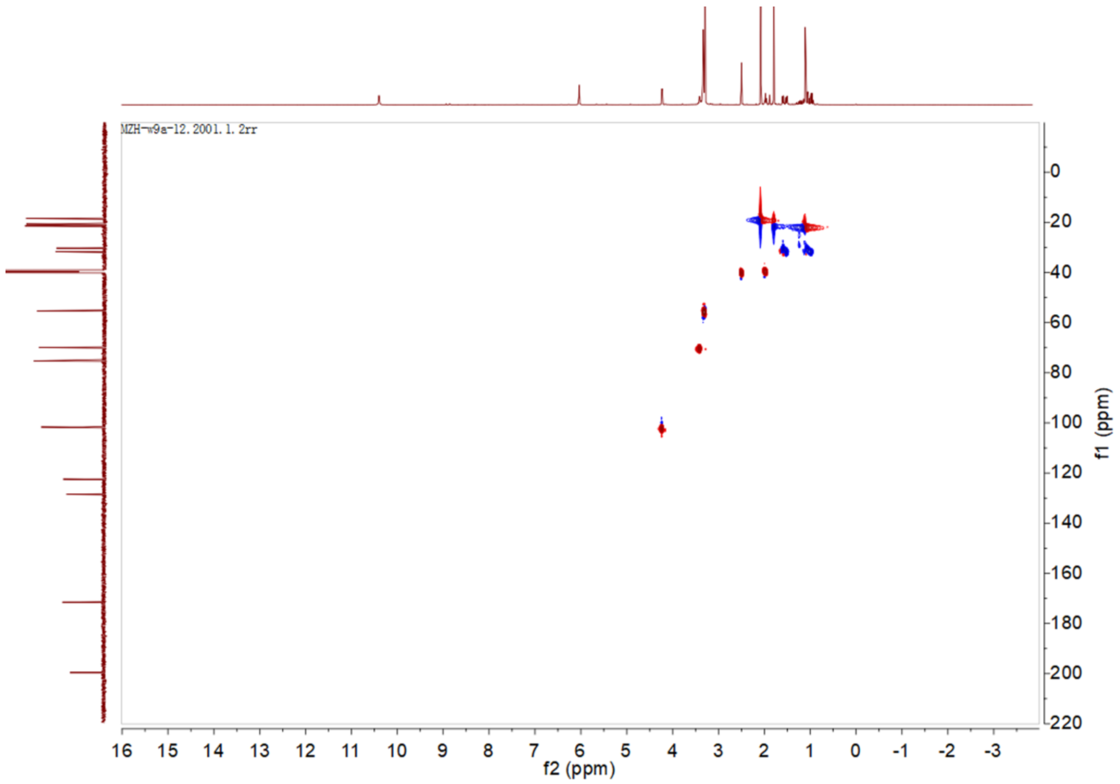

Figure S32. HSQC spectrum of **5**

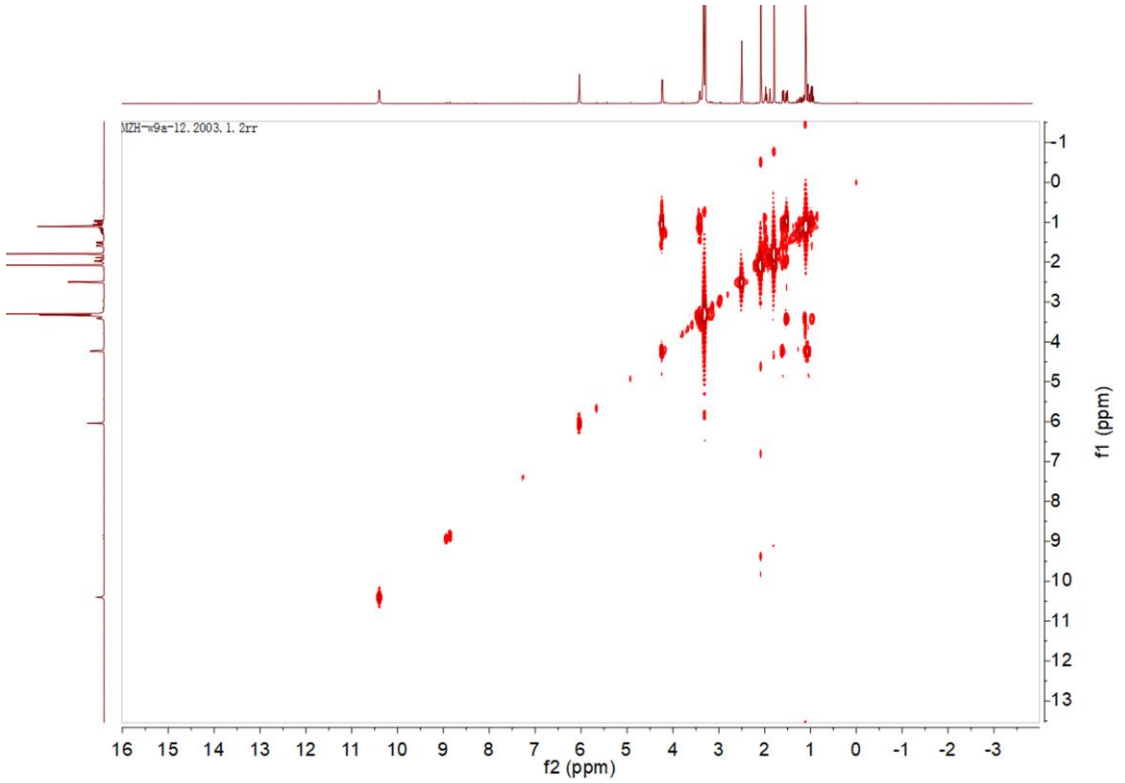

Figure S33. ^{1}H-^{1}H COSY spectrum of **5**

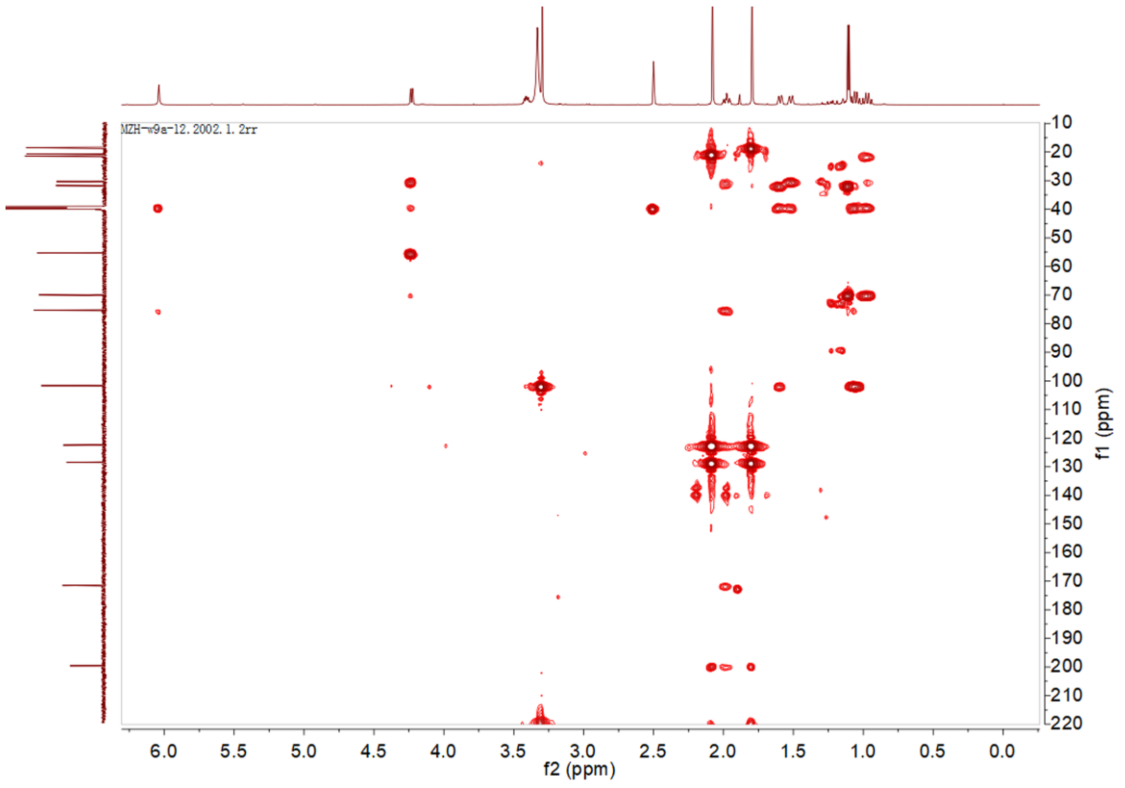

Figure S34. HMBC spectrum of **5**

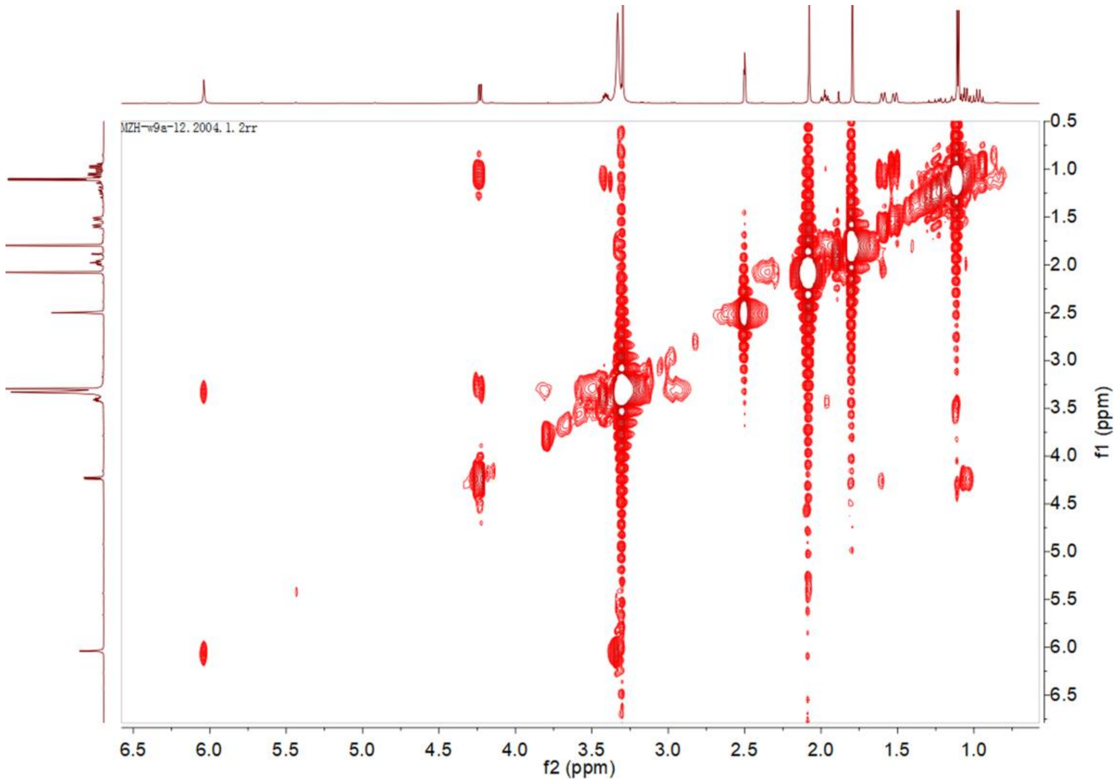

Figure S35. NOESY spectrum of **5**

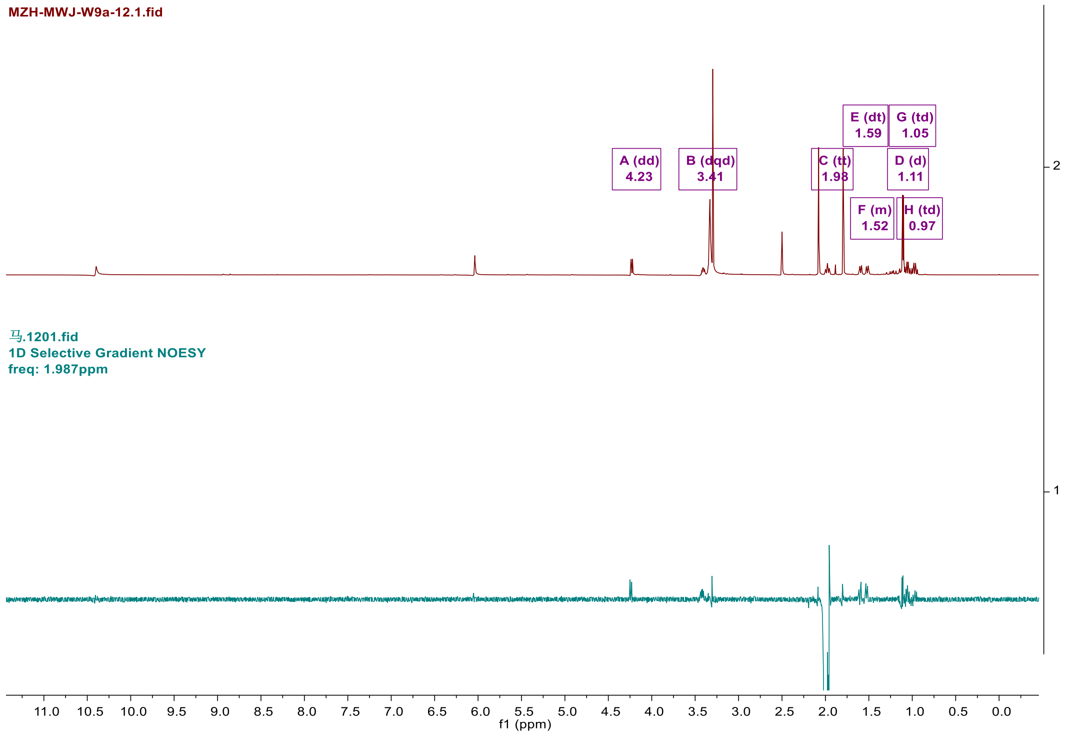

Figure S36. 1D selective NOESY spectrum of **5** (1.98ppm)

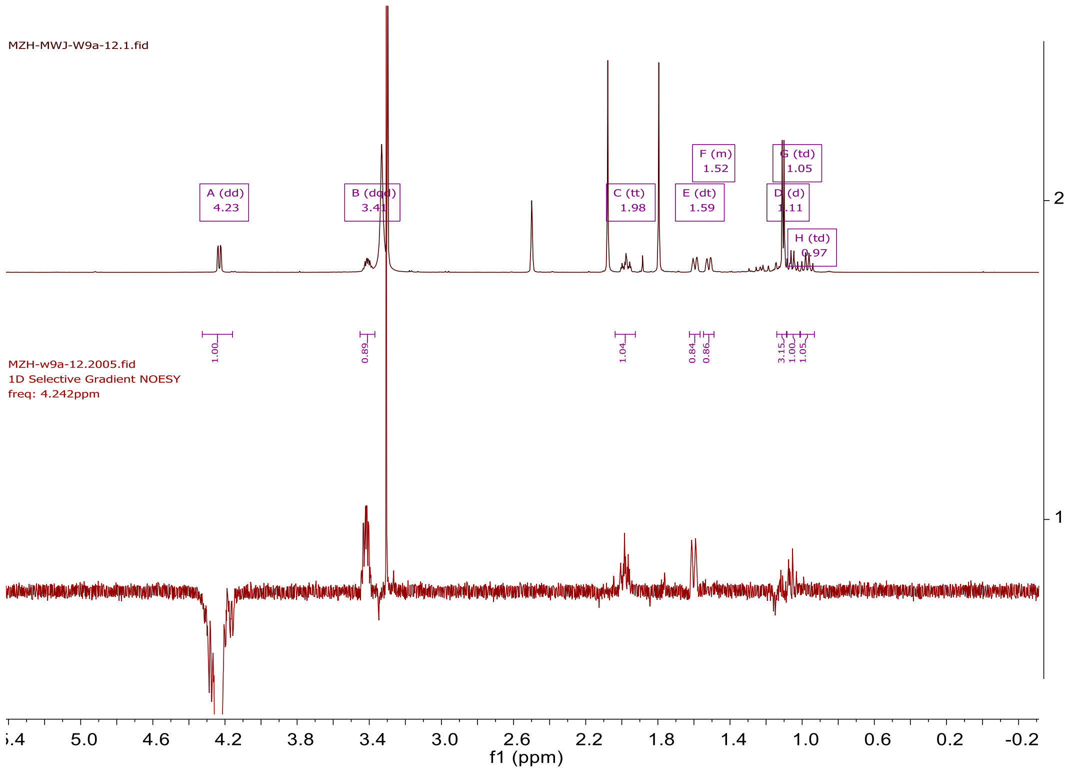

Figure S37. 1D selective NOESY spectrum of **5** (4.24ppm)

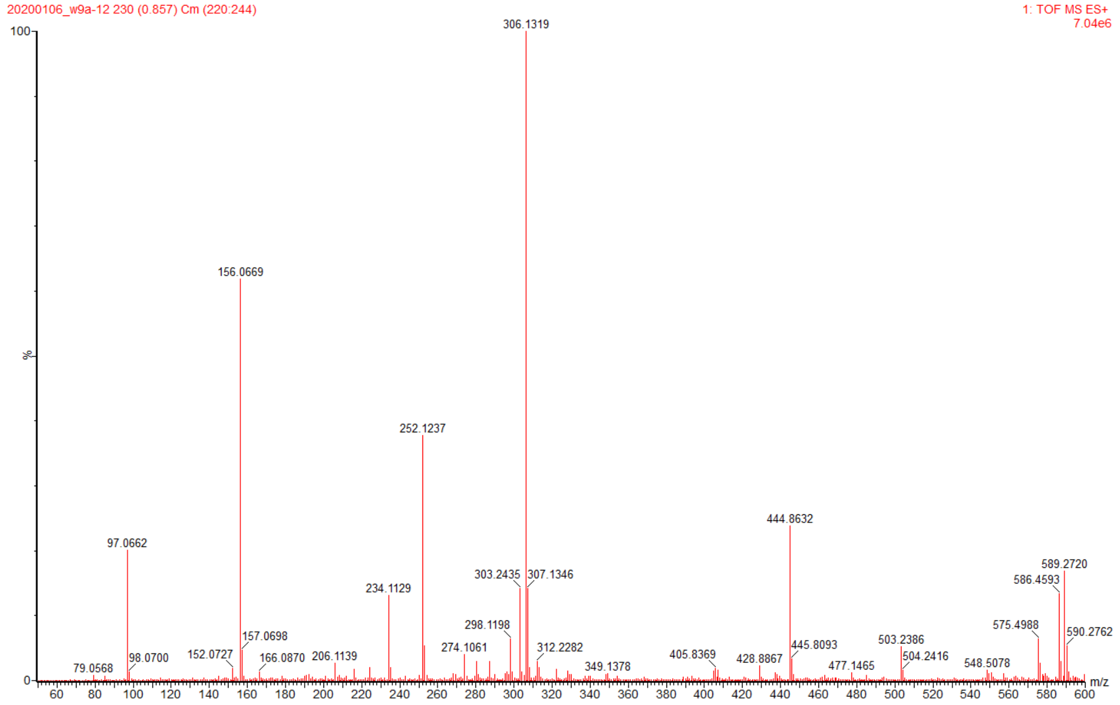

Figure S38. HR-ESI-MS spectrum of **5**

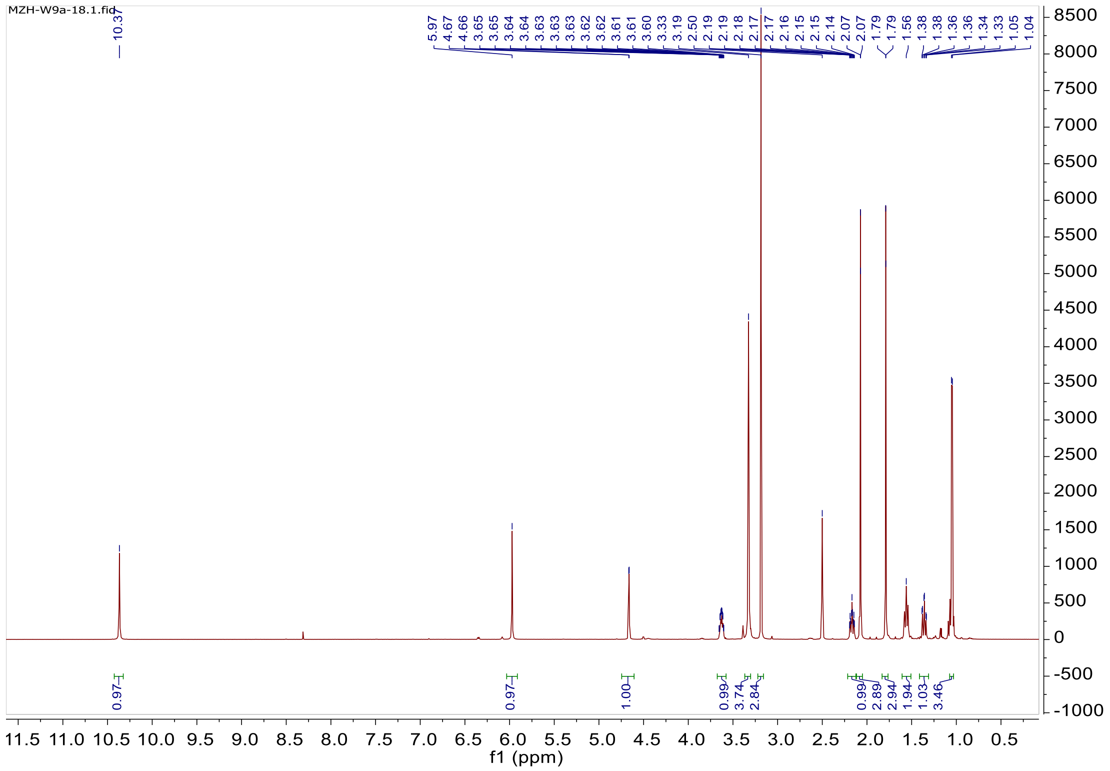

Figure S39. ^{1}H NMR spectrum of **6**

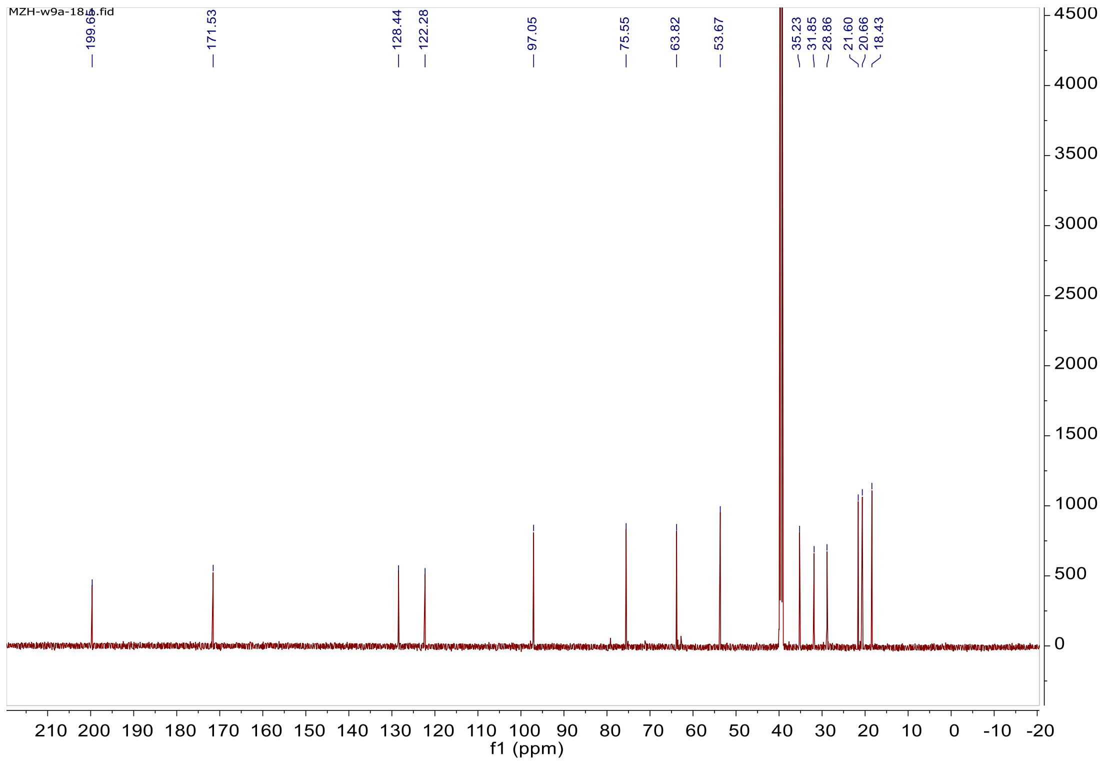

Figure S40. ^{13}C NMR spectrum of **6**

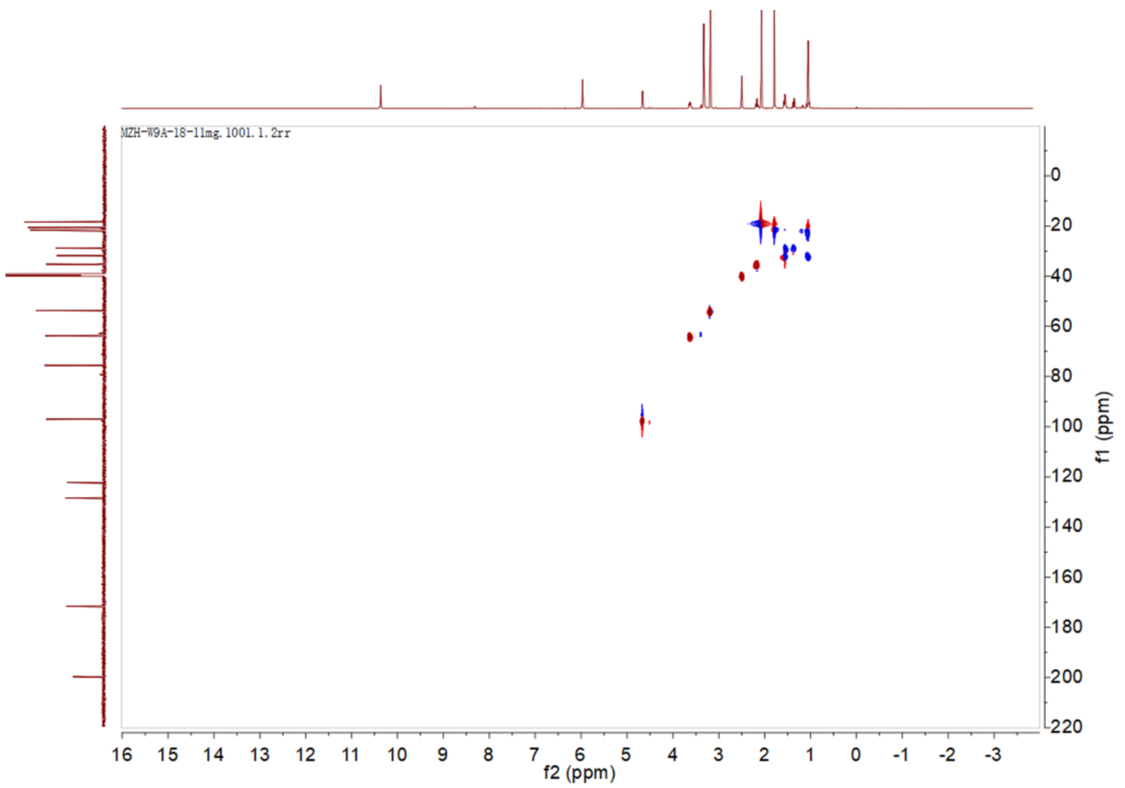

Figure S41. HSQC spectrum of **6**

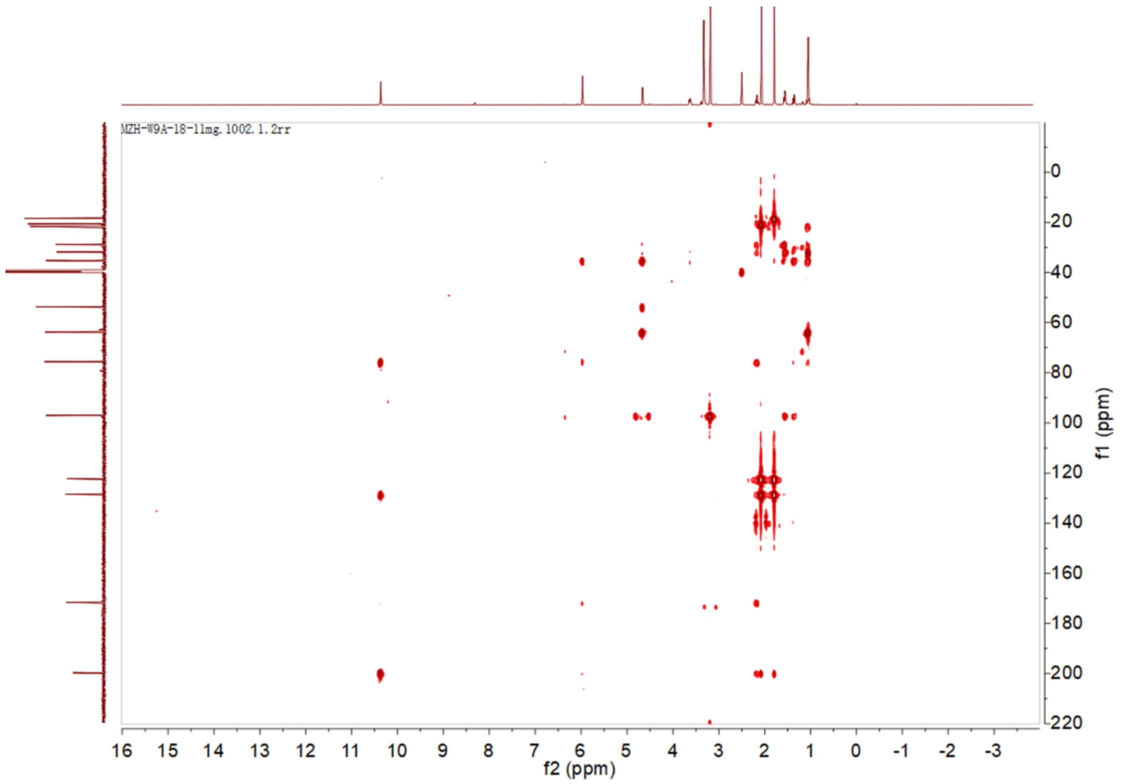

Figure S42. ^{1}H-^{1}H COSY spectrum of **6**

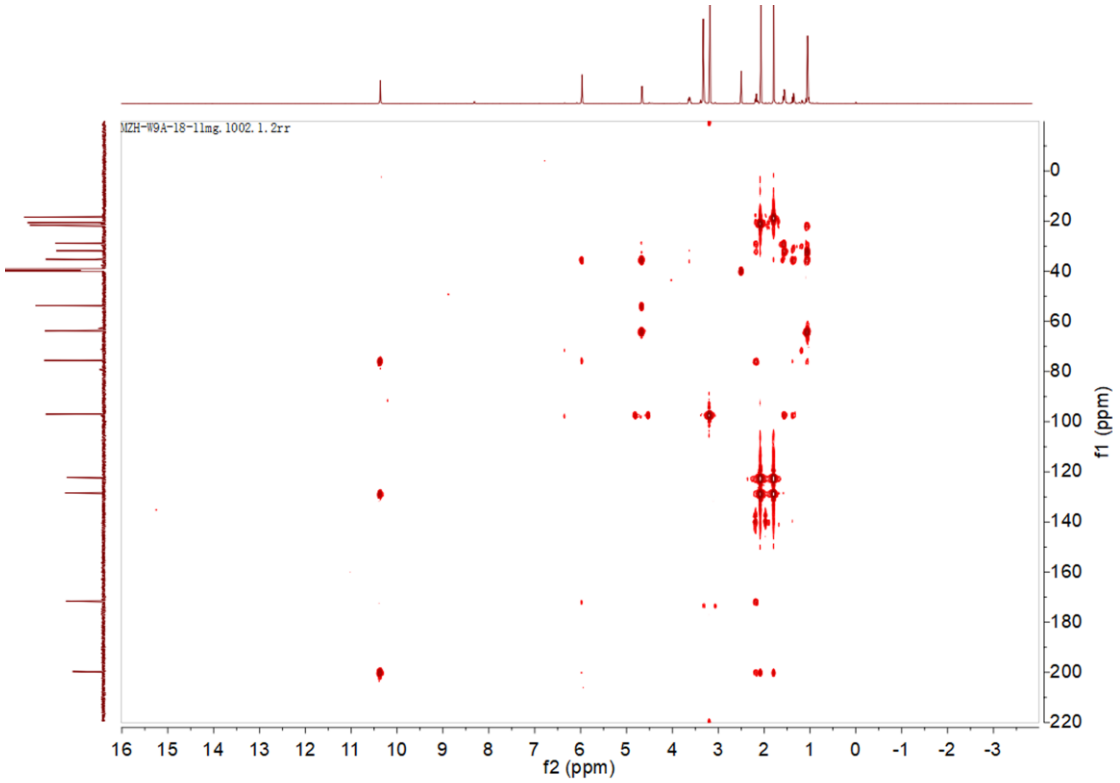

Figure S43. HMBC spectrum of **6**

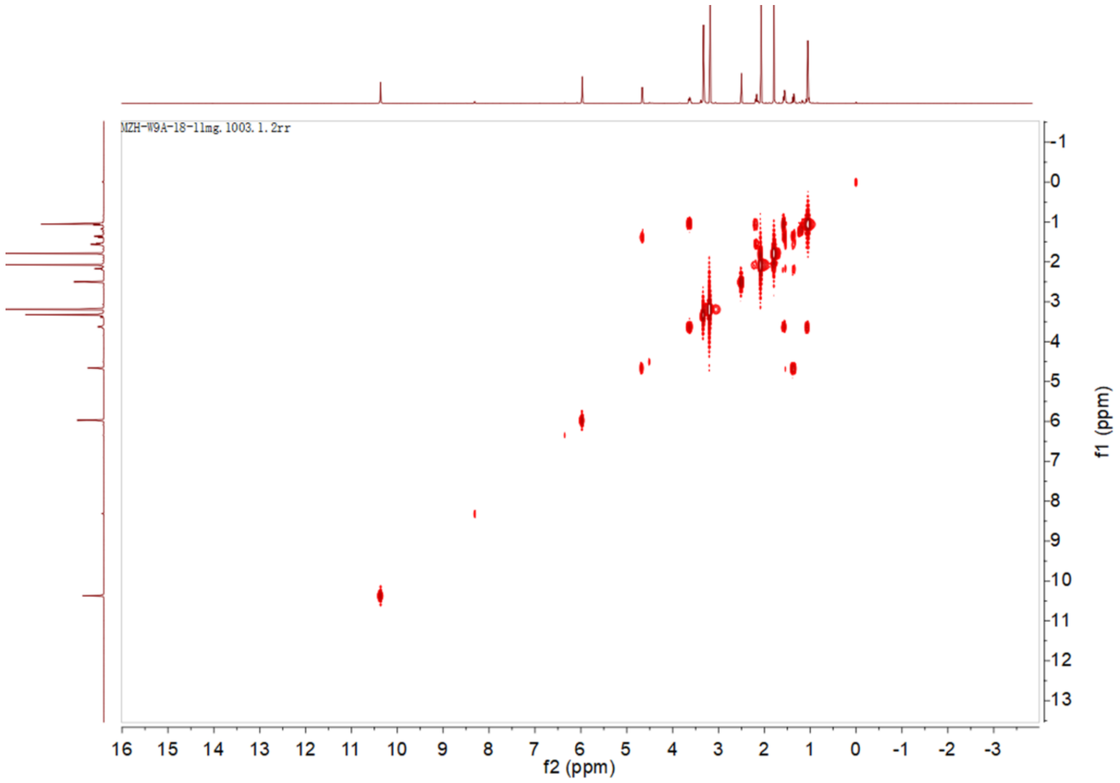

Figure S44. NOESY spectrum of **6**

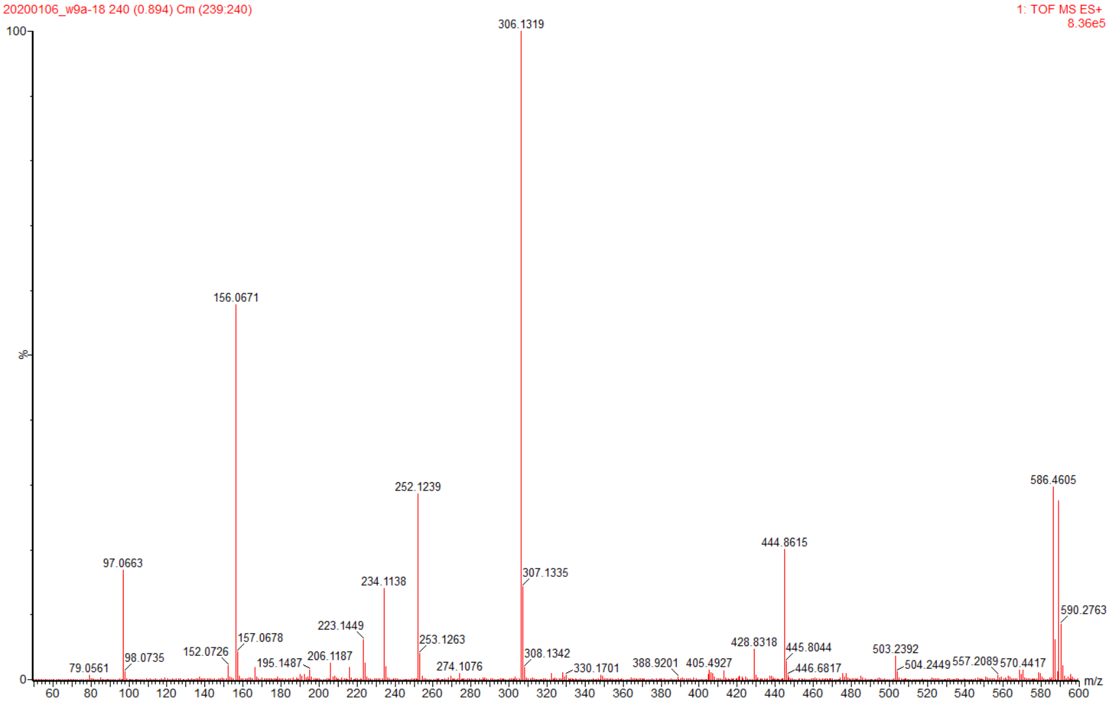

Figure S45. HR-ESI-MS spectrum of **6**

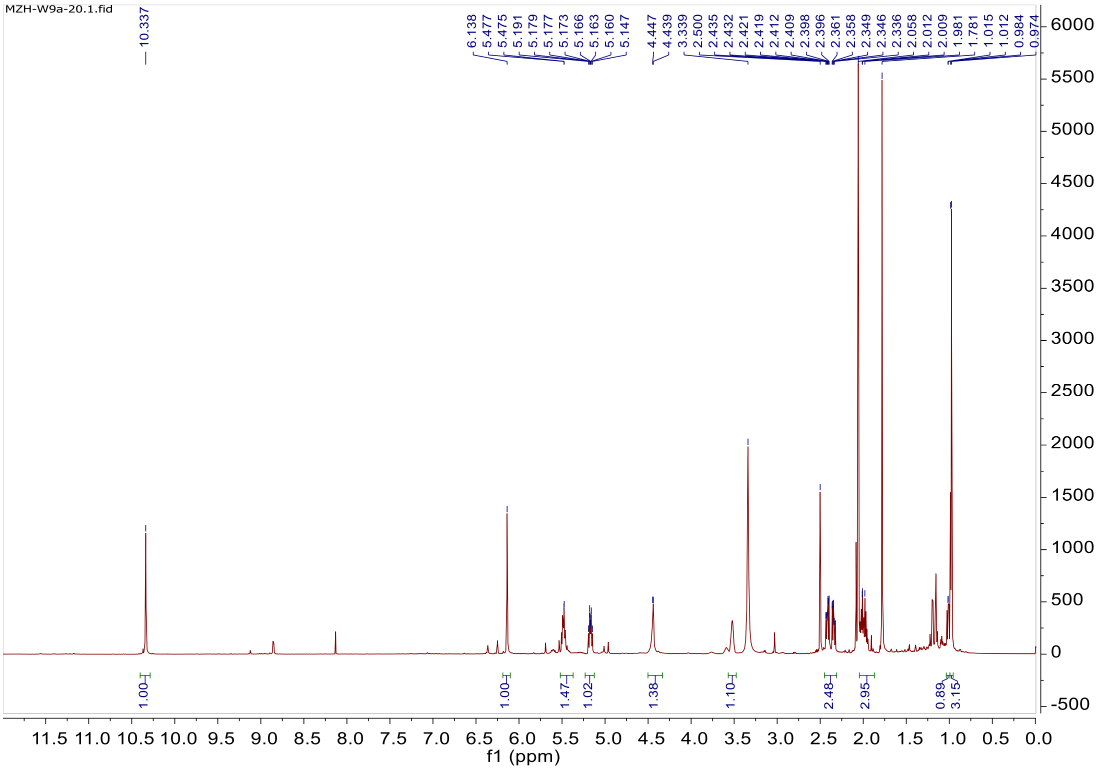

Figure S46. ^{1}H NMR spectrum of **7**

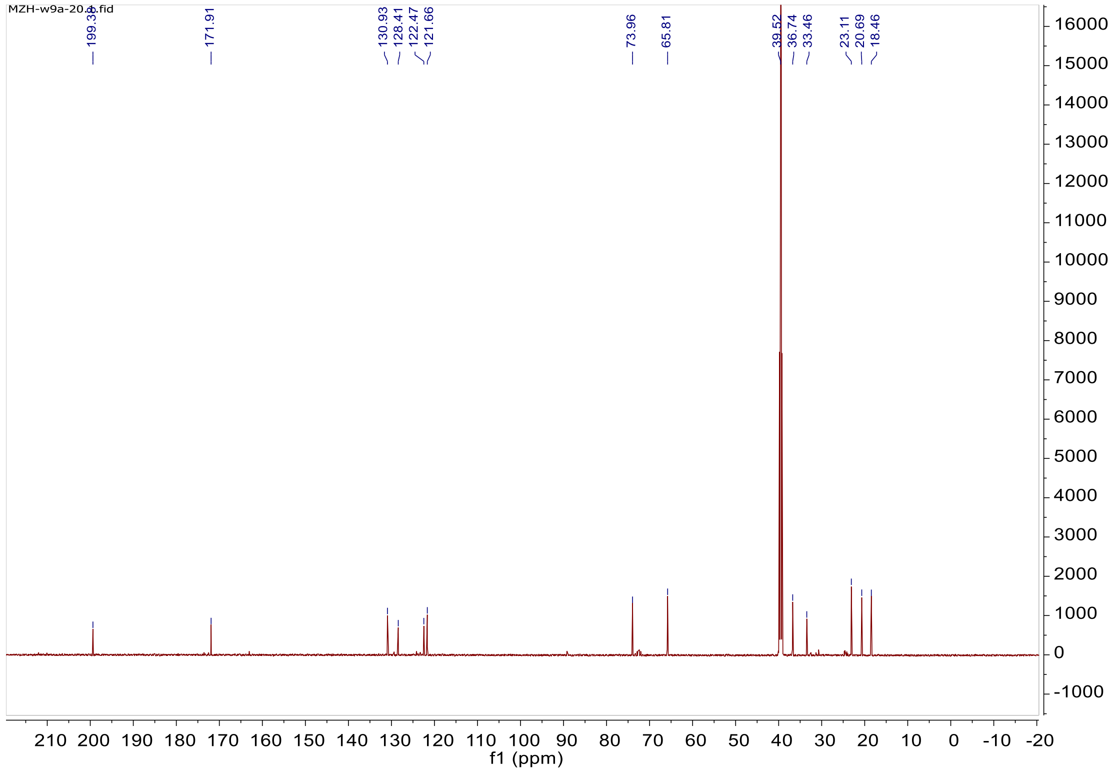

Figure S47. ^{13}C NMR spectrum of **7**

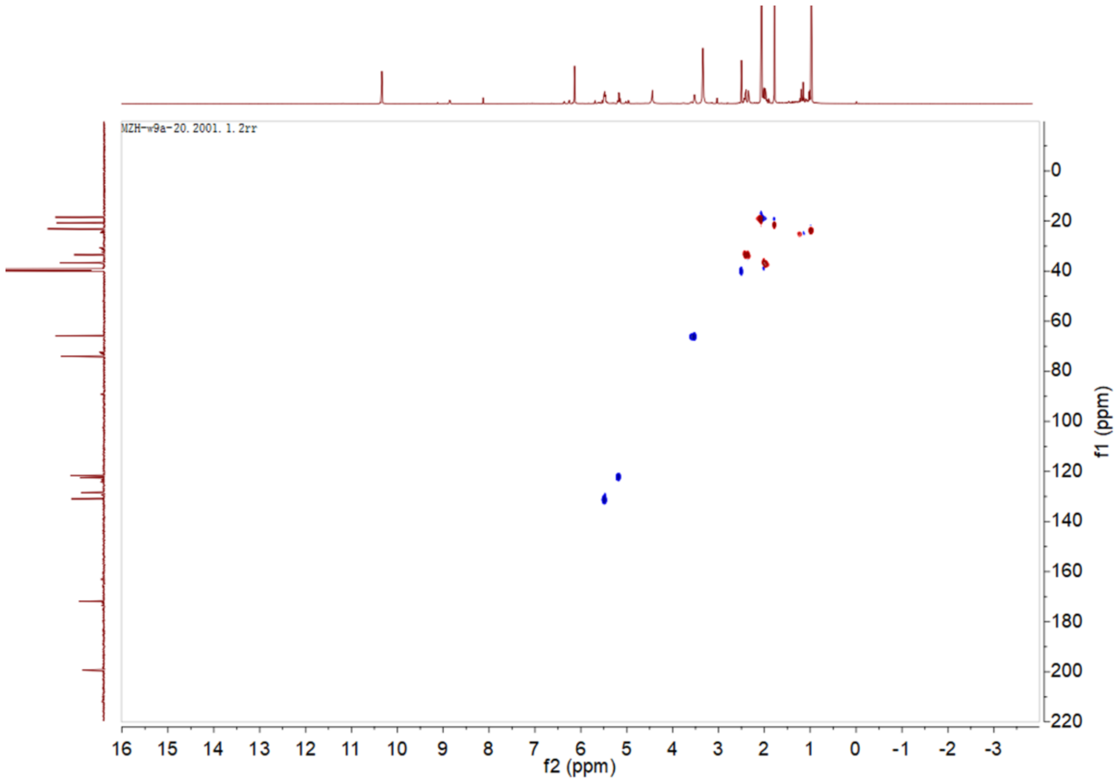

Figure S48. HSQC spectrum of **7**

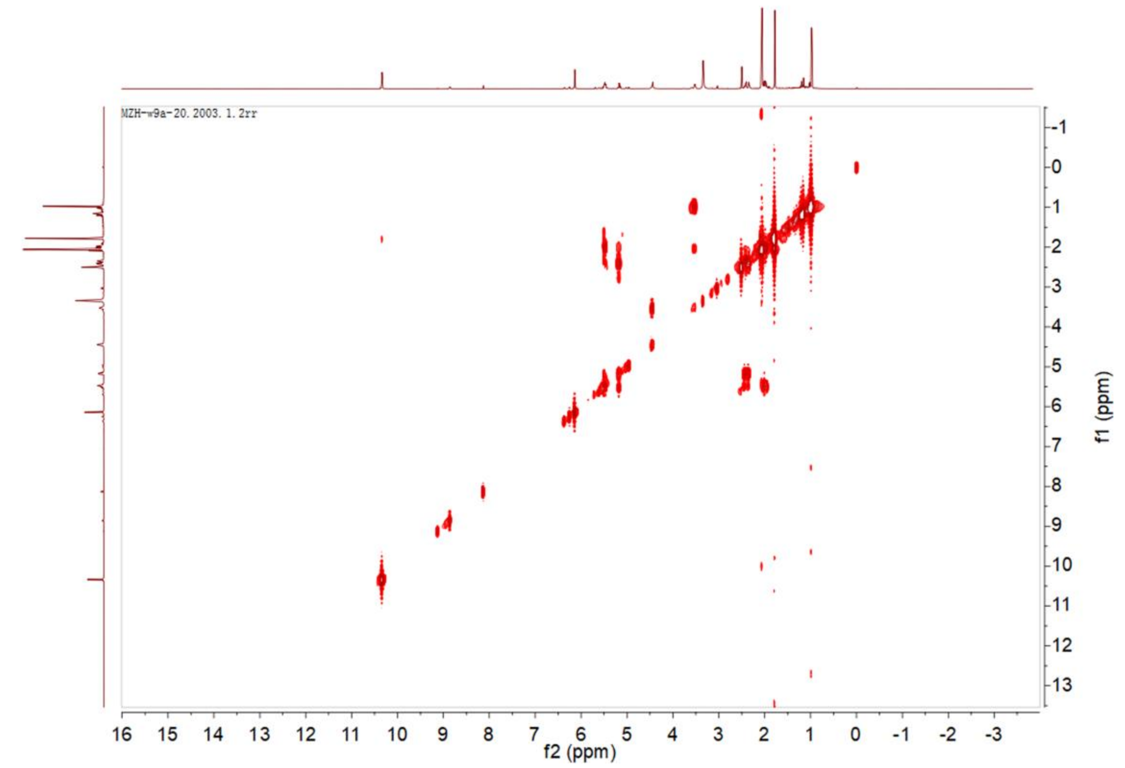

Figure S49. ^{1}H-1H COSY spectrum of **7**

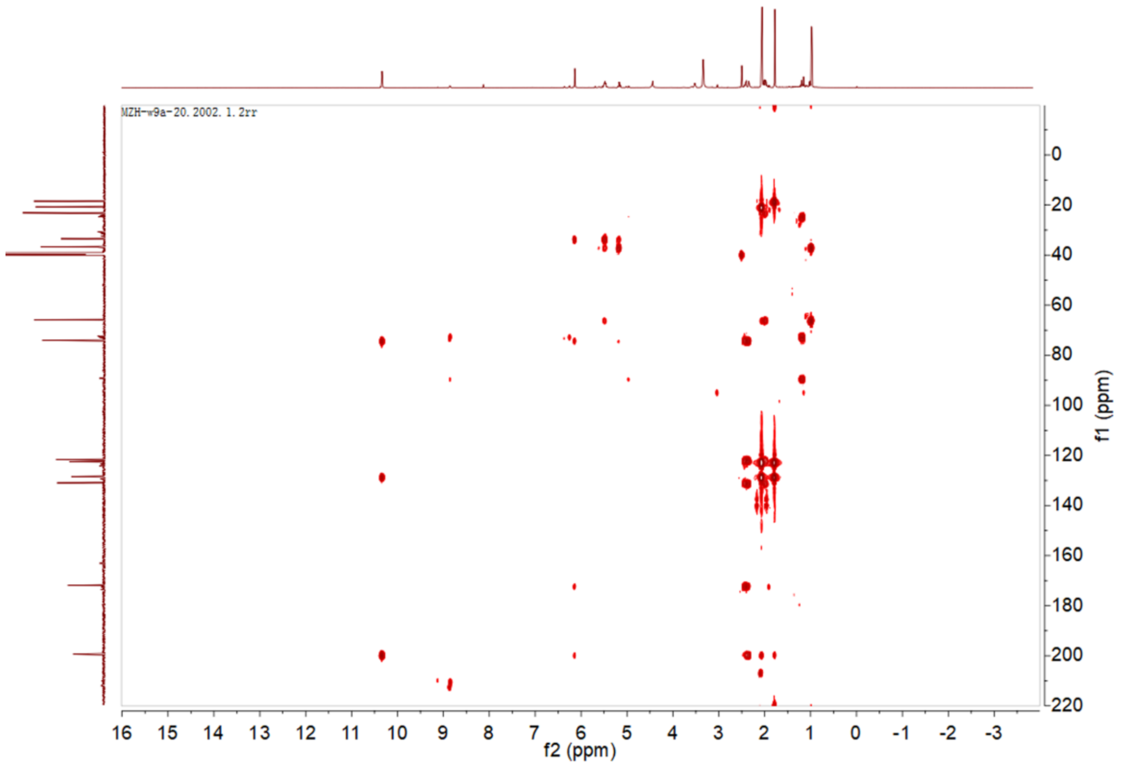

Figure S50. HMBC spectrum of **7**

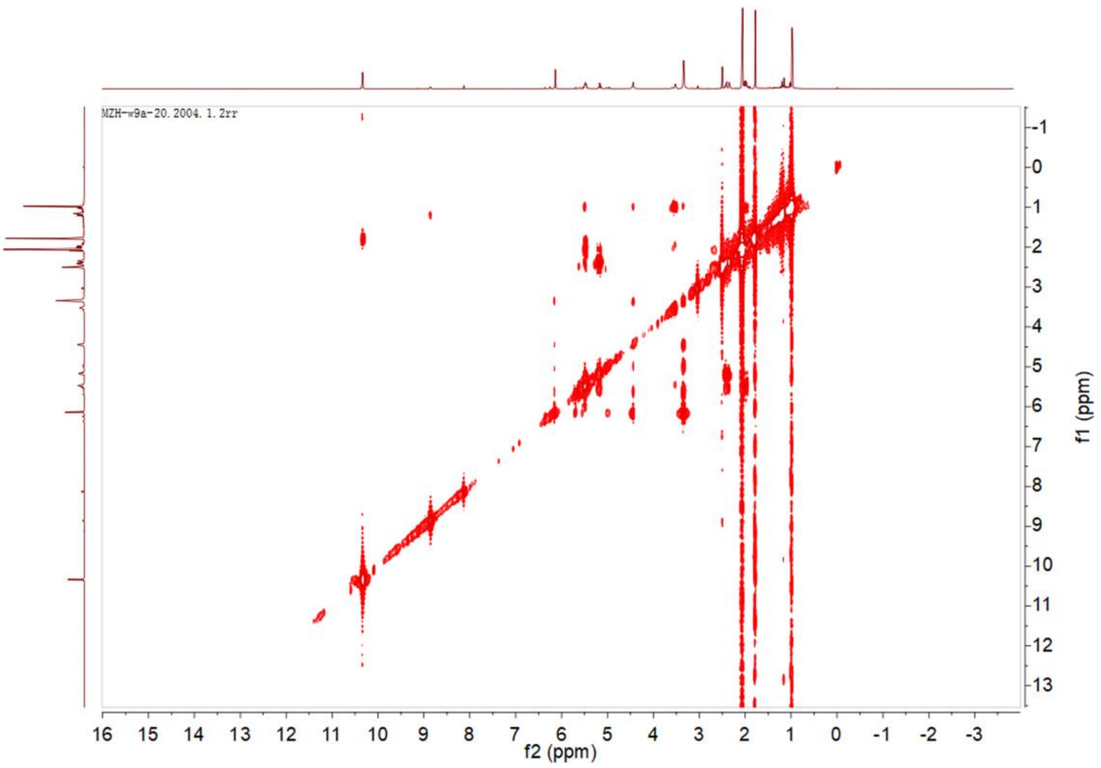

Figure S51. NOESY spectrum of 7

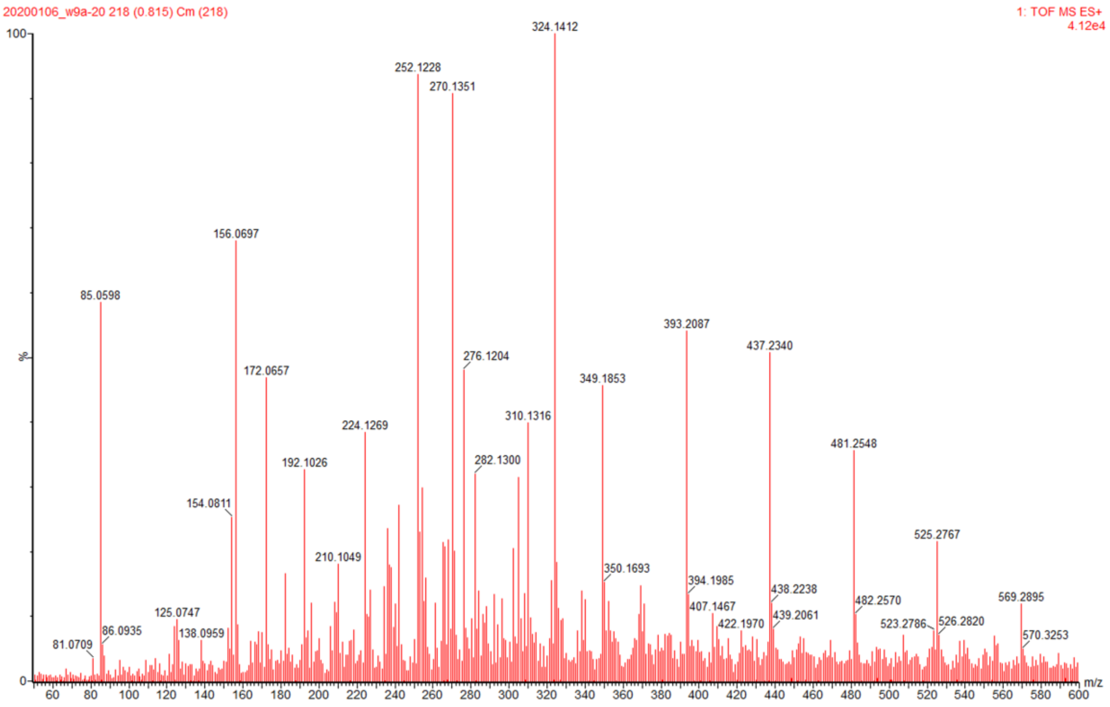

Figure S52. HR-ESI-MS spectrum of 7

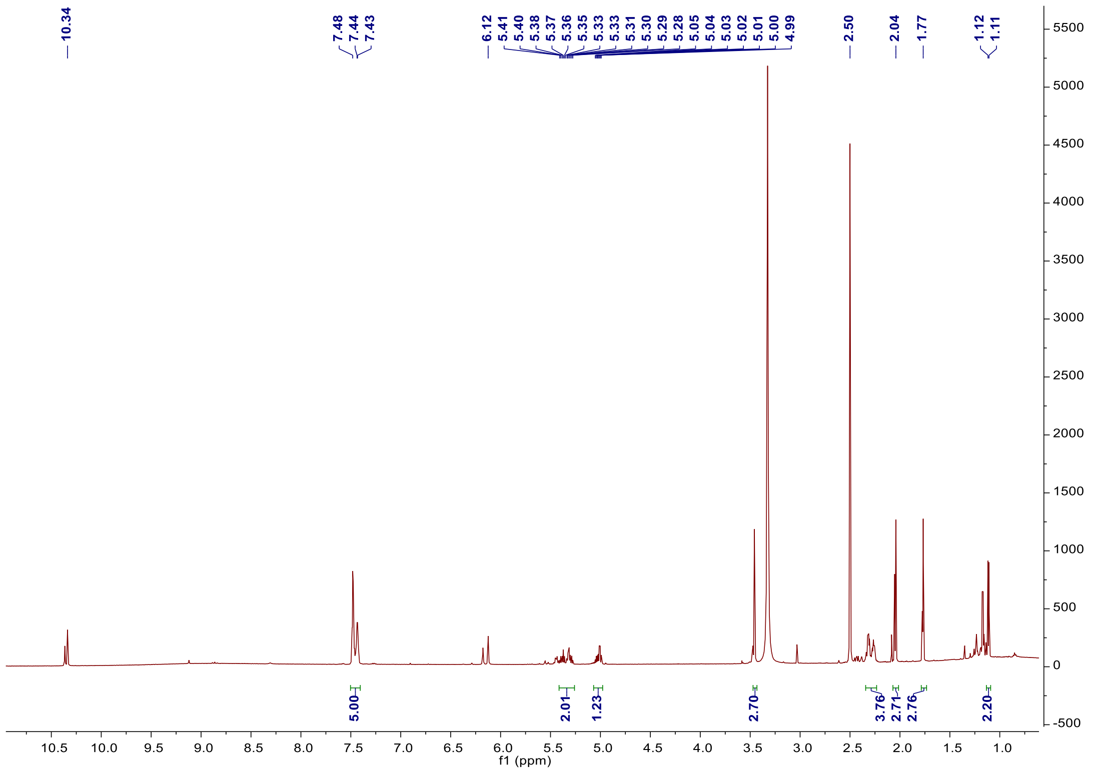

Figure S53. ^{1}H NMR spectrum of (*R*)-MTPA ester of **7**

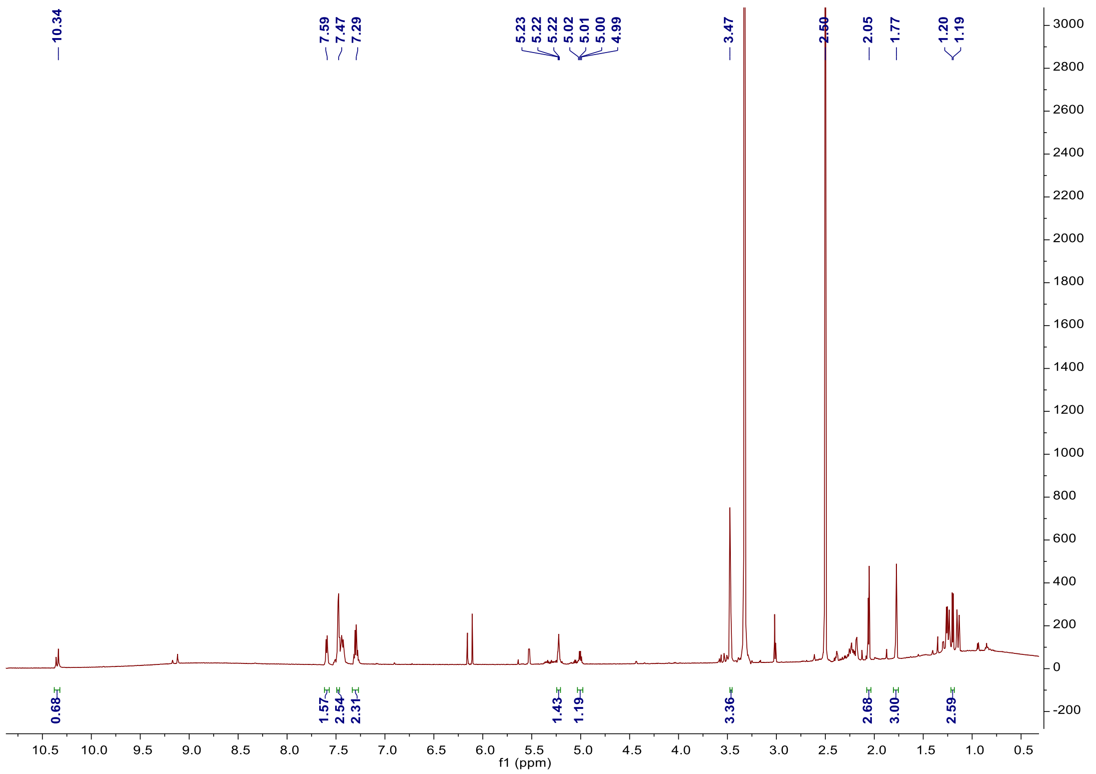

Figure S54. ^{1}H NMR spectrum of (*S*)-MTPA ester of **7**

Table S2. Relative free energies[a] and equilibrium populations[b] of conformers for (3*S*, 6*S*, 10*R*)-2a [c]

Conformer	ΔG	P (%)
**2a1**	0.00	42.97
**2a2**	0.52	17.90
**2a3**	0.78	11.50
**2a4**	0.98	8.26
**2a5**	1.11	6.62
**2a6**	1.11	6.56

[a] B3LYP/6-31G(d), in kcal/mol. [b] From ΔG values at 298.15 K. [c] in MeOH, no imaginary frequency.

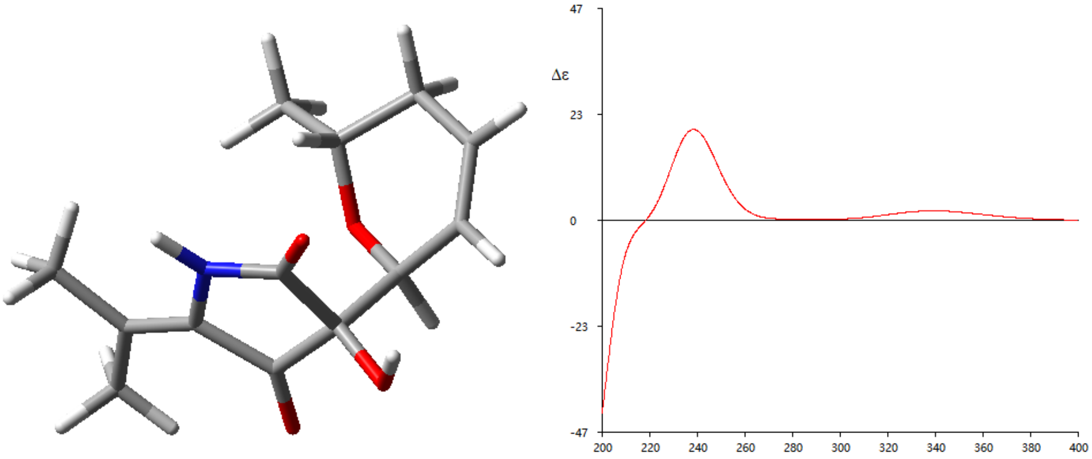

(3*S*, 6*S*, 10*R*)-2**a1**

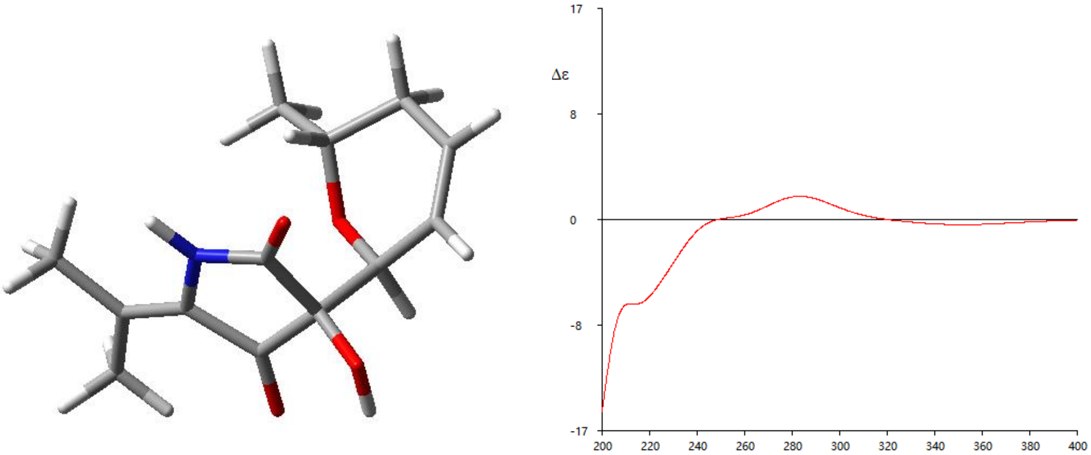

(3*S*, 6*S*, 10*R*)-2**a2**

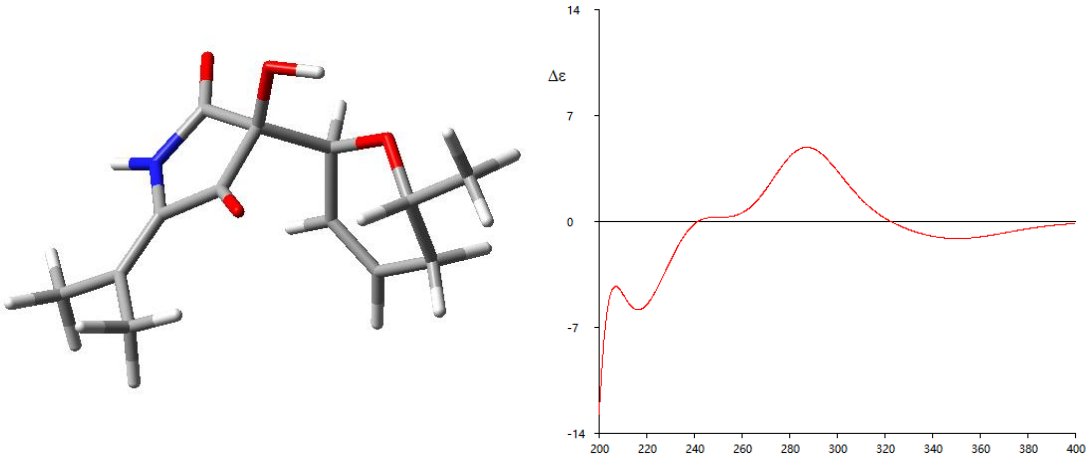

(3*S*, 6*S*, 10*R*)-2**a3**

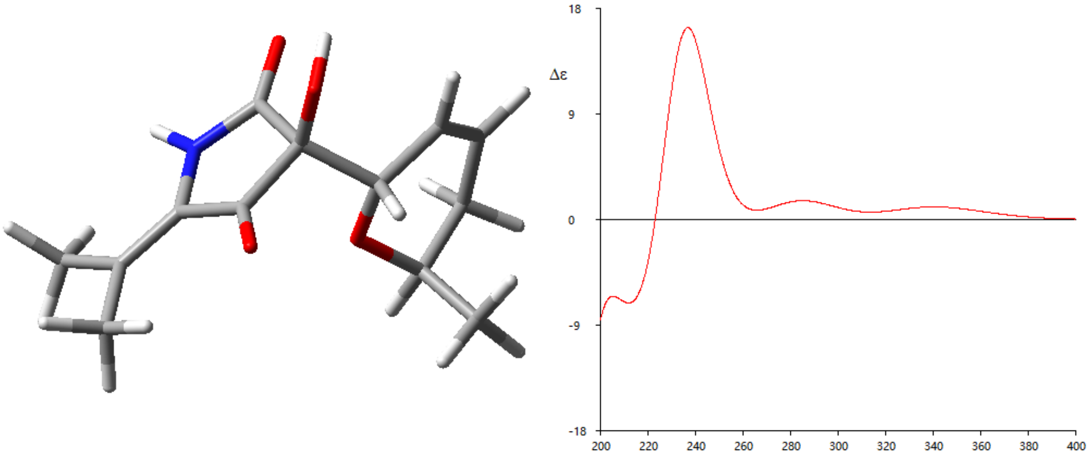

(3*S*, 6*S*, 10*R*)-2**a4**

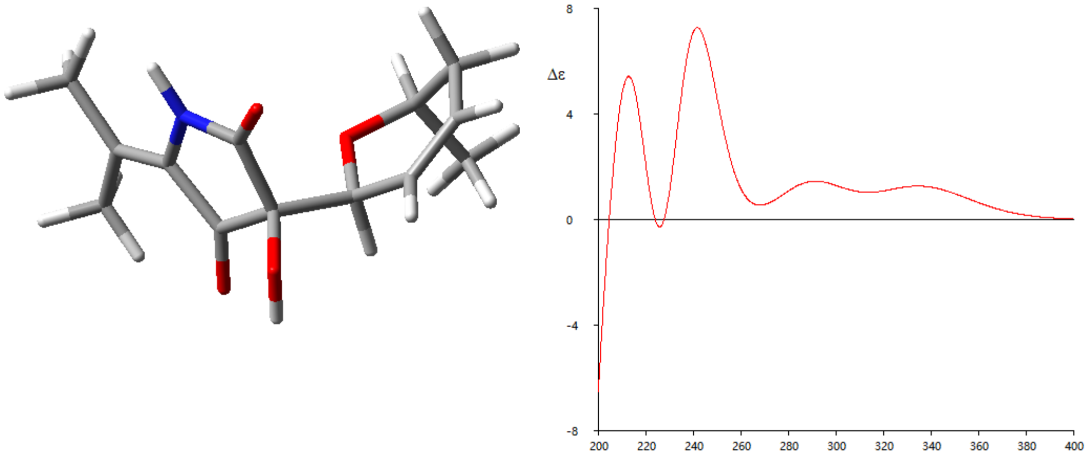

(3*S*, 6*S*, 10*R*)-2**a5**

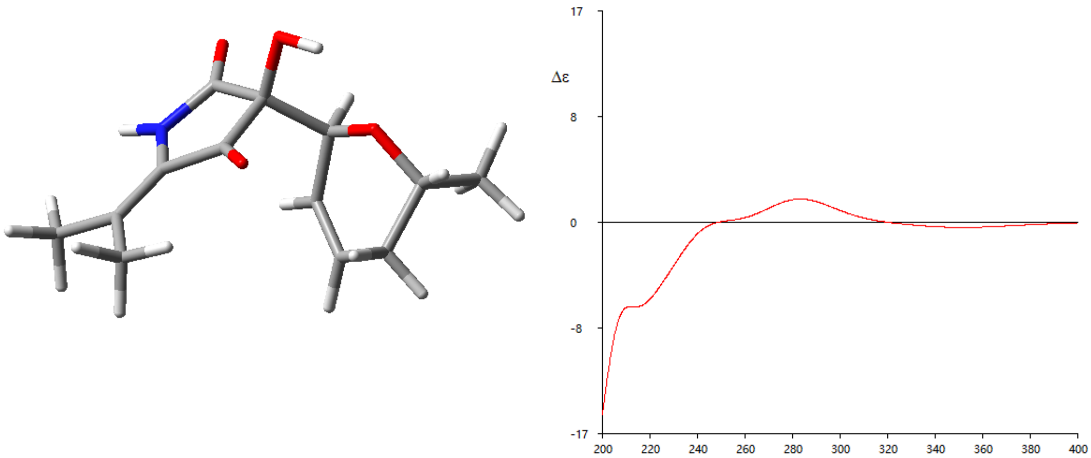

(3*S*, 6*S*, 10*R*)-2**a6**

Figure S55. The optimized structures (left) and the calculated CD spectra of conformers (3*S*, 6*S*, 10*R*)-**2a** in MeOH at M06-2X/def2TZVP level (right). σ=0.30 eV.

Table S3. Relative free energies[a] and equilibrium populations[b] of conformers for (3*S*, 6*R*, 10*S*)-2b[c]

Conformer	Δ*G*	P (%)
**2b1**	0.00	51.42
**2b2**	0.61	18.24
**2b3**	0.65	17.19
**2b4**	0.81	13.15

[a] B3LYP/6-31G(d), in kcal/mol. [b] From Δ*G* values at 298.15 K. [c] in MeOH, no imaginary frequency.

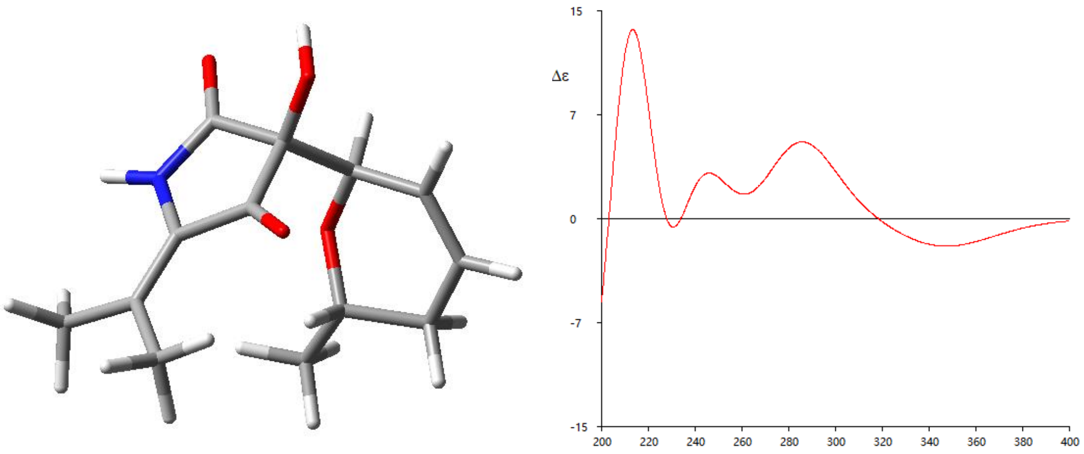

(3*S*, 6*R*, 10*S*)-2**b1**

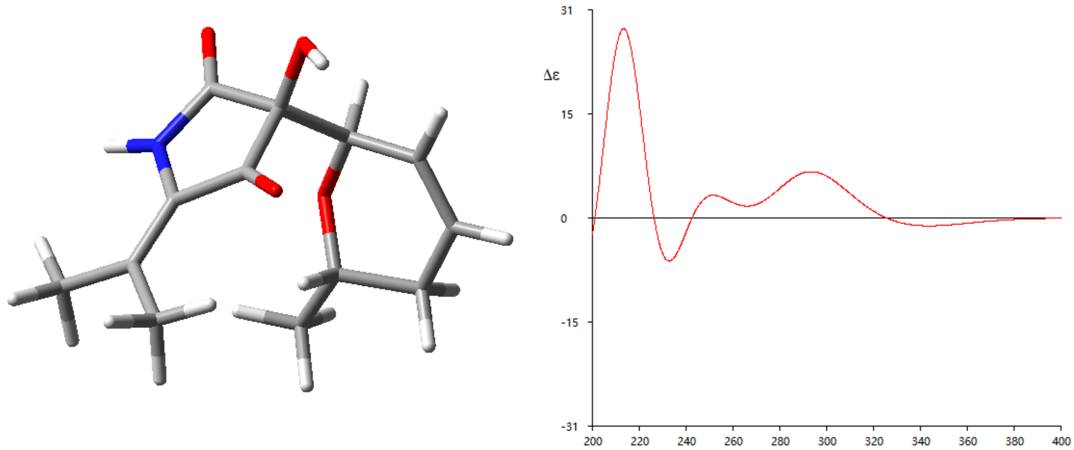

(3*S*, 6*R*, 10*S*)-2**b2**

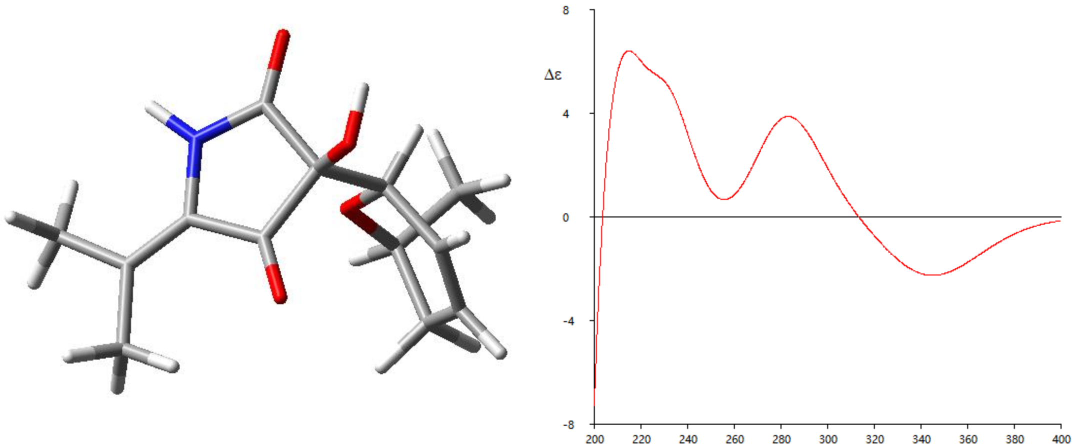

(3*S*, 6*R*, 10*S*)-2**b3**

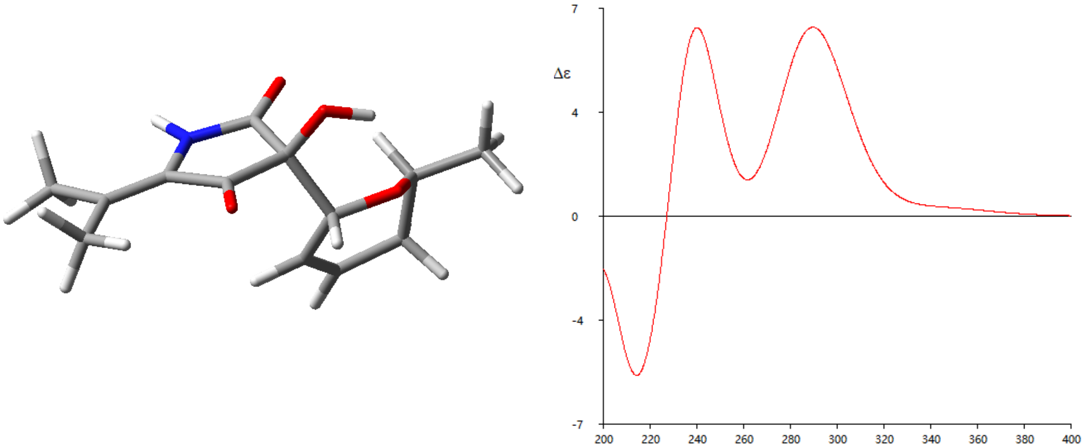

(3*S*, 6*R*, 10*S*)-2**b4**

Figure S56. The optimized structures (left) and the calculated CD spectra of conformers (3*S*, 6*R*, 10*S*)-**2b** in MeOH at M06-2X/def2TZVP level (right). σ=0.30 eV.

Table S4. Relative free energies[a] and equilibrium populations[b] of conformers for (3*S*, 6*S*, 9*R*)-**3a**[c]

Conformer	ΔG	P (%)
**3a1**	0.00	45.78
**3a2**	0.47	20.56
**3a3**	0.48	20.22
**3a4**	0.73	13.45

[a] B3LYP/6-31G(d), in kcal/mol. [b] From ΔG values at 298.15 K. [c] in MeOH, no imaginary frequency.

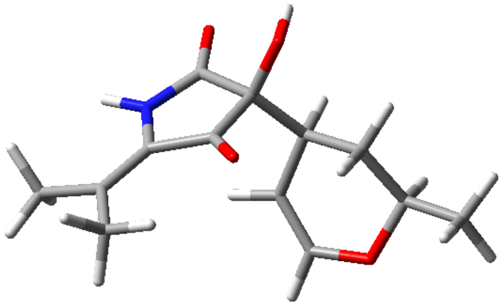

(3*S*, 6*S*, 9*R*)-**3a1**

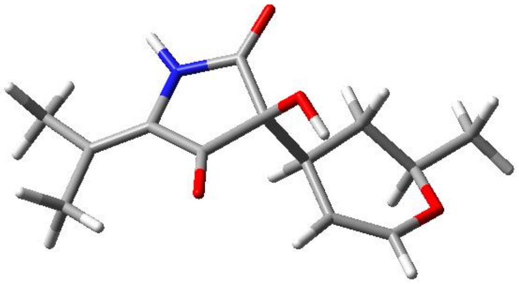

(3*S*, 6*S*, 9*R*)-**3a2**

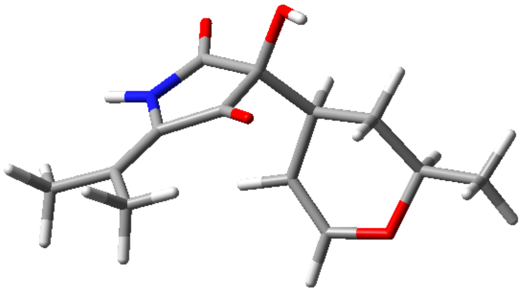

(3*S*, 6*S*, 9*R*)-**3a3**

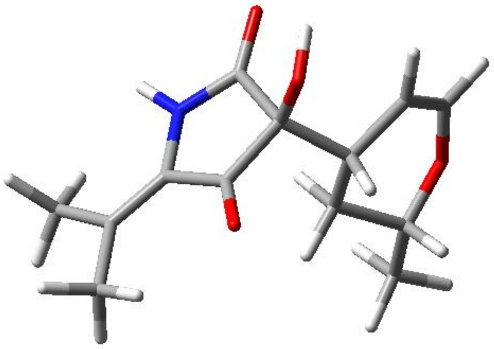

(3*S*, 6*S*, 9*R*)-**3a4**

Figure S57. The optimized structure of conformers for (3*S*, 6*S*, 9*R*)-**3a** in MeOH at B3LYP/6-31G (d) level

Table S5. Relative free energies[a] and equilibrium populations[b] of conformers for (3*S*, 6*R*, 9*S*)-3b[c]

Conformer	ΔG	P (%)
**3b1**	0.00	46.24
**3b2**	0.36	25.13
**3b3**	0.52	19.29
**3b4**	0.95	9.34

[a] B3LYP/6-31G(d), in kcal/mol. [b] From ΔG values at 298.15 K. [c] in MeOH, no imaginary frequency.

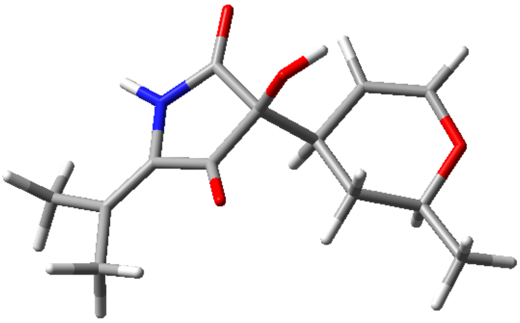

(3*S*, 6*R*, 9*S*)-**3b1**

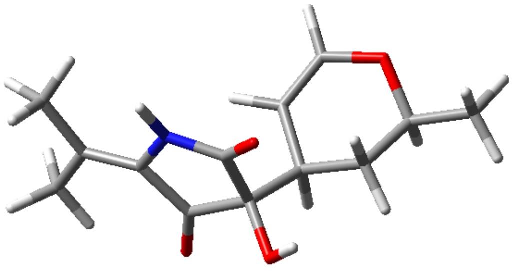

(3*S*, 6*R*, 9*S*)-**3b2**

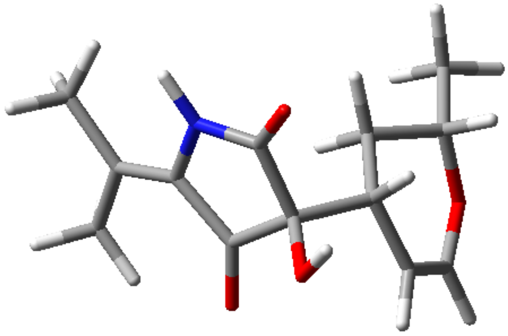

(3*S*, 6*R*, 9*S*)-**3b3**

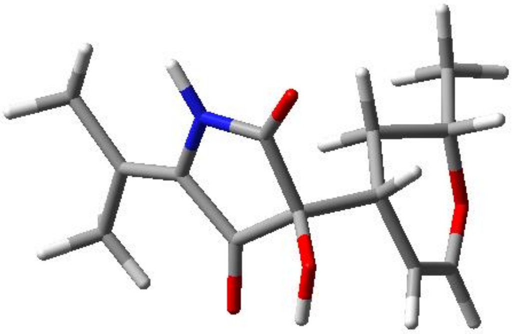

(3*S*, 6*R*, 9*S*)-**3b4**

Figure S58. The optimized structure of conformers for (3*S*, 6*R*, 9*S*)-**3b** in MeOH at B3LYP/6-31G (d) level

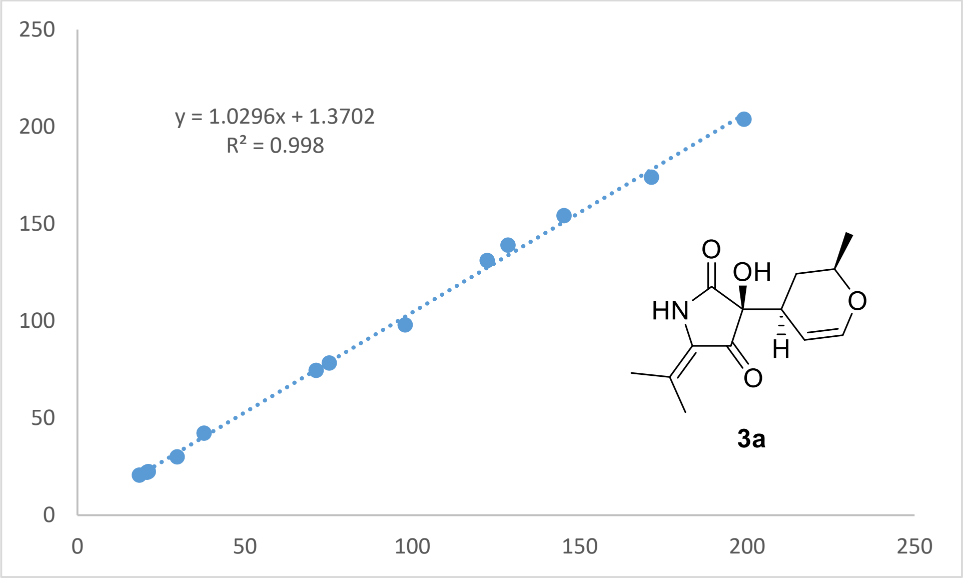

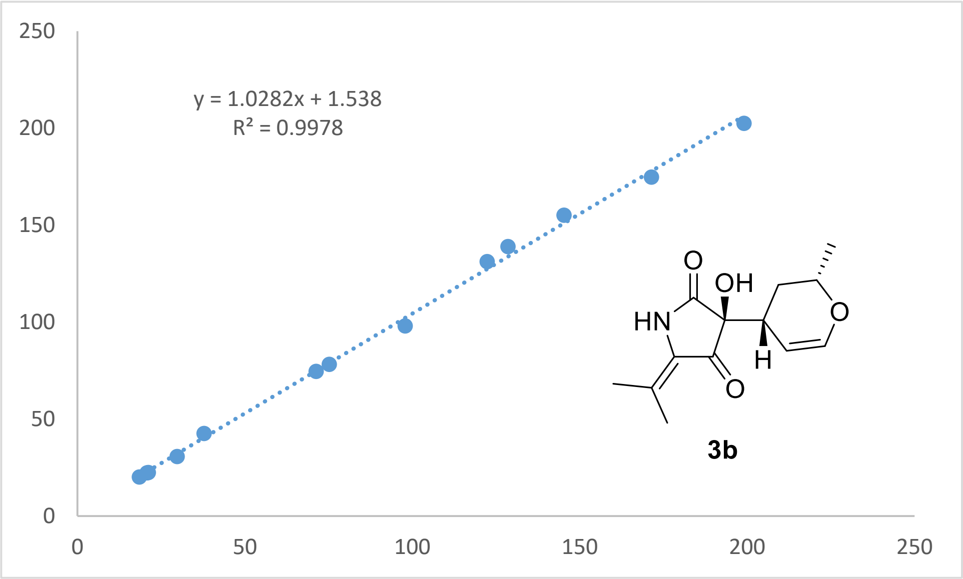

Figure S59. Correlation plots of experimental ^{13}C NMR chemical shifts versus the corresponding calculated data for (3*S*, 6*S*, 9*R*)-**3a** and (3*S*, 6*R*, 9*S*)-**3b**

		DP4+	89.66%	10.34%
Nuclei	sp2?	xperimenta	Isomer 1	Isomer 2
C	x	122.3	131.118	131.161
C	x	199.04	203.806	202.455
C		75.14	78.319	78.102
C	x	171.35	174.002	174.743
C	x	128.57	139.058	138.82
C		18.49	20.49	20.138
C		20.71	22.077	22.151
C		37.73	42.155	42.442
C	x	97.81	97.961	98.007
C	x	145.33	154.144	155.104
C		71.2	74.538	74.579
C		29.7	30.025	30.613
C		21.17	22.413	22.364

Figure S60. DP4+ probabilities (%) for conformers (3*S*, 6*S*, 9*R*)-**3a** (isomer 1) and (3*S*, 6*R*, 9*S*)-**3b** (isomoer 2)

Table S6. Relative free energies[a] and equilibrium populations[b] of conformers for (3*S*,6*S*,9*S*)-**4a**[c]

Conformer	Δ*G*	P (%)
**4a1**	0.00	56.61
**4a2**	0.35	31.47
**4a3**	1.30	6.28
**4a4**	1.37	5.64

[a] B3LYP/6-31G(d), in kcal/mol. [b] From Δ*G* values at 298.15 K. [c] in MeOH, no imaginary frequency.

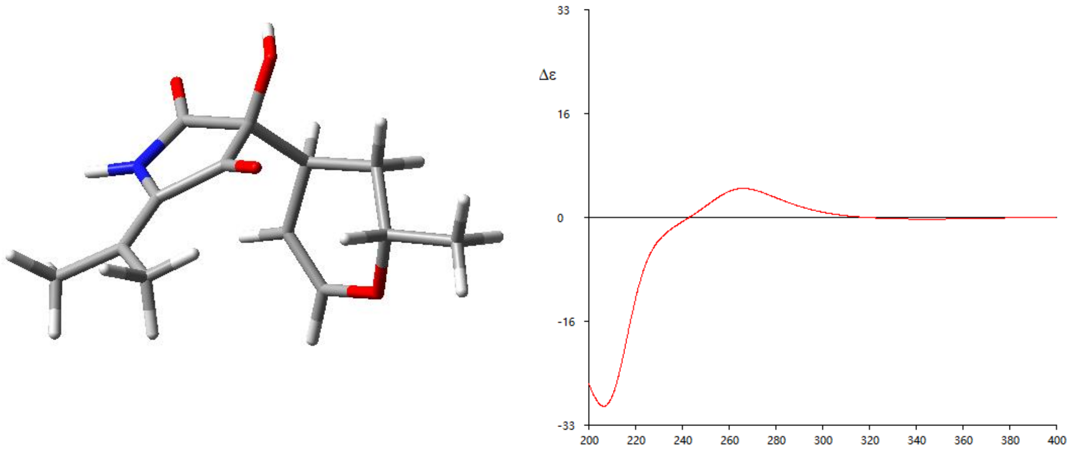

(3*S*,6*S*,9*S*)-4**a1**

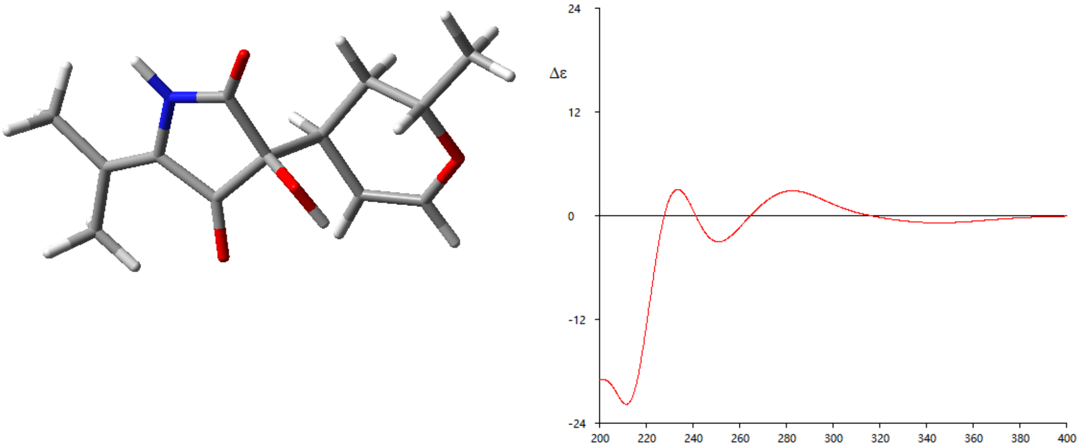

(3*S*,6*S*,9*S*)-4**a2**

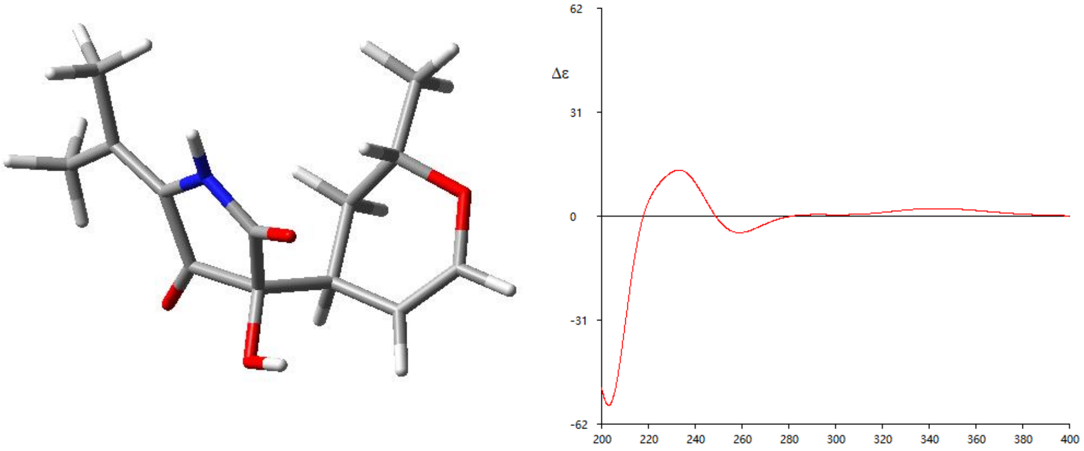

(3*S*,6*S*,9*S*)-4**a3**

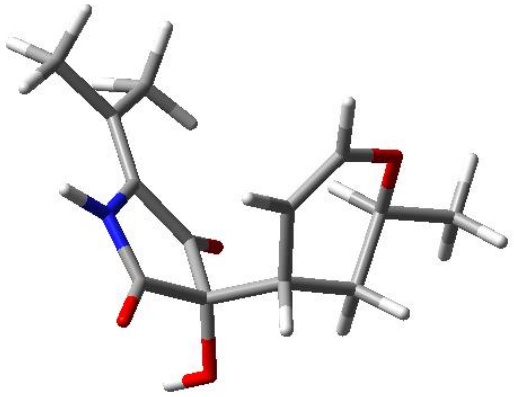
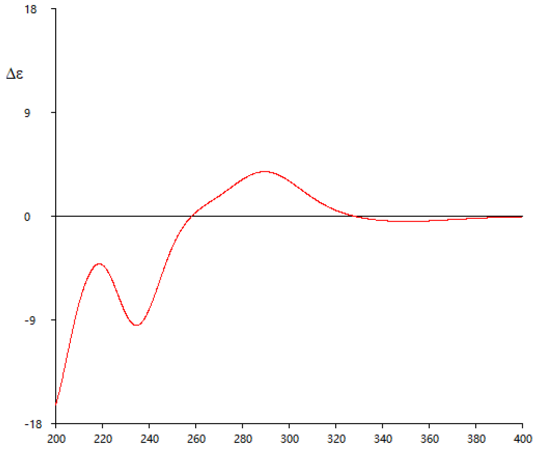

(3*S*,6*S*,9*S*)-4**a4**

Figure S61. The optimized structures (left) and the calculated CD spectra for conformers (3*S*,6*S*,9*S*)-**4a** in MeOH at M06-2X/def2TZVP level (right). σ=0.30 eV.

Table S7. Relative free energies[a] and equilibrium populations[b] of conformers for (3*S*,6*R*,9*R*)-**4b**[c]

Conformer	Δ*G*	P (%)
**4b1**	0.00	58.58
**4b2**	0.34	32.79
**4b3**	1.13	8.63

[a] B3LYP/6-31G(d), in kcal/mol. [b] From Δ*G* values at 298.15 K. [c] in MeOH, no imaginary frequency.

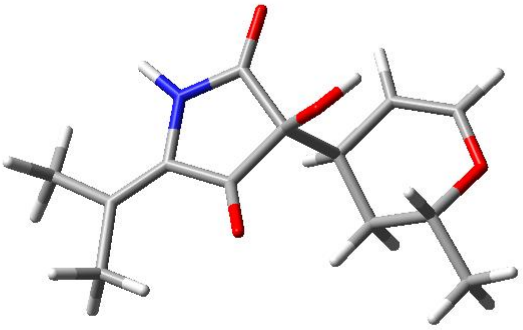
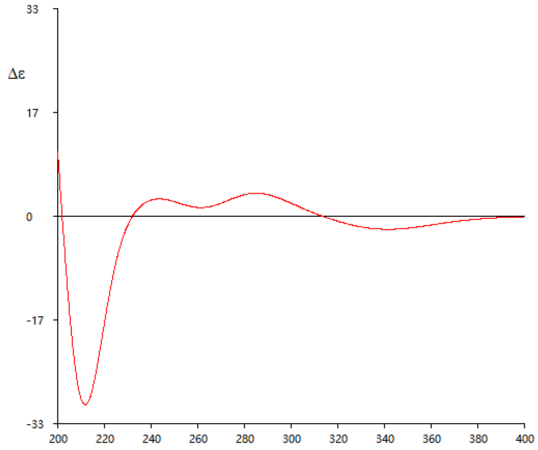

(3*S*,6*R*,9*R*)-**4b1**

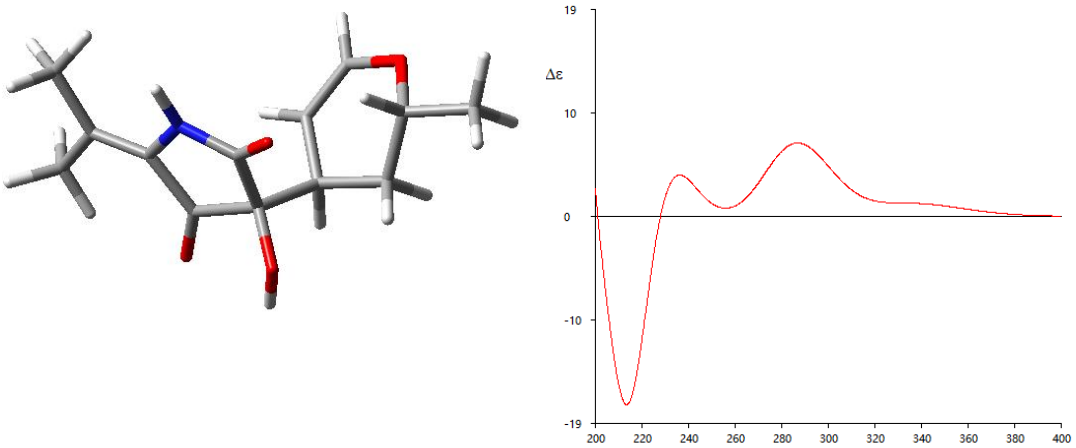

(3*S*,6*R*,9*R*)-**4b2**

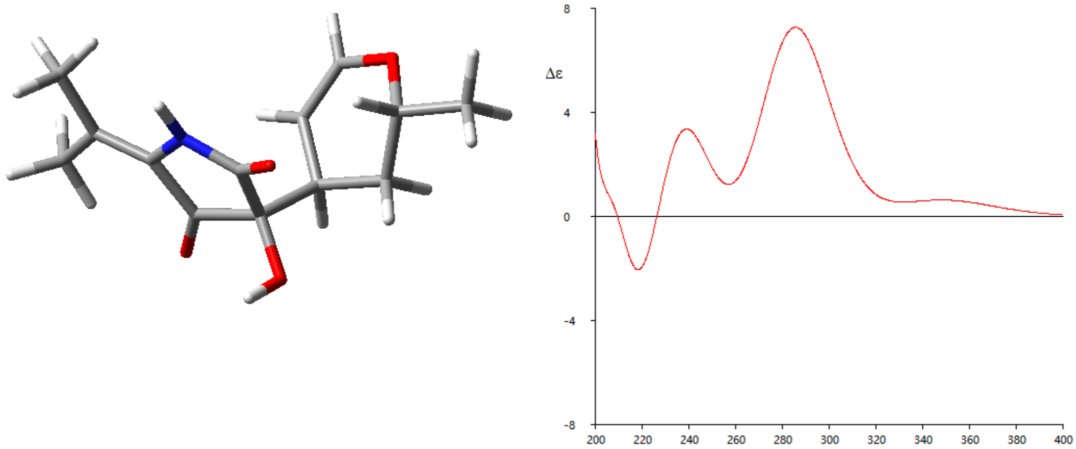

(3*S*,6*R*,9*R*)-**4b3**

Figure S62. The optimized structures (left) and the calculated CD spectra for conformers (3*S*,6*R*,9*R*)-**4b** in MeOH at M06-2X/def2TZVP level (right). σ=0.30 eV.

Table S8. Relative free energies[a] and equilibrium populations[b] of conformers for (3*R*,6*R*,9*R*)-**4c**[c]

Conformer	Δ*G*	P (%)
**4c1**	0.00	57.30
**4c2**	0.36	31.09
**4c3**	1.34	5.95
**4c4**	1.37	5.65

[a] B3LYP/6-31G(d), in kcal/mol. [b] From Δ*G* values at 298.15 K. [c] in MeOH, no imaginary frequency.

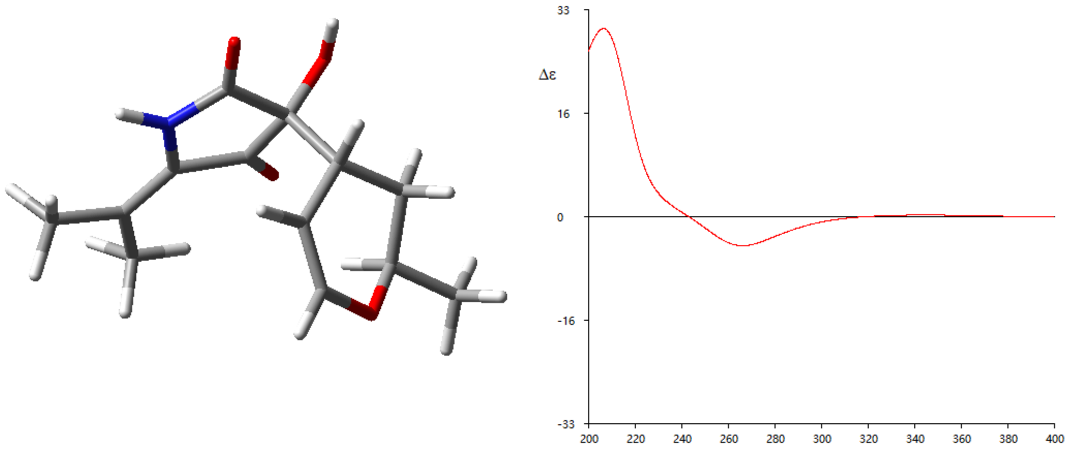

(3*R*,6*R*,9*R*)-**4c1**

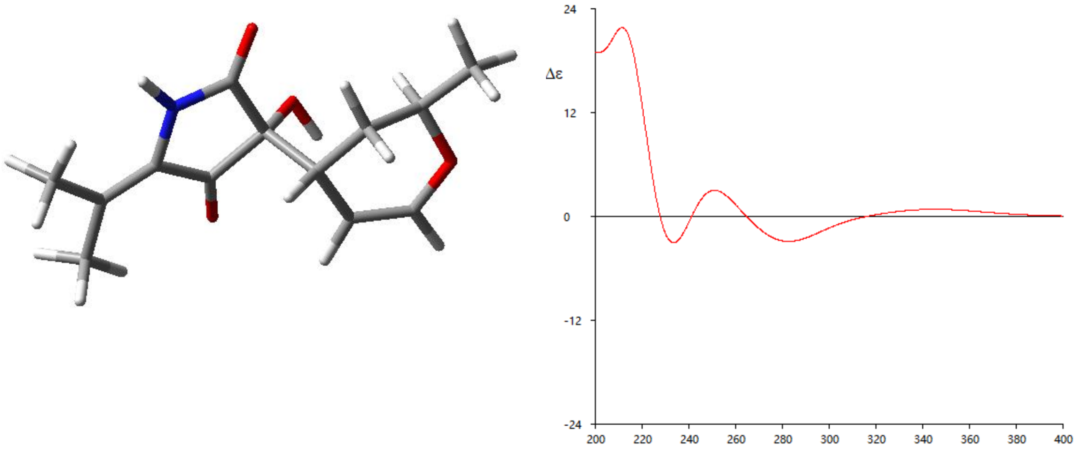

(3*R*,6*R*,9*R*)-**4c2**

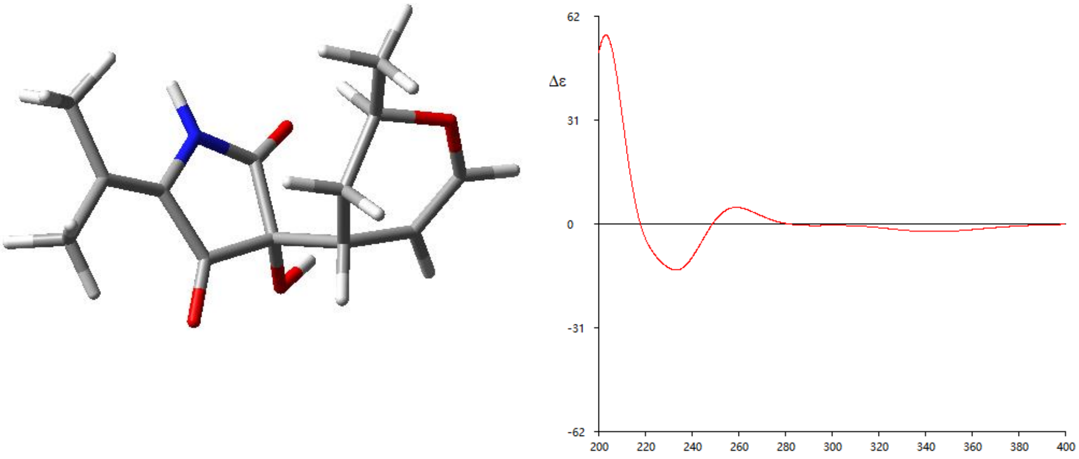

(3*R*,6*R*,9*R*)-**4c3**

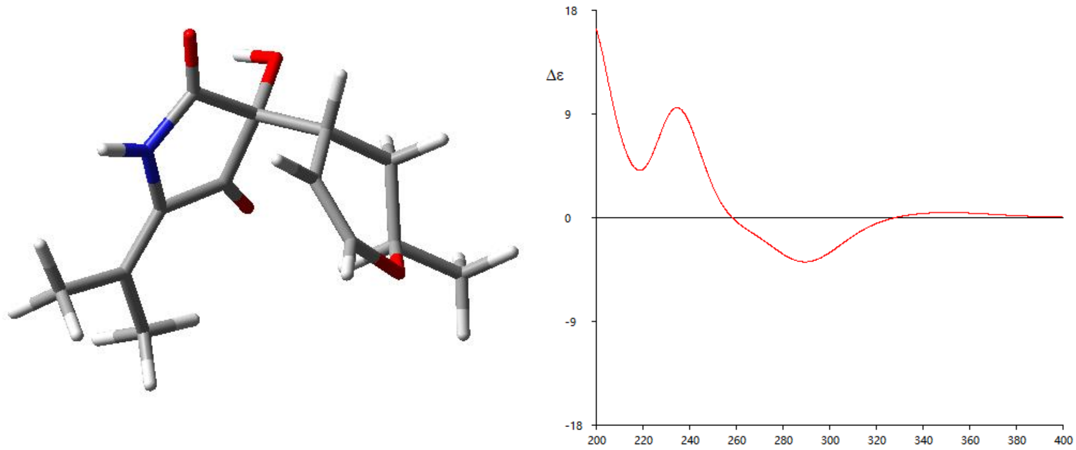

(3*R*,6*R*,9*R*)-**4c4**

Figure S63. The optimized structures (left) and the calculated CD spectra for conformers (3*R*,6*R*,9*R*)-**4c** in MeOH at M06-2X/def2TZVP level (right). σ=0.30 eV.

Table S9. Relative free energies[a] and equilibrium populations[b] of conformers for (3*R*,6*S*,9*S*)-**4d**[c]

Conformer	Δ*G*	P (%)
**4d1**	0.00	54.38
**4d2**	0.34	30.43
**4d4**	1.13	8.00
**4d4**	1.20	7.19

[a] B3LYP/6-31G(d), in kcal/mol. [b] From Δ*G* values at 298.15 K. [c] in MeOH, no imaginary frequency.

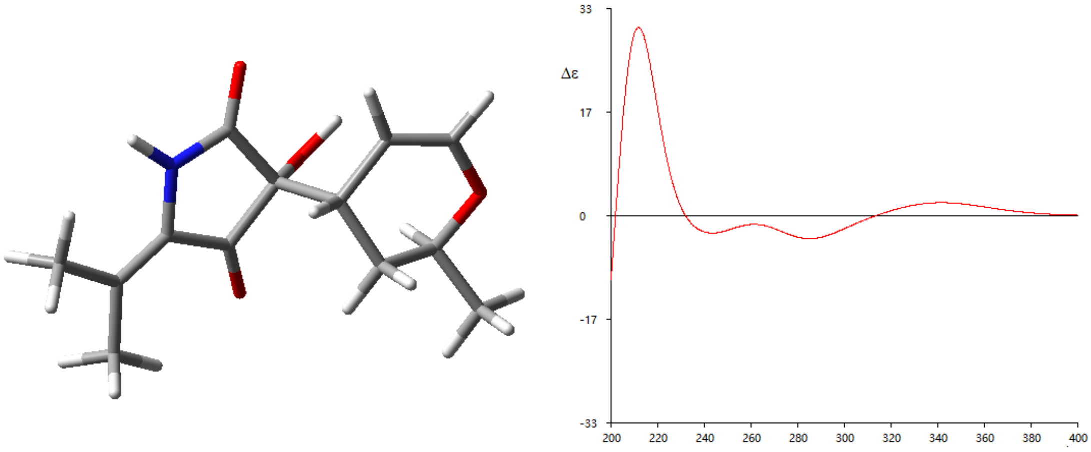

(3*R*,6*S*,9*S*)-**4d1**

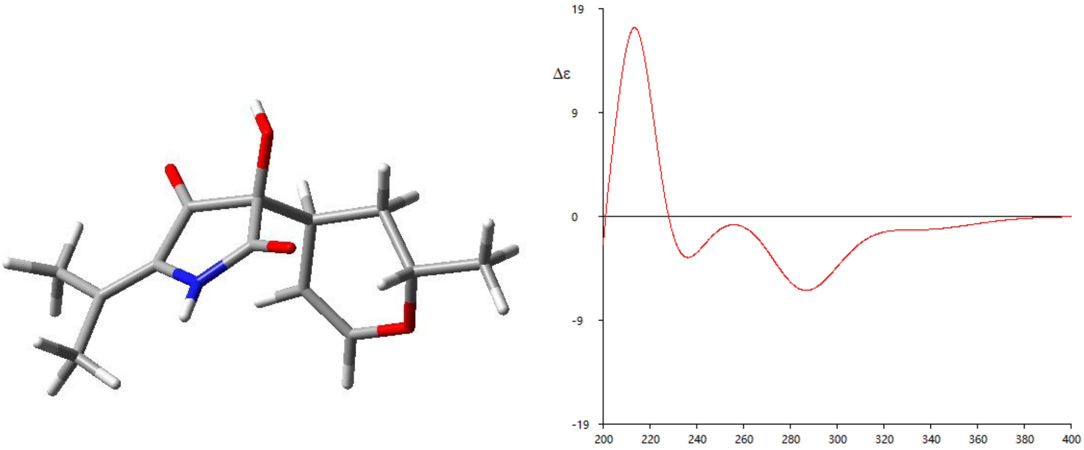

(3*R*,6*S*,9*S*)-**4d2**

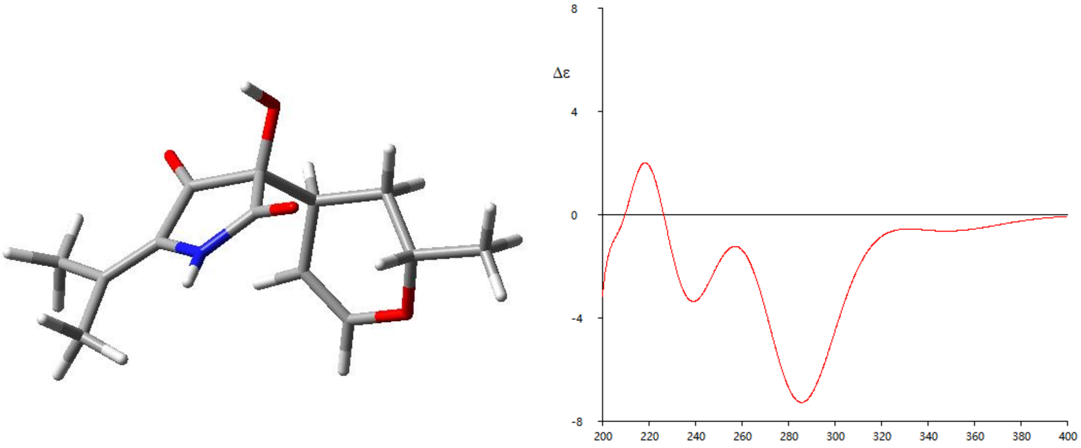

(3*R*,6*S*,9*S*)-**4d3**

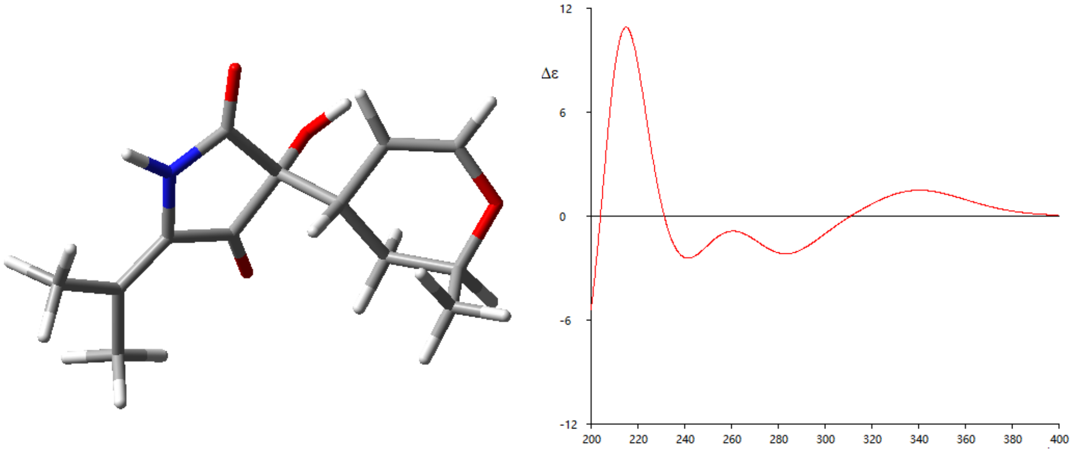

(3*R*,6*S*,9*S*)-**4d4**

Figure S64. The optimized structures (left) and the calculated CD spectra for conformers (3*R*,6*S*,9*S*)-**4d** in MeOH at M06-2X/def2TZVP level (right). σ=0.30 eV.

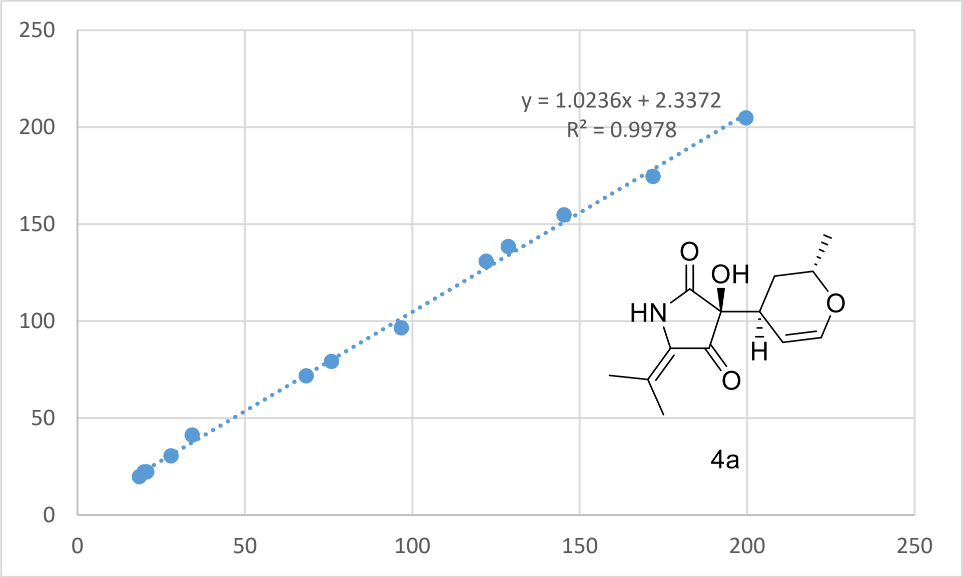

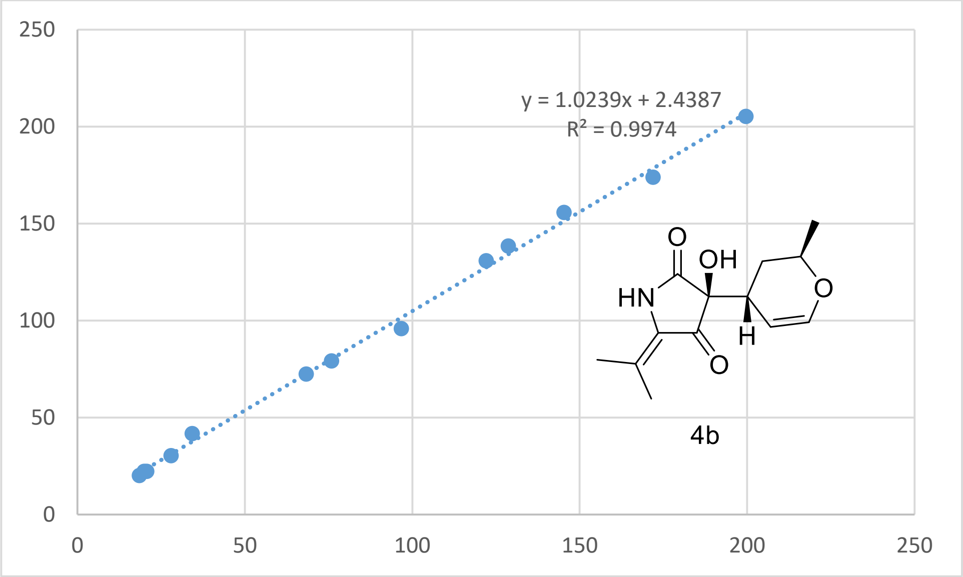

Figure S65. Correlation plots of experimental ^{13}C NMR chemical shifts versus the corresponding calculated data for (3*S*, 6*S*, 9*S*)-**4a** and (3*S*, 6*R*, 9*R*)-**4b**

		DP4+	91.53%	8.47%
Nuclei	sp2?	xperimenta	Isomer 1	Isomer 2
C	x	122.07	130.833	130.781
C	x	199.64	204.715	205.3
C		75.86	79.184	79.225
C	x	171.89	174.492	173.923
C	x	128.6	138.448	138.369
C		18.44	19.821	20.057
C		19.91	22.225	22.203
C		34.26	41.124	41.651
C	x	96.65	96.549	95.898
C	x	145.25	154.707	155.726
C		68.28	71.742	72.405
C		27.95	30.437	30.371
C		20.63	22.192	22.205

Figure S66. DP4+ probabilities (%) for conformers of (3*S*, 6*S*, 9*S*)-**4a** (isomer 1) and (3*S*, 6*R*, 9*R*)-**4b** (isomoer 2).

Table S10. Relative free energies[a] and equilibrium populations[b] of conformers for (3*S*, 6*R*, 8S, 9*S*)-**5a**[c]

Conformer	Δ*G*	P (%)
**5a1**	0.00	24.26
**5a2**	0.10	20.40
**5a3**	0.15	18.97
**5a4**	0.17	18.20
**5a5**	0.17	18.17

[a] B3LYP/6-31G(d), in kcal/mol. [b] From Δ*G* values at 298.15 K. [c] in MeOH, no imaginary frequency.

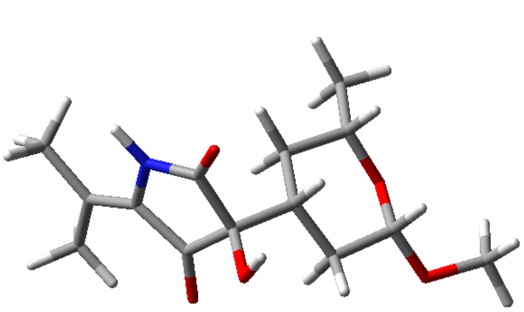

(3*S*,6*R*,8*S*,9*S*)-**5a1**

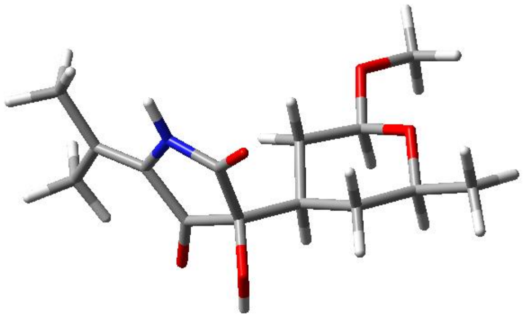

(3*S*,6*R*,8*S*,9*S*)-**5a2**

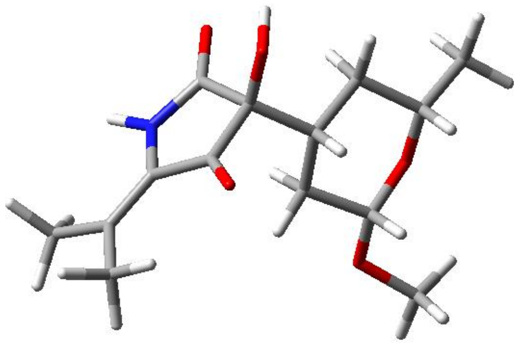

(3*S*,6*R*,8*S*,9*S*)-**5a3**

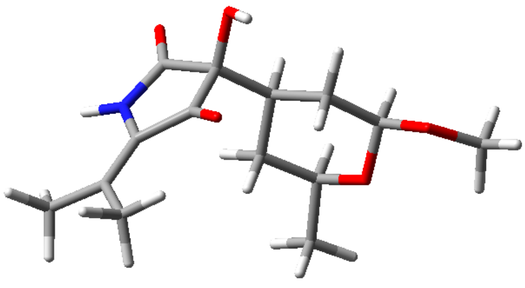

(3*S*,6*R*,8*S*,9*S*)-**5a4**

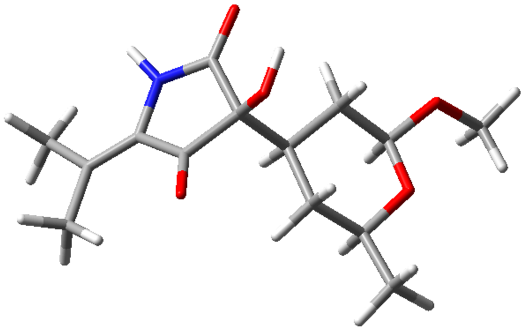

(3*S*,6*R*,8*S*,9*S*)-**5a5**

Figure S67. The optimized structure of conformers for (3*S*,6*R*,8*S*,9*S*)-**5a** in MeOH at B3LYP/6-31G (d) level

Table S11. Relative free energies[a] and equilibrium populations[b] of conformers for (3*S*,6*S*,8*R*,9*R*)-**5b** [c]

Conformer	Δ*G*	P (%)
**5b1**	0.00	48.08
**5b2**	0.32	28.22
**5b3**	0.65	16.14
**5b4**	1.10	7.56

[a] B3LYP/6-31G(d), in kcal/mol. [b] From Δ*G* values at 298.15 K. [c] in MeOH, no imaginary frequency.

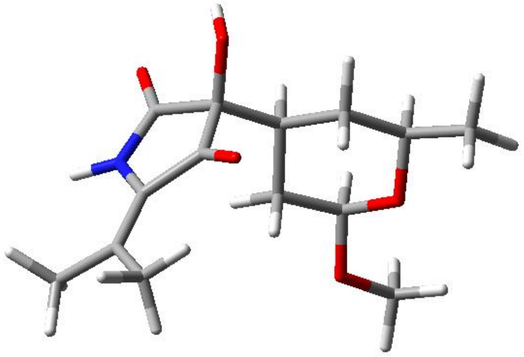

(3*S*,6*S*,8*R*,9*R*)-**5b1**

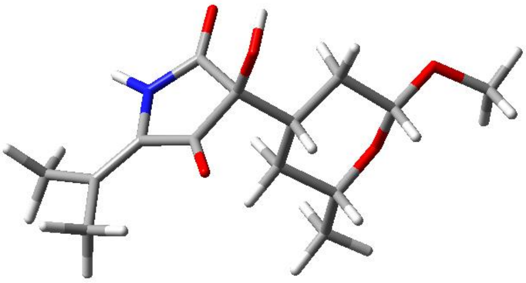

(3*S*,6*S*,8*R*,9*R*)-**5b2**

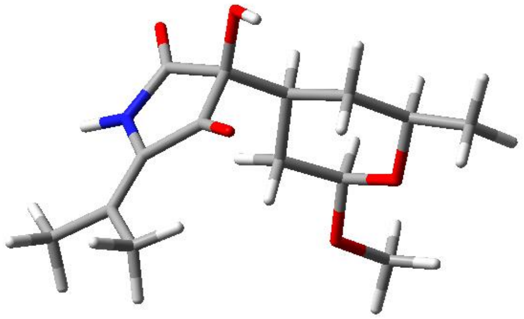

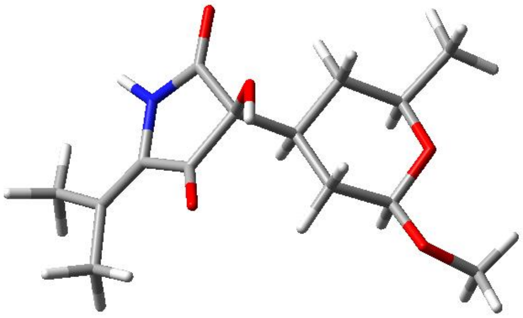

(3*S*,6*S*,8*R*,9*R*)-**5b3** (3*S*,6*S*,8*R*,9*R*)-**5b4**

Figure S68. The optimized structure of conformers for (3*S*,6*S*,8*R*,9*R*)-**5b** in MeOH at B3LYP/6-31G (d) level

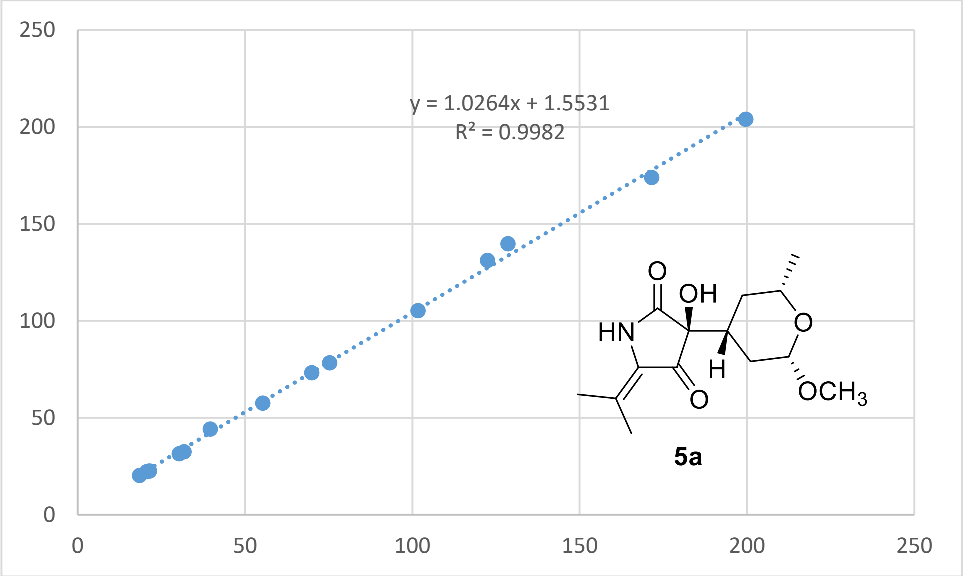

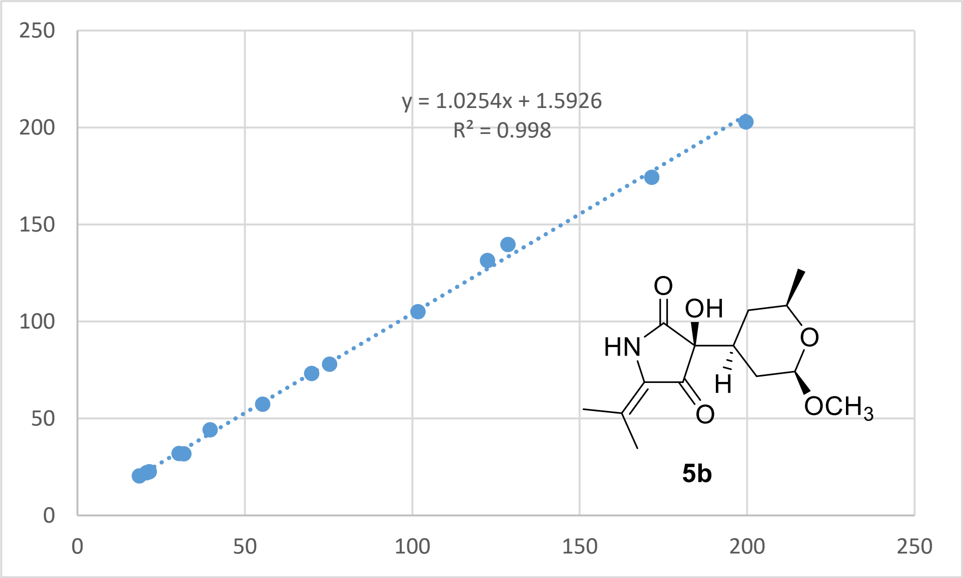

Figure S69. Correlation plots of experimental ^{13}C NMR chemical shifts versus the corresponding calculated data for (3*S*,6*R*,8*S*,9*S*)-**5a** and (3*S*,6*S*,8*R*,9*R*)-**5b**.

		DP4+	86.42%	13.58%
Nuclei	sp2?	xperimenta	Isomer 1	Isomer 2
C	x	122.44	131.238	131.53
C	x	199.59	203.942	203.056
C		75.29	78.338	78.149
C	x	171.51	173.932	174.402
C	x	128.5	139.759	139.848
C		18.46	20.382	20.534
C		20.69	22.232	22.16
C		39.6	44.243	44.288
C		30.31	31.56	31.994
C		101.66	105.23	105.176
C		69.93	73.333	73.261
C		31.72	32.473	31.823
C		21.45	22.653	22.652
C		55.32	57.549	57.509

Figure S70. DP4+ probabilities (%) for conformers of (3*S*,6*R*,8*S*,9*S*)-**5a** (isomer 1) and (3*S*,6*S*,8*R*,9*R*)-**5b** (isomoer 2).

Table S12. Relative free energies[a] and equilibrium populations[b] of conformers for (3*S*,6*S*,8*R*,9*S*)-**6a** [c]

Conformer	Δ*G*	P (%)
**6a1**	0.00	68.40
**6a2**	0.72	20.24
**6a3**	1.06	11.35

[a] B3LYP/6-31G(d), in kcal/mol. [b] From Δ*G* values at 298.15 K. [c] in MeOH, no imaginary frequency.

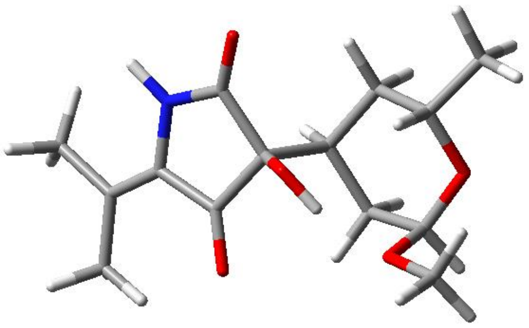

(3*S*,6*S*,8*R*,9*S*)-**6a1**

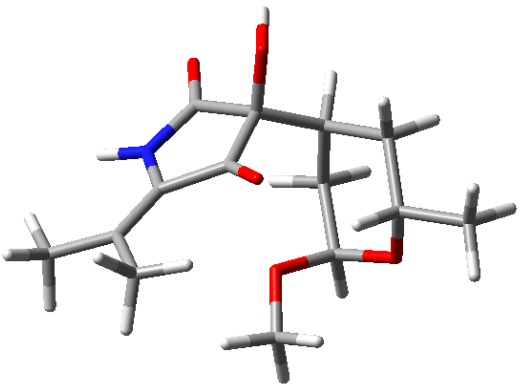

(3*S*,6*S*,8*R*,9*S*)-**6a2**

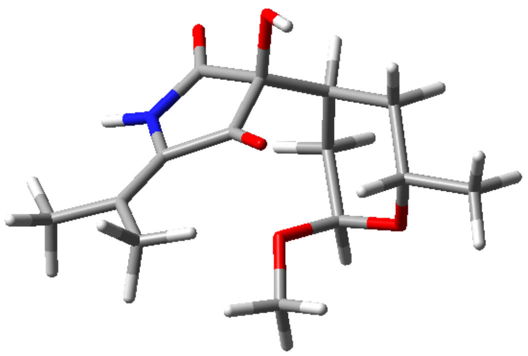

(3*S*,6*S*,8*R*,9*S*)-**6a3**

Figure S71. The optimized structure of conformers for (3*S*,6*S*,8*R*,9*S*)-**6a** in MeOH at B3LYP/6-31G (d) level.

Table S13. Relative free energies[a] and equilibrium populations[b] of conformers for (3*S*,6*R*,8*S*,9*R*)-**6b** [c]

Conformer	Δ*G*	P (%)
**6b1**	0.00	77.82
**6b2**	0.98	14.77
**6b3**	1.39	7.41

[a] B3LYP/6-31G(d), in kcal/mol. [b] From Δ*G* values at 298.15 K. [c] in MeOH, no imaginary frequency.

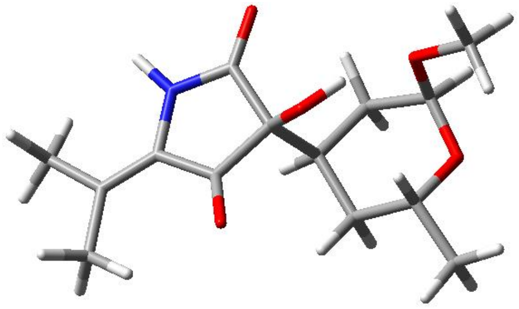

(3*S*,6*R*,8*S*,9*R*)-**6b1**

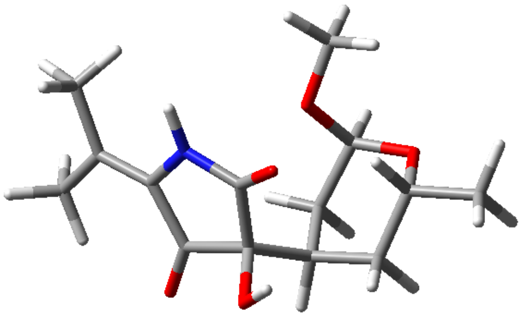

(3*S*,6*R*,8*S*,9*R*)-**6b2**

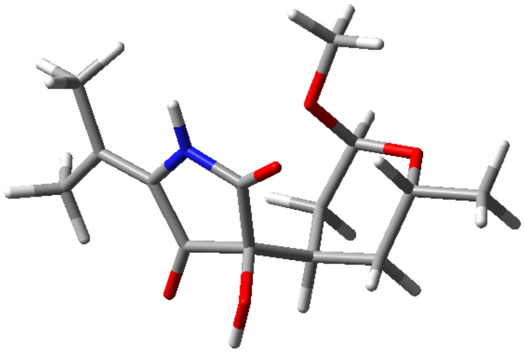

(3*S*,6*R*,8*S*,9*R*)-**6b3**

Figure S72. The optimized structure of conformers for (3*S*,6*R*,8*S*,9*R*)-**6b** in MeOH at B3LYP/6-31G (d) level.

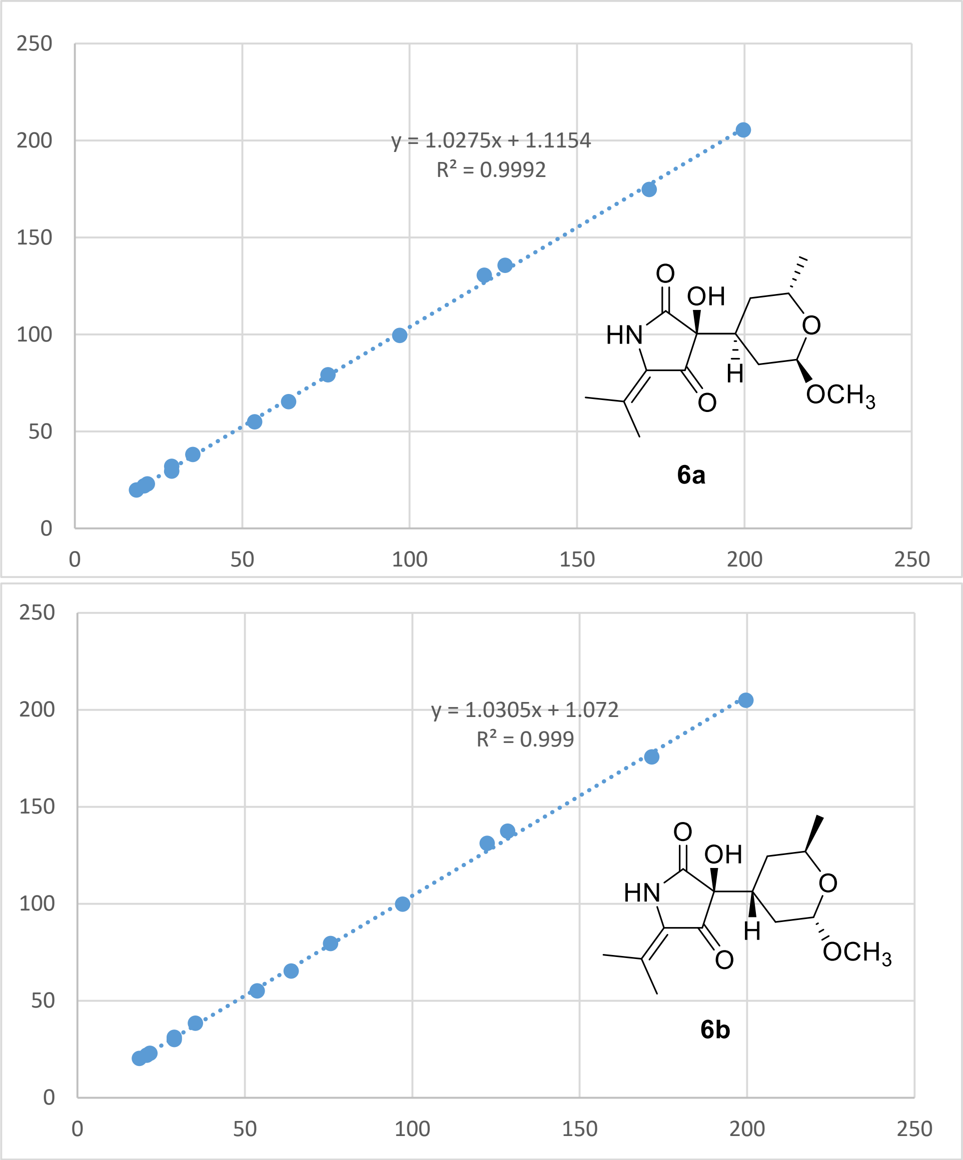

Figure S73. Correlation plots of experimental ^{13}C NMR chemical shifts versus the corresponding calculated data for (3*S*,6*S*,8*R*,9*S*)-**6a** and (3*S*,6*R*,8*S*,9*R*)-**6b**

		DP4+	86.61%	13.39%
Nuclei	sp2?	xperimenta	Isomer 1	Isomer 2
C	x	122.28	130.626	131.05
C	x	199.65	205.514	204.922
C		75.55	79.267	79.475
C	x	171.53	174.733	175.655
C	x	128.44	135.751	137.446
C		18.43	19.937	20.266
C		20.66	22.002	21.895
C		35.23	38.188	38.331
C		28.86	29.628	29.974
C		97.05	99.607	99.78
C		63.82	65.342	65.329
C		28.86	32.037	31.086
C		21.6	22.914	22.846
C		53.67	54.999	55.092

Figure S74. DP4+ probabilities (%) for conformers of (3*S*,6*S*,8*R*,9*S*)-**6a** (isomer 1) and (3*S*,6*R*,8*S*,9*R*)-**6b** (isomoer 2).

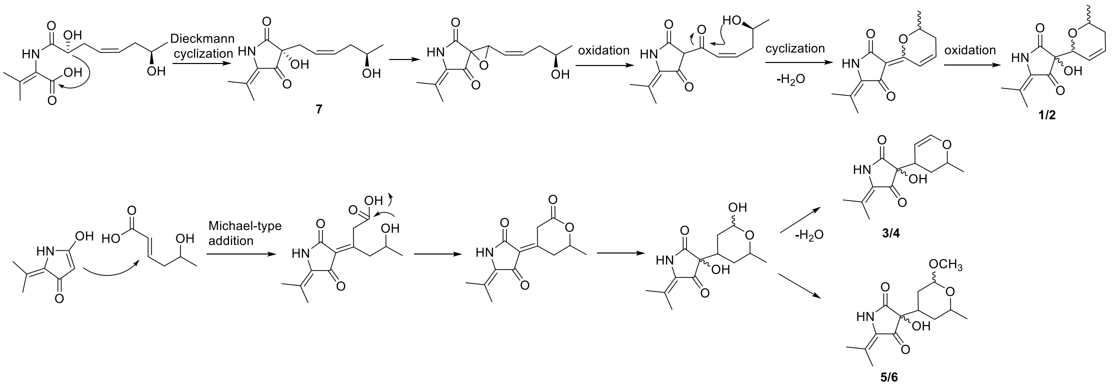

Figure S74. Proposed biosynthetic pathway of compounds **1–7** [1,2].

[1] Rischer, M.; Lee, S. R.; Eom, H. J.; Park, H. B.; Vollmer, J.; Kaster, A. K.; Shin, Y. H.; Oh, D. C.; Kim, K. H.; Beemelmanns, C. Spirocyclic cladosporicin A and cladosporiumins I and J from a Hydractinia-associated Cladosporium sphaerospermum SW67. *Org. Chem. Front.* **2019**, *6*, 1084-1093.

[2] Mo, X.; Gulder, T. Biosynthetic strategies for tetramic acid formation. *Nat. Prod. Rep*. **2021**, *38*, 1555-1566.
